# A Comprehensive Review of Modeling of Solid Oxide Fuel Cells: From Large Systems to Fine Electrodes

**DOI:** 10.1021/acs.chemrev.4c00614

**Published:** 2025-02-07

**Authors:** Zhen Wu, Pengfei Zhu, Yakun Huang, Jing Yao, Fusheng Yang, Zaoxiao Zhang, Meng Ni

**Affiliations:** † School of Chemical Engineering and Technology, Xi’an Jiaotong University, Xi’an 710049, China; ‡ State Key Laboratory of Multiphase Flow for Power Engineering, Xi’an Jiaotong University, Xi’an 710049, China; § Department of Building and Real Estate, Research Institute for Sustainable Urban Development & Research Institute for Smart Energy, Hong Kong Polytechnic University, Hung Hom, Kowloon, Hong Kong, 999077, China

## Abstract

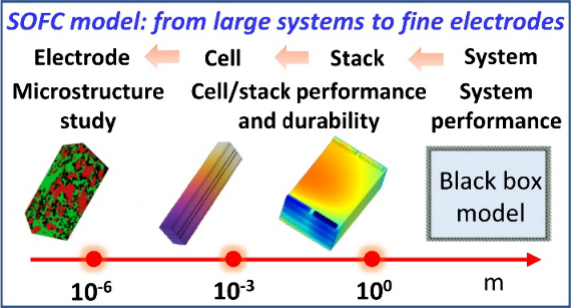

Commercialization of solid oxide fuel cell (SOFC) systems requires improved SOFC performance and durability, which is highly dependent on the coupling of the SOFC stack with other auxiliary components, SOFC stack configuration, and electrode microstructure. Optimization of SOFC systems at the system/stack/cell/electrode scale via experimentation is expensive and challenging, whereas numerical modeling can be fast and cost-effective. Although many excellent reviews on SOFCs have been published, the previous articles lack practical problem-oriented literature classification and do not cover new emerging models, such as artificial intelligence (AI) assisted models, heterogeneous models, and so on. These models are important for accelerating the solution of large-scale multiphysics models and describing mesoscopic electrode behaviors. In this review, a top-down approach is adopted that can truly guide SOFC system/stack/cell/electrode design to meet targeted applications. Another distinct feature of this review is the inclusion of the latest developments in SOFC modeling. This review offers a thorough summary and in-depth analysis of an extensive collection of research on SOFC simulations, classifying the models into distinct categories based on their varying scales, and serves as a valuable tool to assist researchers in selecting the most suitable models for diverse research objects.

## 1 Introduction

With the continuous development of the global economy, human demand for energy, as a crucial driver of social development, has increased. However, massive of fossil fuel use has led to environmental pollution, posing a substantial challenge that must be addressed by all countries together. The 26th United Nations Climate Change Conference held in Glasgow, United Kingdom, in November 2021, highlighted the “1.5 °C target,” which aims to ensure global net zero carbon emissions and control the temperature rise to within 1.5 °C by midcentury.^1^ China introduced its own “dual-carbon target” in 2021, aiming to achieve peak carbon dioxide (CO_2_) emissions by 2030 and carbon neutrality by 2060, thereby contributing to global temperature control.^2^ This unprecedented opportunity for environmental governance has induced more stringent requirements on the energy sector. Currently, there are two paths to reducing CO_2_ emissions in the energy sector: (1) from the supply side, clean energy should be utilized as much as possible and energy conversion efficiency should be improved; and (2) from the demand side, energy should be saved as much as possible and the efficiency of energy-consuming equipment should be improved.

As a representative clean energy, hydrogen (H_2_) is currently the focus of researchers and the energy sector worldwide. H_2_ can be directly utilized as fuel for clean power generation and clean transportation. Moreover, as a promising medium or carrier for energy storage, H_2_ can store excess electricity generated by other renewable energy sources, such as solar and wind energy, via water electrolysis. This approach provides a solution for the spatiotemporal inhomogeneity of renewable energy. Apart from water electrolysis, H_2_ can be produced via water splitting through solar thermochemical cycles, producing H_2_ and oxygen (O_2_) in two separate steps. In addition, H_2_ can be derived from secondary processing of fuels, such as coal and biomass gasification. Refining primary fuels can produce H_2_-rich syngas, which is an efficient and clean fuel with low CO_2_ emissions. In particular, biomass conversion to syngas is expected to create an energy supply without carbon emissions.

After realizing large-scale production of H_2_, especially green H_2_, its efficient utilization or of H_2_-containing syngas is the next challenge. One of the most promising ways to harness H_2_ is electricity generation through fuel cell technology, which offers numerous advantages, including high efficiency, cleanliness, low noise, and ease of maintenance.^3−5^ Importantly, fuel cell efficiency is not limited by the classical Carnot cycle, enabling efficiencies that are impossible to achieve with conventional heat engines or thermal cycles. The solid oxide fuel cell (SOFC), a type of high-temperature fuel cell, typically operates at approximately 800 °C.^6,7^ Additionally, SOFCs are fuel flexible, as most fuels can be chemically converted to H_2_ and carbon monoxide (CO)-rich syngas for subsequent electrochemical reactions at high temperature. Thus, the SOFC is well suited for the efficient, clean conversion of H_2_ or H_2_-containing syngas, making it an ideal candidate to replace conventional power generation technologies. The SOFC is regarded as the fourth power generation technology, following thermal power, hydropower, and nuclear power generation.^8^ Currently, SOFC systems are rapidly advancing toward large-scale application in countries such as Japan, South Korea, the United States, and various European nations. However, this will require improved SOFC performance and long-term stability, which depends on the coordination of the SOFC stack and other components and optimal design or operation of the SOFC stack/cell and electrode structure. For example, thermal stress caused by uneven temperature distribution may lead to cell delamination, crack or even failure. Further, hydrocarbon fuel decomposition leads to carbon deposition and performance degradation. SOFC stack performance depends not only on the stack configuration but also on the electrode microstructure, which depends on the morphology and material composition of the starting materials. Thus, the whole SOFC system must be optimized to achieve high performance and durable operation, including the stack, single cells in the stack, and the electrode microstructure. Unfortunately, optimizing the structure and operating conditions of the system/stack/cell/electrode experimentally is expensive and time-consuming. In a word, their high-temperature operation makes experimental studies on SOFCs economically and technically challenging. To address this limitation, numerical modeling of SOFCs has gradually developed and matured.

**Figure 1:**
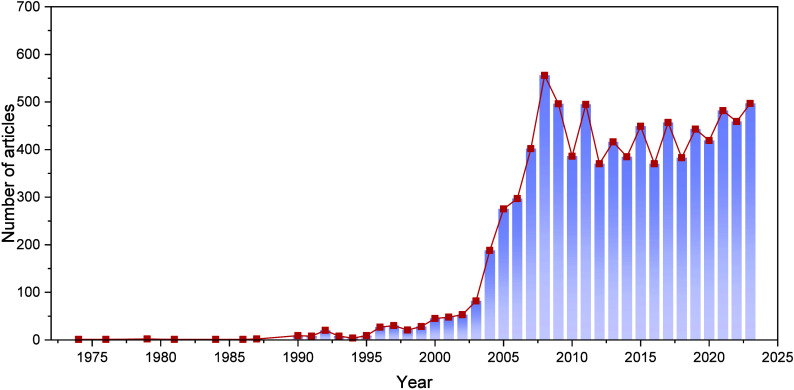
Number of articles published on SOFC modeling from 1974 to 2023. (Data from SCOPUS database, searching for the keywords (SOFC OR (Solid Oxide Fuel Cell) AND (Model OR Modeling) in the title, abstract, or keywords).

**Figure 2:**
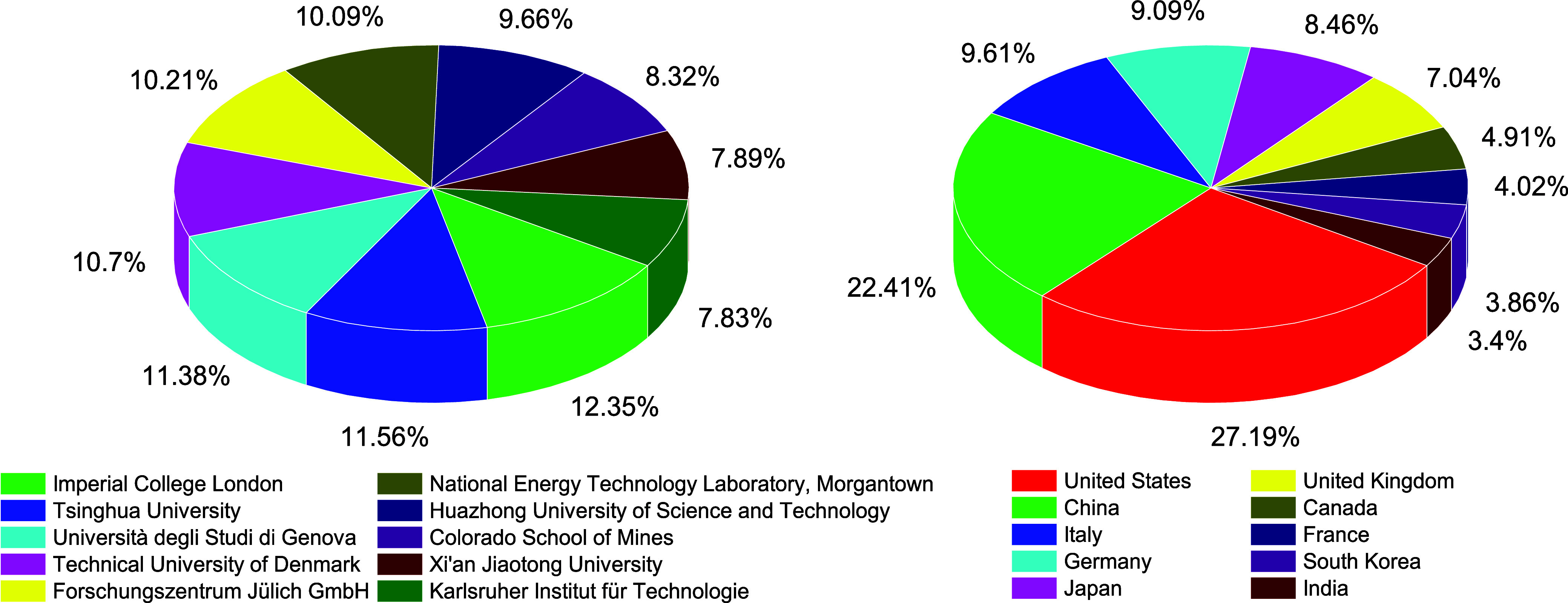
Comparison of the number of published articles on SOFC modeling by country and research institution. (Data from SCOPUS database, searching for the keywords (SOFC OR (Solid Oxide Fuel Cell) AND (Model OR Modeling) in the title, abstract, or keywords.)

As demonstrated in Figure 1, many articles on SOFC modeling have been published over the past decades, with >300 studies published annually between 2008 and 2022. This growth in the number of publications underscores the importance of numerical simulations in SOFC-related R&D. Figure 2 illustrates the number of articles on SOFC modeling published by different countries or research institutions, highlighting countries with considerable industrialization of SOFC technology, such as the United States, Japan, and various European countries. Additionally, Figure 2 also lists the top 10 research institutions that have published the most articles on SOFC modeling.

In summary, numerical modeling has become a crucial tool in the study of SOFCs, and SOFC numerical simulations have progressed rapidly in the last 20 years, leading to a wealth of research outcomes. Therefore, a comprehensive summary and review of SOFC numerical modeling is essential to understand the achievements and shortcomings of existing models, and several excellent reviews can be found in the literature.^3,9−12^ However, powder-to-power models were only recently developed to help identify the starting morphology and material composition of SOFCs for targeted performance and durability. This approach enables the top–down design of SOFCs. Moreover, the application of artificial intelligence (AI) has greatly facilitated SOFC design and optimization. Therefore, a comprehensive literature review of SOFC modeling in the past few years is warranted to avoid extensive repetitive work in the future and enhance numerical simulation use in SOFC-related R&D. To maintain focus, this review emphasizes macroscopic and mesoscopic reaction-transport-mechanical phenomena in SOFC numerical simulations, excluding studies performed at the micromolecular or atomic scale.

### 1.1 History and Development of SOFCs

SOFC technology moved from theory to practical application within a century. The famous physical chemist and Nobel Prize winner Nernst was the first scientist to develop an SOFC and one of the earliest SOFC pioneers.^3^ In 1899, Nernst accidentally discovered that zirconia doped with calcium, magnesium, or yttrium oxide could conduct O_2_ ions (O^2–^) at 500 °C–1400 °C and both O^2–^ ions and electrons at 1500 °C. Based on this discovery, Nernst invented the Nernst lamp.^13^ However, SOFC development stagnated until the 1950s as the high operating temperature limited the choice of possible electrode materials and practical application of SOFCs. During this period, the Swiss scientist Baur and his colleague Preis developed an SOFC using the electrolyte discovered by Nernst.^14^ They employed tubular YSZ (yttria-stabilized zirconia) as the electrolyte, iron or solid carbon as the anode, and Fe_3_O_4_ as the cathode to successfully fabricate an SOFC running on H_2_/CO as the fuel and air as the oxidant. The SOFC achieved a current density of up to 1 mA·cm^–2^ at 0.65 V and 1050 °C. In the 1940s, the Russian scientist Davtyan attempted to add phosphorus cerium lanthanum ore to a mixture of NaCO_3_, WO_3_, and sodium glass to prepare a solid electrolyte with high conductivity and mechanical strength.^15^ However, the electrolyte preparation process induced side reactions and the electrolyte life was short. The Dutch scientists Broers and Ketelaar subsequently discovered that the electrolyte prepared by Davtyan was molten at at 650 °C–700 °C, suggesting that finding a viable electrolyte was essential. A solid electrolyte with good CO tolerance was developed in the late 1950s, which greatly attracted the interest of researchers exploring high-temperature fuel cells. Westinghouse Electric committed to SOFC R&D in the late 1950s, focusing on the development of electrolyte materials based on zirconium compounds. Finally, Westinghouse Electric achieved a breakthrough in SOFC technology in the 1960s and 1970s and first manufactured a 5 kW SOFC generator consisting of 324 tubular single cells in 1986.^16^ In the following decades, research on SOFCs conducted by institutions worldwide has boomed, with researchers investigating the working mechanism, electrode material manufacture, stack amplification, and system integration.

**Figure 3:**
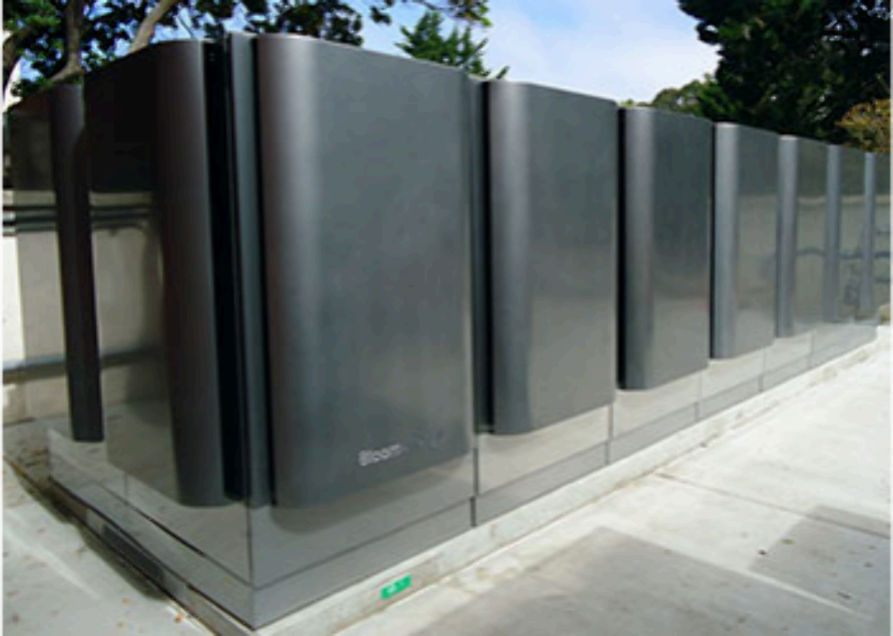
SOFC power plant for Adobe installed by Bloom Energy.^17^

SOFC-related research has rapidly developed. Several demonstration SOFC systems have been constructed, and many energy enterprises have begun selling SOFCs and offering installation services. For example, Bloom Energy, FuelCell Energy, and Japan’s NEDO (The New Energy and Industrial Technology Development Organization) ventured into the SOFC market. Bloom Energy has become an important manufacturer of large-scale commercial SOFC stacks, enabling commercial application of kilowatt-to-megawatt SOFC power generation systems. Today, Bloom Energy provides clean, reliable, and affordable energy to leading companies across a broad range of industries, such as Honda, Yahoo, Google, Walmart, etc.^17^Figure 3 presents an image of the SOFC power plant installed by Bloom Energy for Adobe at its San Jose headquarters and downtown San Francisco offices. Currently, Bloom Energy has installed two SOFC power plants for Adobe, with a cumulative installed capacity of approximately 1.2 MW. The Adobe SOFC power plant fueled by renewable biogas can produce 14,000 MWh of clean electricity per year. Adobe intends to reduce its carbon footprint by approximately 121.5 million pounds over 10 years, which is equivalent to the carbon emissions of 1,810 compact cars per year.

FuelCell Energy has primarily focused on stationary SOFC R&D. Its product is mainly used in combined heat and power (CHP) systems. NEDO primarily aims to improve the lifespan and reduce the cost of household CHP cogeneration systems. By 2030, NEDO envisions an SOFC hybrid system with a power generation efficiency of approximately 60%, operating time of 90,000 h, and cost of <100,000 JPY/kilowatt. Using current SOFC technology, the efficiency target has been preliminarily achieved; however, the cost and operation time targets need further research. To sum up, SOFC technology has transitioned from the laboratory to small-scale market applications after years of development. The latest information about the commercialization or marketization of SOFC can be found at the Web site.^18^

**Figure 4:**
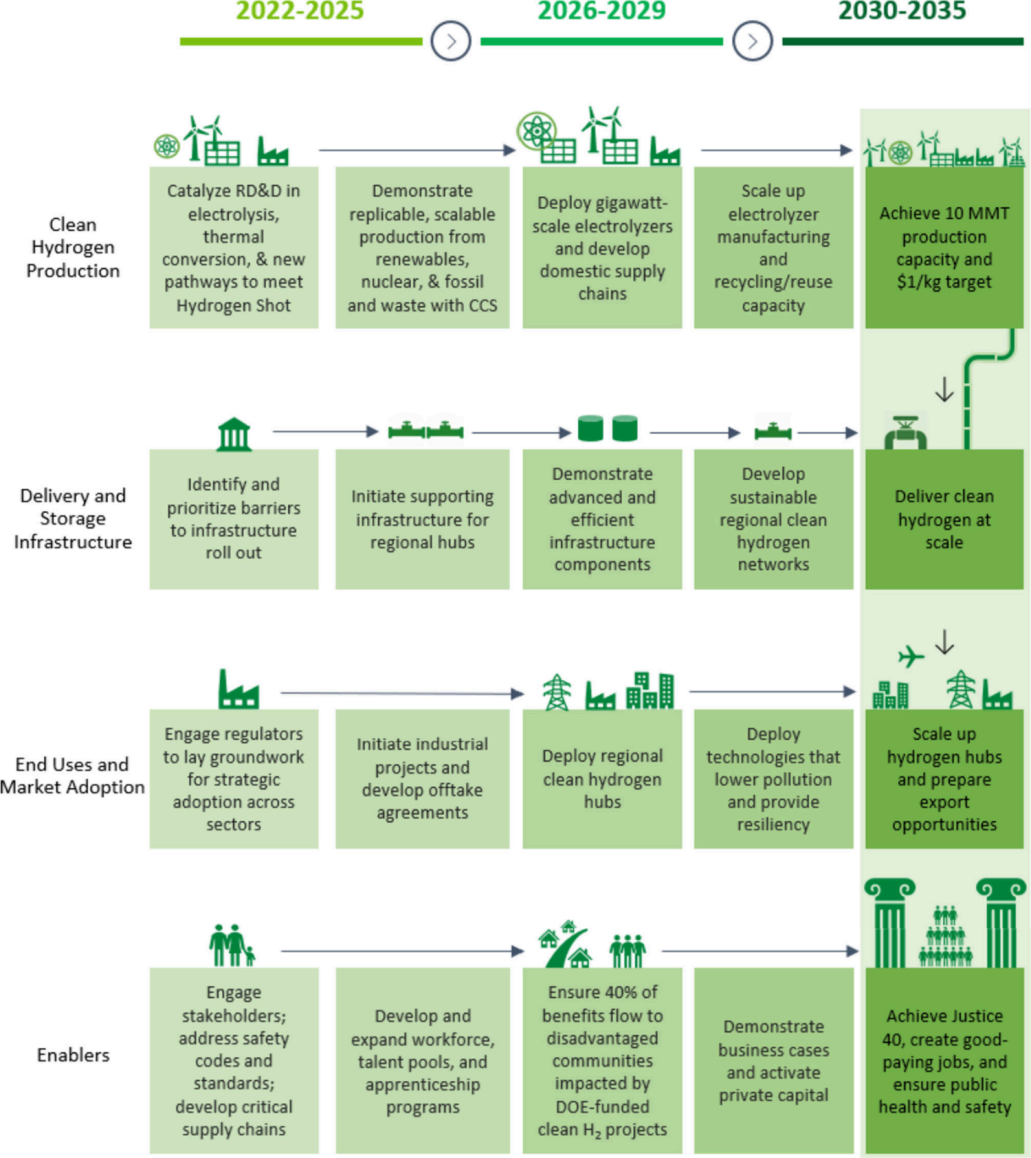
Timeline for the national action plan for clean H_2_ energy proposed by the U.S. Department of Energy.^19^

The U.S. Department of Energy released a “National Clean Hydrogen Strategy and Roadmap” in June 2023.^19^ The timeline of the national action plan for clean H_2_ energy is illustrated in Figure 4, indicating that the United States will begin to deploy regional clean H_2_ hubs in 2026. An H_2_ hub includes H_2_ power generation, the H_2_ chemical industry, H_2_ transportation, and other H_2_ energy industry chains, in which SOFCs will play an important role. In particular, owing to their efficient and clean power generation performance, SOFCs are an attractive application prospect in the stationary power generation scenario.

**Figure 5:**
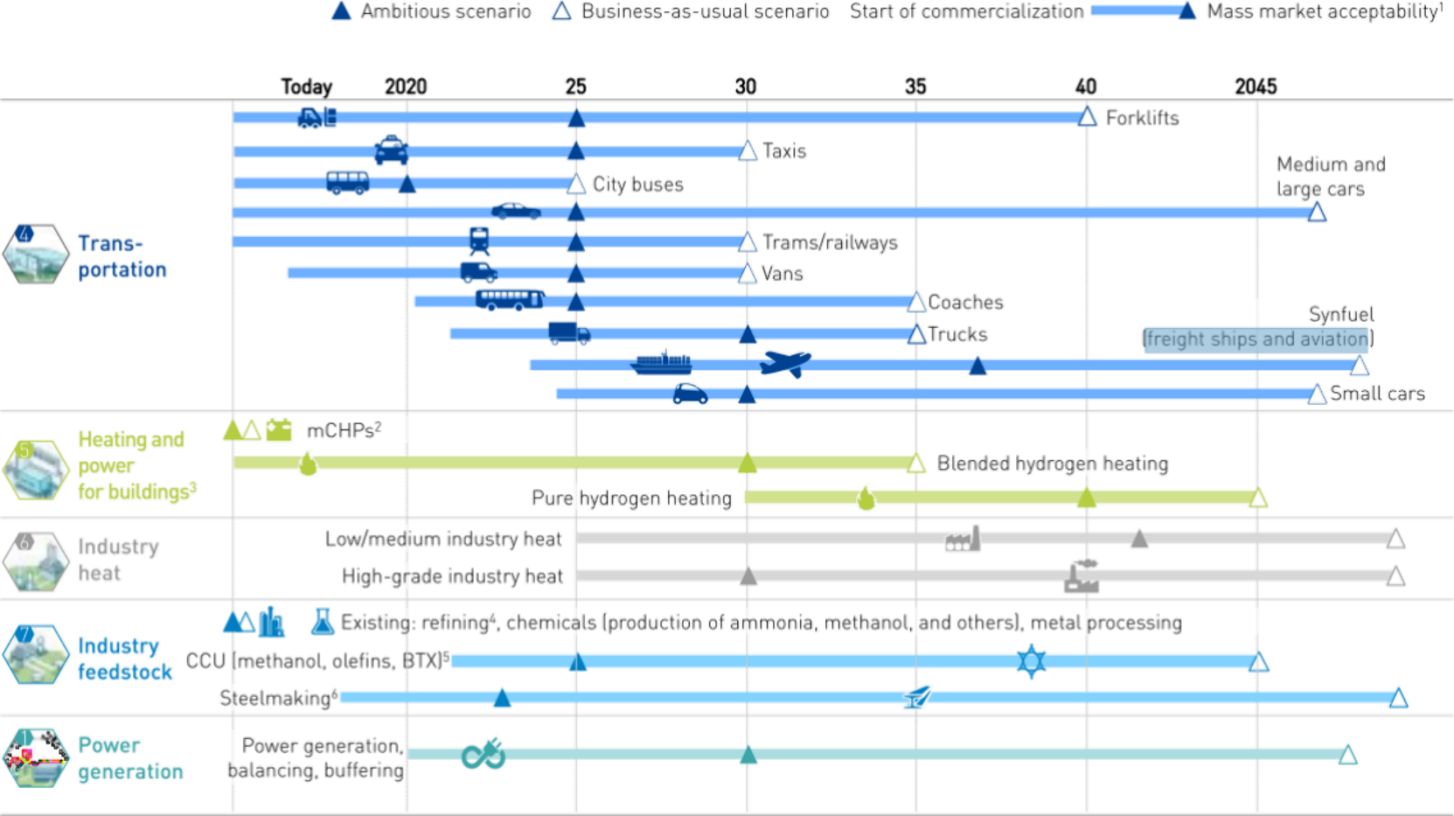
European Union’s H_2_ energy development roadmap proposed by the Fuel Cells and Hydrogen Joint Undertaking.^20^

The European Union proposed a H_2_ energy roadmap in 2019, as shown in Figure 5,^20^ which emphasizes the application and development of H_2_ in the fields of transportation, heating and power for buildings, industrial heating, industrial feedstock, and power generation. SOFCs show great application potential in H_2_-powered freight ships and aviation, building cogeneration systems, industrial heating, and distributed power production scenarios. In addition, Japan and China released H_2_ energy development roadmaps in 2019^21^ and 2023,^22^ respectively. These roadmaps share the great application potential of SOFCs in H_2_ utilization scenarios.

### 1.2 Working Principle of SOFCs

**Figure 6:**
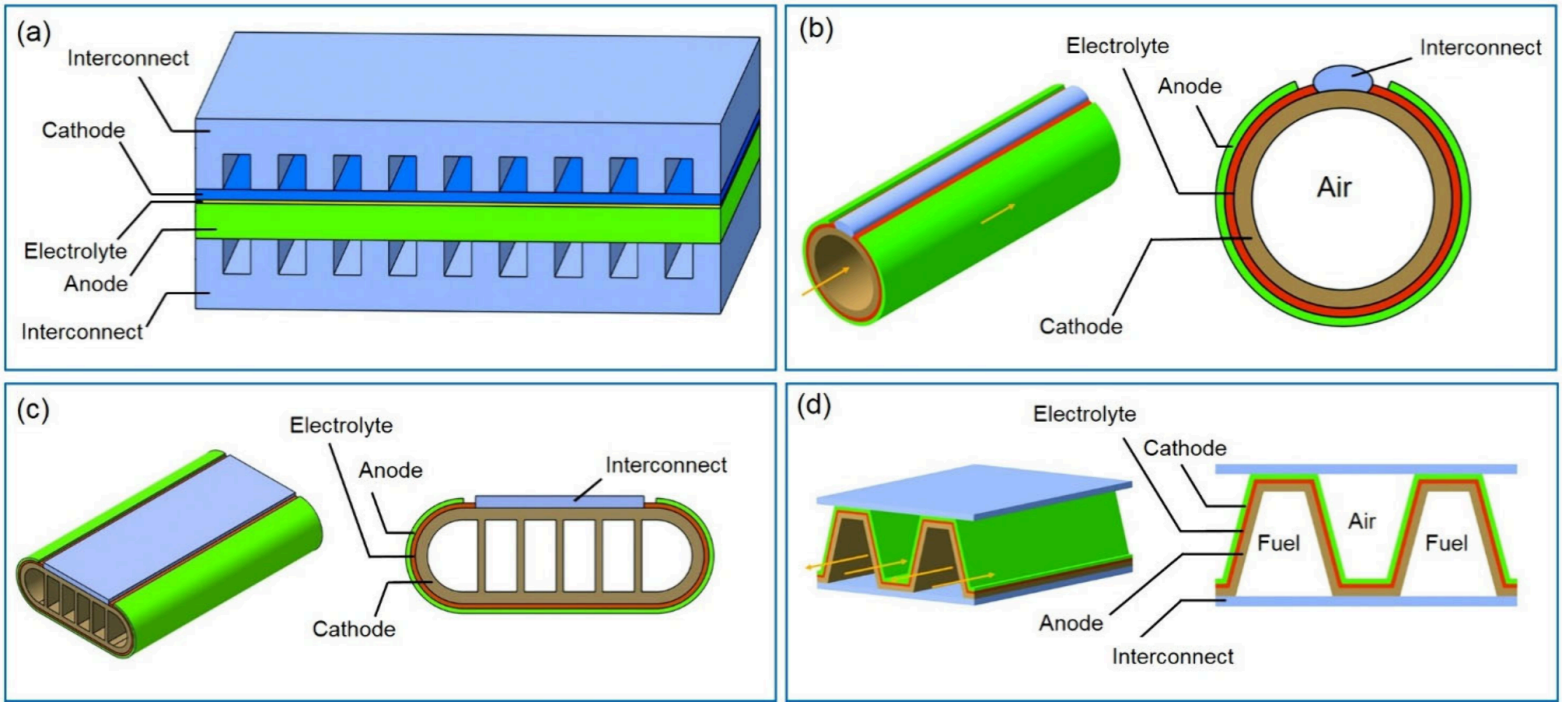
Schematic of different SOFC structures: (a) planar SOFC structure; (b) tubular SOFC structure; (c) flat tubular SOFC structure; (d) MOLB-type SOFC structure. Redrawn and modified with permission from ref (26). Copyright 2021 *Chemistry Bulletin* (Chinese).

The process of power generation in SOFCs is realized through electrochemical reactions. An SOFC comprises a cathode, anode, and electrolyte, which are closely combined via sintering to form a sandwiched positive electrode/electrolyte/negative electrode (PEN) structure. Current mainstream SOFC structures can be divided into four types based on the different supply modes of anode fuel and cathode air: planar, tubular, flat tubular, and monoblock-layer build–type (MOLB-type), as illustrated in Figure 6. Planar SOFCs have the advantages of simple manufacture, low cost, and high power density, although they have high sealing requirements.^23^ Thus, planar SOFCs currently dominate the stationary power generation scenario. Tubular SOFCs are robust with easy gas sealing; however, their performance is unsatisfactory owing to high ohmic loss due to electronic conduction and they have relatively high fabrication costs.^24^ Inspired by the advantages of planar and tubular SOFCs, Westinghouse Electric combined their characteristics to produce a novel flat tubular SOFC, which can achieve high power density at high temperatures.^25^ MOLB-type SOFCs, inspired by planar SOFCs, have also been developed. The effective working area of MOLB-type SOFCs is increased compared to that of planar and tubular SOFCs owing to their wavy PEN structure, which reduces internal resistance, improves the volumetric power density, and avoids the need for high-temperature sealing materials. However, the manufacturing process of MOLB-type SOFCs is complex.

Table 1 summarizes the characteristics of different SOFC structures and compares their performance.

**Table 1:** Characteristics and Performance of Different SOFC Structures

criterion	planar	tubular	flat tubular	MOLB-type
Manufacture	• Low cost	• High cost	• High cost	• High cost
	• Poor sealing	• Good sealing	• Good sealing	• Good sealing
	• Simple interconnector	• Complex interconnector	• Simple interconnector	• Simple interconnector
	• Low reliability	• High reliability	• Slightly low reliability	• High reliability
Durability performance	• Poor thermal cycling stability	• Good thermal cycling stability	• Slightly poor thermal cycling stability	• Poor thermal cycling stability
	• Low long-term stability	• High long-term stability	• High long-term stability	• High long-term stability
	• High performance attenuation rate	• Low performance attenuation rate	• Slightly low performance attenuation rate	• High performance attenuation rate
	• Slow start-up	• Fast start-up	• Slow start-up	• Slow start-up
Electrochemical performance	• Low internal resistance	• High internal resistance	• Low internal resistance	• Low internal resistance
	• High power density	• Low power density	• High power density	• High power density
	• High current density	• Slightly low current density	• High current density	• High current density

**Figure 7:**
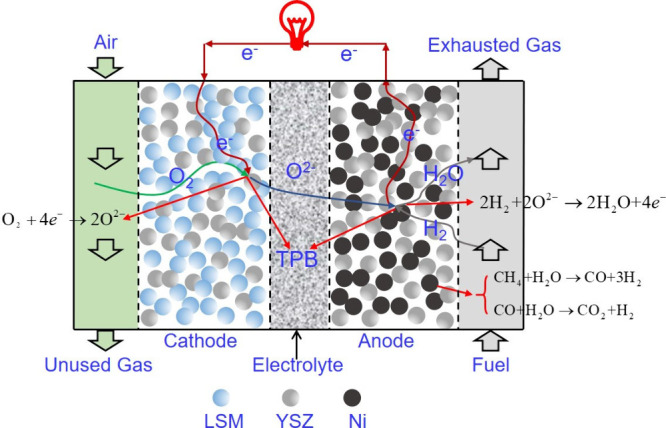
Schematic of the working mechanism of SOFCs.

In addition to the aforementioned SOFC structures, other SOFC structures have emerged in recent years to further enhance SOFC performance. Regardless of SOFC structure optimization, the PEN configuration remains at the core of SOFCs. The primary working mechanism of the PEN configuration is illustrated in Figure 7. In a typical SOFC, the porous anode is usually Ni–YSZ, while the classical cathode material is strontium-doped lanthanum Manganite (LSM-YSZ), which has high catalytic activity at high temperature for electronic conduction and the O_2_ reduction reaction. However, the O_2_ reduction activity of LSM significantly decreases with decreasing temperature, and its polarization resistance is greatly increased at low temperature. Thus, LSCF (La_1–*x*_Sr_*x*_Co_1–*y*_Fe_*y*_O_3−δ_) has become a typical material employed in SOFC cathodes owing to its high ionic conductivity and ability to substantially reduce the thermal expansion coefficient.^27^ The electrolyte is usually made of YSZ. The solid electrolyte can conduct O^2–^ at high temperatures and prevent direct contact between the fuel and O_2_. The anode and cathode are separated by the solid electrolyte, thereby preventing short circuiting. The O_2_ transported through the cathode channel diffuses to the interior of the porous cathode, while electrons are captured by O_2_ at the triple-phase boundary (TPB) and oxidized to generate O^2–^. The half electrode reaction of the cathode is described by eq 1, as follows:
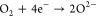
1

Driven by the potential difference and concentration gradient, O^2–^ generated at the cathode migrates to the TPB of the anode through the electrolyte, where it combines with fuel molecules that have diffused from the anode, releasing electrons and forming steam or CO_2_. In principle, all combustible fuels can be electrochemically oxidized for power generation due to the transport of O^2–^ to the SOFC anode through the electrolyte. However, in practice, breaking the C–H or C–C bond is difficult, making the electrochemical oxidation of hydrocarbon fuels challenging. Thus, H_2_ is usually electrochemically oxidized in SOFCs, as expressed by eq 2. The electrochemical oxidation rate of H_2_ is reportedly 2.3–3.1 times higher than that of CO at 800 °C,^28^ indicating that the electrochemical reaction of CO, as expressed by eq 3, should be considered in SOFCs. However, the electrochemical oxidation of CO was not considered in early modeling studies of SOFCs for two main reasons:^29,30^ (1) the water–gas shift (WGS) reaction rate is faster than the electrochemical oxidation of CO; and (2) accurate and reliable models for simulating the electrochemical oxidation of CO were lacking. Nevertheless, considering the oxidation reaction of CO is recommended for accurate simulation of SOFCs.^31−33^ When the fuel for SOFCs is hydrocarbons,^34,35^ alcohol,^33,36,37^ ammonia (NH_3_),^38−40^ or even solid fuel,^41−43^ it is often first converted into syngas via reforming for subsequent electrochemical reactions. An excellent review of SOFCs running on various alternative fuels can be found in ref (4). After the oxidation reaction of H_2_ or CO, the generated H_2_O and CO_2_ diffuse from the TPB of the porous anode to the anode channel, where they are removed from the SOFC stack. The electrons produced at the anode are collected by the interconnector and flow to the cathode via the external circuit, generating useful electrical power. At the TPB of the cathode, electrons combine with O_2_ to form O^2–^. The transportation of gas in porous electrodes, conduction of O^2–^ in the cathode, electrolyte, and anode, conduction of electrons in the electrode and current collector, and electrochemical reaction processes all considerably influence the distributions of gas concentration, temperature, current, and other parameters, ultimately affecting the output current density and voltage of SOFCs. Moreover, if the SOFC is coupled with internal reforming or WGS reactions, the characteristics of these reactions crucially affect the output performance of the SOFC. SOFC models are primarily established based on the aforementioned working mechanism.
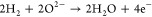
2
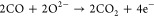
3

### 1.3 Current Challenges of SOFCs

Currently, the comprehensive demands for SOFCs may be broadly delineated into three fundamental aspects: (1) elevated efficiency, (2) optimal stability, and (3) economic cost. High temperatures cause coarsening of the Ni catalyst, leading to lower TPB and SOFC performance or even SOFC failure. Another challenge associated with high operating temperatures is a mismatch between the thermal expansion coefficients of the electrodes and electrolyte, which results in high thermal stress at the electrode–electrolyte interface, especially during start-up and shutdown. High thermal stress can cause cracks/delamination at the interface, substantially reducing the performance and lifespan of the SOFC. More importantly, high-temperature operation is one of the main reasons for the high cost of SOFCs as it requires high-temperature aging resistance of various components, materials, and coatings, increasing the costs of materials and processes. The balance of plant (BoP) components for SOFC stacks also need to operate at high temperatures, further driving up the costs of SOFC systems. Lowering the operating temperature can reduce SOFC-related material requirements and thermal management costs, thereby lowering the overall cost of SOFCs. Moreover, reducing the operating temperature has advantages, such as improving the stability and lifespan of SOFCs, hastening the start-up of the SOFC stack, and improving the open-circuit voltage (OCV) of the SOFC. However, the ion conductivity of the electrolyte will be greatly reduced at decreased operating temperatures, increasing ohmic loss of the electrolyte, and the catalytic activity of the electrode will also be greatly reduced, increasing polarization loss. In short, reducing the operating temperature contributes to lower cost and higher stability but it reduces the electricity production performance. Therefore, the development of electrolyte and electrode materials that can perform efficiently at lower temperatures is urgently needed. Reducing the operating temperature of SOFCs from 800 °C–1000 to 300 °C–500 °C while maintaining favorable electrode reaction kinetics and high ionic conductivity is a key goal of ceramic fuel cells.^44^ To date, the operating temperature of SOFCs can be lowered at most to 500 °C via optimization and modification of the solid electrolyte.^45^

Apart from new material development, optimizing the configuration and operating conditions of SOFC systems, the structure and operating conditions of the stack/cell, and electrode micromorphology play important roles in improving the efficiency and stability of SOFCs. The efficient electricity production target can be explored from two aspects: (1) conduct in-depth research on the working mechanism of SOFCs to guide manufacturing or optimization of the operating conditions for efficient power generation; and (2) couple SOFCs with other energy recovery devices through thermal network integration to improve the energy conversion efficiency of the whole system. In terms of stability, current research mainly focuses on clarifying the mechanisms responsible for SOFC performance degradation and developing reasonable measures to avoid or alleviate degradation. During the practical operation of SOFCs, all components may suffer damage, including the anode, cathode, electrolyte, sealant, and interconnector, which may lead to performance degradation. Therefore, exploring and evaluating the damage mechanisms of SOFCs can help to identify the types of damage that have the greatest impact on performance, so they can be addressed first. Golkhatmi et al.^46^ reviewed and summarized the latest research related to SOFC durability, including their failure mechanisms and study tools.

### 1.4 Contribution of Modeling to SOFC-Related Research

**Figure 8:**
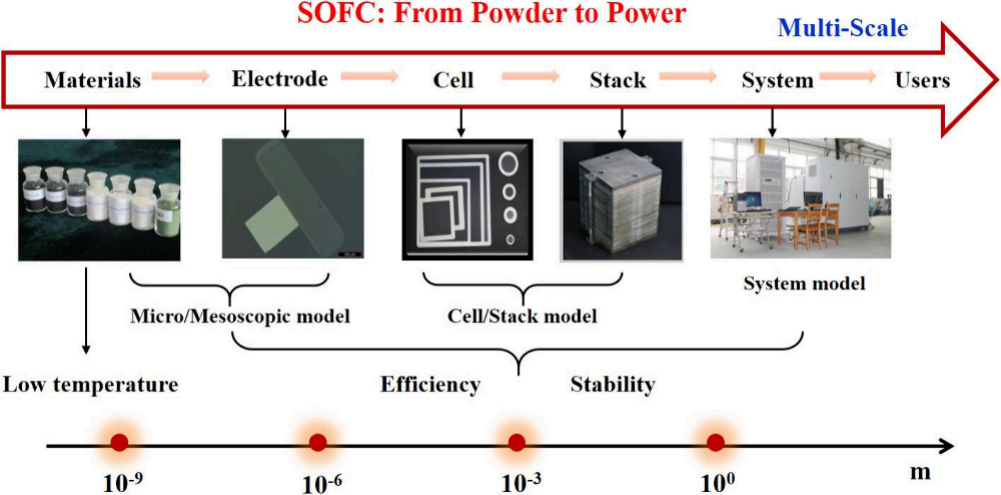
Relationship between research objects, research scale, and modeling methods for SOFCs.

At present, the ultimate goal of SOFC-related research is to develop SOFC systems that can operate efficiently and stably. The SOFC system comprises the SOFC stack and other BoP components, all of which need to cooperate thermodynamically to achieve efficient and clean energy conversion. Moreover, the system configuration needs to be reasonable and feasible, and the operating conditions need to be dynamically optimized in real time. These tasks can only be accomplished through system-scale simulation and optimization. Furthermore, efficient and stable operation of the system depends on SOFC performance and durability to a large extent. Optimal design at the stack/cell scale is required to achieve an SOFC stack with high efficiency and stability. Nevertheless, studying the evolution of electrode morphology and key mesoscopic parameters at the cell/stack scale is challenging, and mesoscopic investigations can solve the difficult problems at the electrode scale. The electrochemical reactions, which take place in the heterogeneous porous electrode, are coupled with complex anode chemical reactions and the phenomena of mass and heat transfer in the porous media. Thus, exploring the complex reaction-transport phenomenon inside the electrode at the mesoscale is necessary to improve SOFC performance and durability. The development of high-performance and durable SOFC systems requires multiscale design and optimization, as demonstrated in Figure 8.

The main purpose of “low-temperature” SOFC-related research is to identify suitable electrolyte materials that can maintain high ionic conductivity and electrode materials with high reaction activity at low temperature. The most common approach is to screen low-temperature materials with excellent performance via atomic-scale numerical simulations (usually high throughput computations and machine learning method), followed by experimental validation and confirmation. System-scale research only needs to consider macroscopic electricity production performance without involving the more detailed internal reaction-transport mechanisms, mainly focusing on the targets of high efficiency and durability. When the SOFC system is in a dynamic working state, other BoP will have great influence on the stability of the stack. A system-scale control model needs to be developed to ensure that the stack can operate in a stable environment, thus demonstrating improved durability. However, system control models, which involve the coupling of multiphysics simulation and control strategy, are difficult to establish and time-consuming to solve. Additionally, there is a trade-off between simulation accuracy and computional load. Stack/cell research provides a detailed and in-depth description of the reaction-transport mechanisms, generally focusing on the targets of high efficiency and durability. Electrode research is more concerned with the reactions or structure at the mesoscale and is mainly focused on the degradation or mesoscopic reaction-transport mechanism.

**Figure 9:**
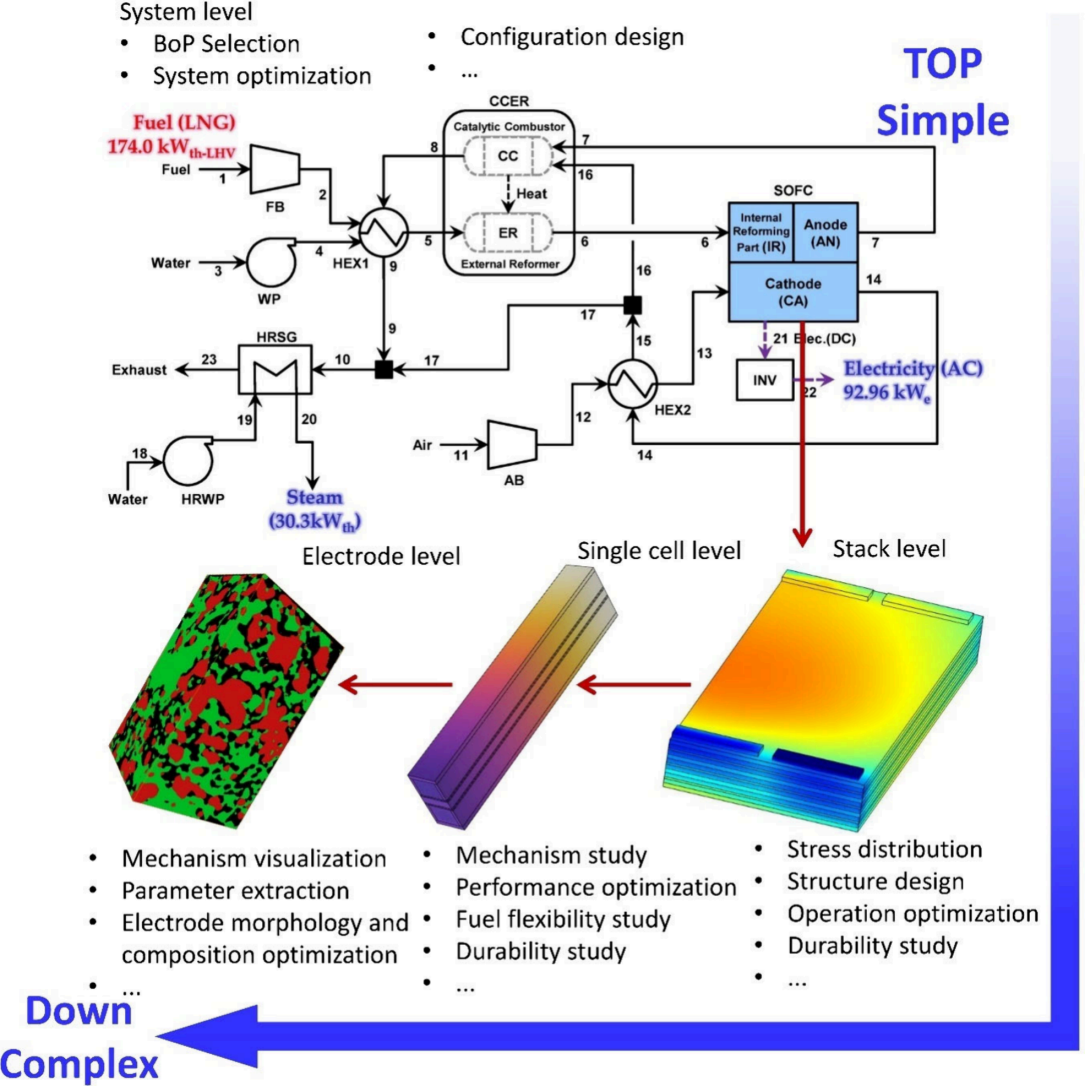
Schematic of the top–down approach for reviewing multiscale SOFC models. Reproduced with permission from ref (47). Copyright 2018 Elsevier.

The organization of this review adheres to the principle of progressing from simplicity to complexity, moving from broader systems to the intricate details of fine electrodes. This top–down approach facilitates explaining the development and application scope of SOFC models. SOFC system performance and durability are the ultimate targets. Based on the top–down approach, the initial particle morphology and composition corresponding to optimum SOFC performance and stability can be determined at the mesoscale. This is helpful for developing high-performance cells/stacks/systems because SOFC performance and durability depend on the initial particle morphology and composition. Thus, this approach is suitable for guiding SOFC development toward the desired performance and durability. By contrast, a bottom–up approach begins with the starting materials and composition to predict SOFC performance, making it difficult to achieve the desired performance and durability. Furthermore, the logical progression from simple to complex is appropriate for modeling or theoretical research and facilitates an understanding of the complex working principle and modeling process of SOFCs. Figure 9 illustrates the top–down approach for reviewing multiscale SOFC models.

### 1.5 Research Gap and Review Necessity

Over the past 20 years, the field of SOFC numerical simulation has experienced rapid growth, and numerous related studies have been published. Unsurprisingly, SOFC numerical simulations have been previously reviewed and summarized.For example, Kakac et al.^11^ summarized and reviewed SOFC multiphysics simulation research in 2007, providing a detailed description of transport models and the corresponding simulation work. However, research on SOFC multiphysics simulations before 2007 was relatively limited and only in the early stages of development. Therefore, such reviews cannot reflect recent progress in SOFC simulation work. Similarly, Martin et al.^12^ summarized and reviewed multiphysics field transport process modeling of SOFCs in 2010, introducing detailed mathematical models and classification of relevant models at the micro-, meso-, and macro-scales; however, these authors did not sufficiently analyze individual studies, summarize different numerical simulation topics, or point out subsequent development directions for these topics. Peksen^9^ reviewed studies related to the thermomechanical behaviors of SOFCs in 2015 but did not cover other simulation topics. Meanwhile, Cheng et al.^10^ provided a detailed review of macroscopic SOFC modeling and model-based control of SOFC-gas turbine (GT) systems in 2018. These authors provided a detailed summary of SOFC mathematical models, particularly focusing on reaction kinetics models, which are often contentious. However, their model classification did not effectively capture the problems or topics to be studied through simulations. More recently, Beale et al.^3^ summarized and reviewed SOFC continuous-scale models and complementary experiments in 2021, providing a detailed summary of the fundamental thermodynamics, reaction processes, transfer processes, porous electrode microstructural analysis, and overpotential. These authors also summarized the details of supplementary experiments related to SOFC simulations. Overall, this review is informative and provides a comprehensive summary of various details in SOFC continuous-scale modeling that are relevant to SOFC model development. The review literature on SOFC modeling has been summarized in Table 2.

**Table 2:** Summary and Key Points of Previous SOFC Simulation Related Works

year	main points	limitations	ref
2007	Provide a detailed description of transport models and corresponding simulation work.	This review focuses on the cell/stack scale transport model and does not include system scale and mesoscopic electrode scale models. In addition, due to the early publication of the review, it did not include the coupled AI model, elementary reaction model, heterogeneous multiphysical field model, phase field model, and other new emerging models.	(11)
2010	Review the detailed mathematical models and classify relevant models into micro, mesoscopic, and macro models.	This review does not cover system-level models and does not provide a detailed analysis of individual works. Similarly, this review does not include AI coupling models, heterogeneous multiphysics model, phase field model and Peridynamic model.	(12)
2015	Review works related to the thermo-mechanical behaviors of SOFC in detail but did not cover other simulation topics.	The thermo-mechanical model summarized in this review is only a specific application of the cell/stack scale model and can only solve problems related to thermo-mechanical behavior.	(9)
2018	Provide a detailed review of macroscopic modeling of SOFC and model-based control of SOFC-GT systems.	The model classification did not effectively capture the problems to be studied through simulations.	(10)
2021	Summarize the SOFC continuum scale models and complementary experiments.	This work does not include system scale models, AI coupling model, phase field model, and peridynamic model. The AI-coupled model can accelerate the solving speed and facilitate the fast prediction and optimization of SOFC performance. Other novel models are important for describing the behavior of mesoscopic electrode scales.	(3)

Although previous reviews have summarized and reviewed SOFC modeling efforts, few reviews on application-oriented SOFC modeling have been published. Moreover, such reviews do not cover rapidly evolving SOFC simulation–related studies conducted in recent years. Peksen’s review^9^ mainly covered SOFC thermomechanical models and did not summarize modeling research on other aspects of SOFCs. The authors of the last two articles, published in recent years, discussed the details of SOFC modeling, including disputed problems, which is valuable for SOFC model development. However, the lack of practical problem-oriented literature classification and review weakens their usefulness in practical SOFC development. From the perspective of SOFC development, SOFCs at the electrode/cell/system scale face distinct challenges. Achieving a stable and efficient SOFC power generation system requires solving problems at all scales. Currently, considerable numerical simulation research has been performed at the SOFC electrode/cell/system scale. Therefore, this review divides SOFC modeling into three scales and summarizes previous research performed at each scale. SOFC simulation studies conducted at various scales are interconnected, mutually reinforcing and collectively driving the advancement of SOFC technology. Thermodynamic simulations at the system scale primarily focus on determining the system configuration, operating conditions, and performance potential. The operating conditions of the system provide boundary conditions for cell/stack simulations, while multiphysics simulations at the cell/stack scale ensure efficient and stable operation of the system. Electrode simulations inform cell/stack simulations with critical parameters and degradation mechanisms. Cell/stack simulations, in turn, leverage the mesoscopic insights gained from electrode simulations to conduct more reliable simulations.

The literature classification is based upon the problems or purposes that the simulations aimed to solve. For instance, in Section 3.2.1, the published literature is divided into four topics: heat and mass transfer mechanisms, structural optimization, fuel flexibility, and stress distribution. This classification helps to clearly describe the development status of numerical simulations under each topic and point out research directions to promote SOFC development. Subsequently, this review suggests follow-up research directions and identifies problems that have not been previously solved. Since previous reviews have extensively discussed the details of SOFC models, this review focuses on providing the model frameworks, with the model details available in refs (3 and 10).

## 2 Development of SOFC System Models

### 2.1 Overview of the SOFC System Level Model

To maintain preferable performance and electricity production efficiency, SOFC fuel utilization is generally maintained at 70%–80%^48,49^ and the operating temperature is usually relatively high, approximately 800 °C. This means that the exhaust gas from SOFCs typically contains high-grade thermal energy and unused chemical energy. An effective approach to improving SOFC efficiency is to recover the energy from exhaust gas in a cascading manner. In recent years, efforts have been undertaken to integrate SOFCs with various thermal cycles or equipment to enhance the overall energy conversion efficiency. Currently, SOFCs are commonly combined with GTs,^50−52^ engine,^53−55^ proton-exchange membrane fuel cells (PEMFCs),^56−58^ the organic Rankine cycle (ORC),^59−61^ CO_2_ transcritical cycles,^62−64^ and others to recover the energy from exhaust gas. System-scale research mainly explores the performance potential of SOFC advanced energy systems, confirming the superiority and development potential of SOFC systems. Moreover, system studies provide the operating and boundary conditions for stack/cell investigations. For this purpose, the key steps of system studies include proposing or designing novel SOFC hybrid systems, conducting thermodynamic modeling and obtaining the operating state, and evaluating and optimizing system performance from thermodynamic and techno-economic perspectives. As a result, the primary requirement of system studies lies in the establishment of a well-founded and dependable thermodynamic model of SOFC hybrid systems.

The SOFC is almost the only electricity production equipment in SOFC systems, thus undertaking most of the electricity production work. Therefore, SOFC modeling and analysis directly affect the electricity production performance of the whole system. Furthermore, the working process of SOFCs, as a new electricity production technology, is complex and the corresponding models are not specific. As a result, SOFC models play a crucial role in modeling the overall system. Most SOFC system models are lumped parameter models, which are based on the principles of energy and mass conservation. Such models are convenient for providing a solution even if they do not include precise physics field information. Additionally, spatial distribution models have been developed for SOFC system simulations,^65−70^ providing insight into the spatial distributions of important parameters. However, these models are complex and their solution processes are difficult. There are two reasons to classify zero-dimensional (0D) and spatial distribution models as system models. First, both types of models are useful in SOFC-based system analysis and research, as shown in the “Model type” column in Table 4. The purpose of these models is to evaluate the electricity generation performance or analyze the specific parameter distribution under a given system configuration and operating conditions, not to analyze the performance of the whole stack/cell, conduct structural optimization, or solve other specific detailed problems inside the stack/cell. These problems are of concern to stack/cell models, which are described in Section 3. Second, the difference between parameter distribution and stack/cell models is that the former only considers the spatial distribution of temperature or concentration in a certain direction, while the latter is a typical multiphysics coupling model that fully considers the spatial distributions of all parameters within the entire stack/cell. In fact, the spatial distribution model serves as a simplification of the stack/cell multiphysics field model, considering that stack/cell models are difficult to solve quickly and efficiently in specific system applications. The BoP components commonly employed to assist SOFCs include heat exchangers, blowers, prereformers, and other standard chemical equipment, with well-established models. Given that this review focuses on the SOFC itself, only SOFC models are discussed and a detailed introduction to BoP component models is not provided. BoP component models have been extensively described in ref.^10^ This section summarizes SOFC system models and reviews research progress in the field.

### 2.2 Research Progress of System Models

#### 2.2.1 SOFC Models in Different System Configurations

**Figure 10:**
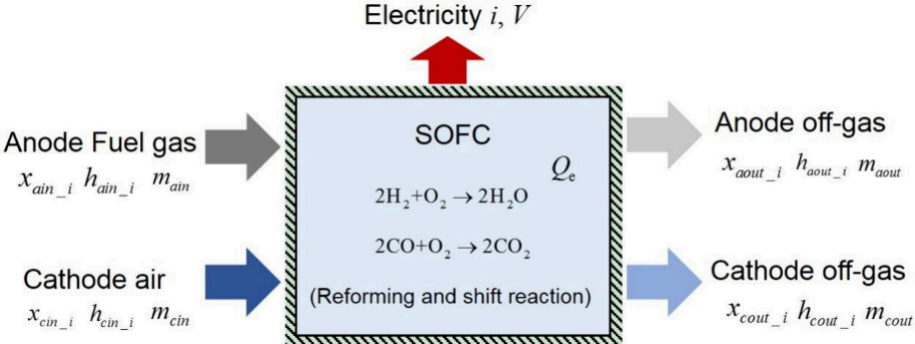
Schematic of a black box model for SOFCs.

A black box model with lumped parameters (also known as a thermodynamic equilibrium model) is the most popular approach for evaluating the energy conversion performance of SOFC hybrid systems. This model captures temperature and species concentrations only at the SOFC outlet and lacks detailed information inside the SOFC. While simple and easy to establish, the black box model (depicted in Figure 10) is limited to steady-state conditions owing to its relatively coarse modeling approach. Several key assumptions underlie its construction, including: (1) the SOFC is highly thermally insulated, resulting in negligible heat loss from the system; and (2) the SOFC is locally isothermal, meaning that the heat transfer process between the gas and SOFC components is not considered.

Based on the above assumptions, the mass and energy conservation of the SOFC can be established via eqs 4 and 5:
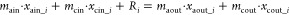
4
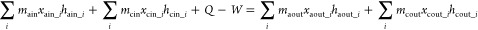
5where *m* is the molar flow (mol·s^–1^); *x* is the molar fraction of species *i*; *h* is the molar enthalpy of species *i* (J·mol^–1^); *R*_*i*_ is the amount of species *i* consumed or produced (mol·s^–1^); *Q* is the heat released by electrochemical or chemical reaction (W); *W* is the electricity generated by electrochemical reactions (W); and the subscripts a, c, in, and out represent the anode, cathode, inlet, and outlet, respectively.

Notably, SOFC configurations include internal and external reforming. Internal reforming indicates that reforming and WGS reactions occur inside the SOFC, whereas external reforming indicates that these reactions occur outside the SOFC through an external reformer. Reforming and WGS reactions must be considered in the internal reforming SOFC model. The reaction rate *R*_*i*_ is typically calculated based on the equilibrium reaction constants,^71,72^ as follows:
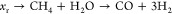
6
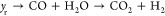
7
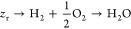
8
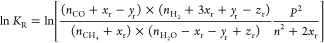
9
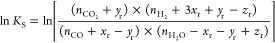
10
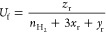
11where *x*_r_, *y*_r_, and *z*_r_ are the molar conversion ratios of the reforming, WGS, and electrochemical reactions, respectively; *K*_R_ and *K*_S_ are the equilibrium constants of the reforming and WGS reactions; *n*_CO_, *n*_CO2_, *n*_H2_, *n*_H2O_, and *n*_CH4_ are the molar flow rates of CO, CO_2_, H_2_, H_2_O, and methane (CH_4_), respectively; and *U*_f_ is the fuel utilization, which is a preset parameter.

The equilibrium constants for the reforming and WGS reactions can be determined by minimizing the Gibbs function, as expressed in eqs 12 and 13:
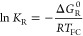
12
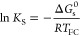
13

By combining eqs 9–13, *x*_r_, *y*_r_, and *z*_r_ can be solved, and the consumption or generation rate *R*_*i*_ of species *i* can be calculated.

The electricity power *W* generated by SOFCs can be calculated using eq 14, as follows:
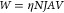
14where *N* represents the number of cells; *J* represents the output current density (A·m^–2^); *A* represents the effective area of a single cell (m^2^); *V* represents the output voltage (V); and η is the DC/AC convert efficiency.

The output voltage *V* can be determined through the relationship between the equilibrium voltage *E*_N_ and polarization voltage *V*_loss_, as expressed by eq 15:
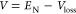
15
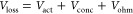
16

The equilibrium voltage *E*_N_ can be calculated using the Nernst equation, as shown in eq 17. The polarization voltage *V*_loss_ can be categorized into three types of polarization: activation (*V*_act_), concentration (*V*_conc_), and ohmic (*V*_ohm_). The three polarization overpotentials (or voltage losses) can be estimated using empirical–theoretical equations, which are summarized in Table 3.
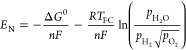
17

**Table 3:** Equations of the Activation, Concentration, and Ohm Polarization Overpotentials^73−75^^a^

polarization overpotentials	model description	remark
Activation loss		*E*_act,a_ and *E*_act,c_ are the activation energies of the anode and cathode, respectively. The values of *E*_act,a_ and *E*_act,c_ are 100 and 120 kJ·mol^–1^, respectively.
		
		
		
		
Ohmic loss^b^		*l*_e_ is the thickness of the electrolyte measured in micrometers.
Concentration loss^c^		*l*_a_ and *l*_c_ are the anode and cathode thickness, respectively. and represent the effective diffusion coefficients of the anode and cathode, respectively. and are the effective Knudsen diffusion coefficients of O_2_. is the tortuosity to porosity ratio of the anode. and are are the molecular diffusion coefficients for H_2_-H_2_O and O_2_-N_2_ binary systems, respectively. *P*_C_ is the operating pressure at the cathode.
		
		
		

a The equation used to estimate the polarization overpotential was originally established based on H_2_-fueled SOFCs but is also applied to SOFCs fueled by syngas or prereforming gas. In these cases, it is assumed that CO does not participate in electrochemical reactions.

b Other equations also describe ohmic polarization, and reliable simulation results have been obtained using these equations. For more detailed information, please see refs (72 and 76).

c If SOFC is fed by hydrocarbon fuel, the  is the oxygen partial pressure after reforming and gas shift reaction.

The heat produced by the electrochemical reactions is calculated as the change in enthalpy of the electrochemical reactions minus the electricity produced, which is expressed by eq 18, as follows:
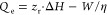
18

**Figure 11:**
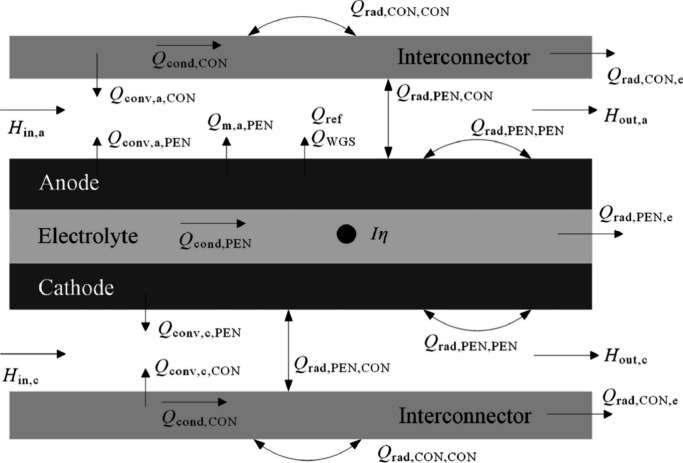
Energy conversion of each component of a planar SOFC along the gas channel. *Q* represents the heat source, and the subscripts conv, cond, and rad represent heat conduction, heat convection, and heat radiation, respectively. The subscripts ref and WGS indicate reforming and WGS reactions, respectively. The subscripts in and out refer to the inlet and outlet, respectively. The subscripts a and c refer to the anode and cathode, respectively. The subscript CON refers to the interconnector. *H* is the gas enthalpy. The subscript *Iη* refers to the irreversible heat generated by electrochemical reactions. Reproduced with permission from ref (10). Copyright 2018 Elsevier.

The above black box model for SOFCs obviously does not consider the physics field information within the SOFC. Moreover, the model utilizes algebraic equations to describe the energy conservation, mass balance, and electrochemical polarization of the SOFC, making the model relatively easy to solve. This model is widely used in system simulations of large SOFC hybrid systems when the research purpose lies in the design and optimization of novel energy systems and estimations of thermodynamic efficiency. Given that incorporating the spatial distributions of SOFC physical parameters will complicate the whole-system model, obtaining accurate results with black box models is difficult. For example, in such models, the SOFC temperature is assumed to be uniform within the entire SOFC stack and heat transfer between the gas and components is not considered. In such cases, the temperature distribution of the SOFC cannot be obtained, and model and experimental results can deviate greatly if the SOFC has severely uneven temperature distribution. To reflect more information within the SOFC, other SOFC system models have been developed for different research purposes. At present, the most widely employed model is the spatial distribution model, which divides the SOFC into five components, namely, the interconnector, anode, cathode, anode channel, and cathode channel. One-dimensional (1D) spatial discretization of the SOFC is carried out along the gas flow direction, and the mass and energy conservation equations for each small-volume element are separately established based on the control volume method. The energy conversion of each component of a planar SOFC along the gas channel is illustrated in Figure 11.

The mass conservation equation of species along the axis coordinate *z* ∈ [0, *L*] in the gas channel region can be obtained using eq 19, as follows:
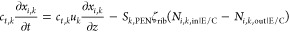
19where *c* represents the concentration of the gas (mol·m^–3^); *x* represents the molar fraction of substance *i*; *u* represents the gas velocity (m·s^–1^); *N* represents the flux of gas in or out of the gas channel (mol·m^–2^·s^–1^); *S* represents the effective mass transfer area (m^2^·m^–3^), which can be found in refs (10 and 70), and ζ represents the rib coefficient. The subscript *k* refers to the anode (a) or cathode (c), and E/C represents the electrode–channel interface.

The energy conservation of the gas in the channel region is given by eq 20, as follows:
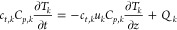
20where *C*_*p*,*k*_ refers to the specific heat capacity of the gas mixture (J·mol^–1^·K^–1^); and *Q*_*k*_ refers to the heat source term (W·m^–3^).

As shown in Figure 11, the heat source term of the anode comprises four parts: (1) convective heat exchange between the fuel and PEN (*Q*_conv,a,PEN_); (2) convective heat exchange between the fuel and interconnector (*Q*_conv,a,CON_); (3) sensible heat flow due to mass transfer (*Q*_m,a,PEN_), referring to the sensible heat flow associated with the temperature difference, which flows into the channel when species enters the channel from the PEN; and (4) heat from the reforming and WGS reactions (*Q*_ref_ and *Q*_WGS_, respectively). The equations for these four heat source terms can be found in refs (10, 70). Notably, if the chemical reactions are thought to occur inside the porous electrode, the chemical reaction heat source terms (*Q*_ref_ and *Q*_WGS_) will not appear in the anode channel. Moreover, the heat source term of the cathode channel does not include the sensible heat flow due to mass transfer (*Q*_m,a,PEN_) and the chemical reaction heat source terms (*Q*_ref_ and *Q*_WGS_).

The energy conservations of the PEN and interconnector are described by eqs 21 and 22, as follows:
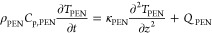
21
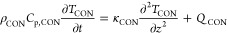
22where ρ refers to the density (kg·m^–3^); κ refers to the heat conductivity (W·m^–1^·K^–1^); *Q* refers to the heat source (W·m^–3^); and the subscripts PEN and CON represent the PEN and interconnector, respectively.

As illustrated in Figure 11, the source terms in the PEN energy conservation equation comprise four parts: (1) the species enthalpy change due to species mass transfer between the channel and PEN (the temperature of species entering the PEN is the gas temperature in the channel, while the temperature of species outflowing from the PEN is the PEN temperature, making the difference between the enthalpies of species flowing in and out of the PEN the sum of the chemical and electrochemical heat); (2) the electrical power generated in the PEN; (3) the convection heat transfer between the PEN and channel (*Q*_conv,a,PEN_ and *Q*_conv,c,PEN_); and (4) the radiation heat flux at the PEN surface (*Q*_rad,PEN,PEN_, *Q*_rad,PEN,CON_, *Q*_rad,PEN,e_). The equations for calculating these heat source terms can be found in refs.^10,70^

To simplify the model, the control equation of gas flow in the channel is not considered; thus, the gas velocity distribution in the channel cannot be obtained. To quantify the gas pressure drop in the flow channel, the pressure drops of the gas in the channels of planar and tubular SOFCs are estimated as follows:^70^
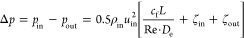
23
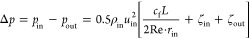
24where *c*_f_ refers to the Colburn friction factor; ζ refers to the friction coefficient; *Re* is the Reynolds number calculated from the inlet gas property; *D*_e_ is the hydraulic diameter for a rectangular flow channel; and *r*_in_ is the inside diameter of a tubular channel. The gas velocity distribution along the flow channel can be roughly obtained by combining the pressure drop along the flow channel and the Bernoulli equation.

Notably, SOFCs are assumed to operate in a quasi-steady state, and the nonsteady-state terms in the governing equations are thus equal to 0. The source terms and parameters in the above equations are determined based on a planar SOFC structure. When modeling other SOFC structures, the source terms or discretization should be adjusted according to the SOFC working process. The source terms and related parameters of tubular SOFCs are given in refs.^10,70^ The temperature distributions of the different components along the gas channel direction can be obtained by coupling the energy and mass conservation equations for the different components and discretizing them along the flow channel direction.

SOFC electrochemical performance can be predicted by the functions of current density and voltage, i.e., *V*_cell_ = *f*(*I*). One of the SOFC operating conditions, such as the power demand *P*, current density *I*, or voltage *V*_cell_, usually needs to be set. Here, *P* is taken as an example to illustrate how other electrochemical parameters can be determined. First, the *P* value should be specified, and then the estimated *I* value should be given. Subsequently, the overpotential *V*_loss_ of each node can be calculated using the equations listed in Table 3 and the voltage of each node can be obtained using eq 15. The average outpower can be obtained, and then the obtained average power can be compared with the specified power. If the error between the average outpower and power load meets the calculation accuracy, the initial *I* value is correct and calculations can be terminated; if not, the estimated *I* value should continue to be changed and the above steps are repeated. The correct voltage distribution along the gas channel direction can be obtained via continuous iteration. Notably, setting the power load or voltage to the known value is recommended because the voltage needs to be solved iteratively if *I* is set to a known value, which will increase the complexity of the solution and increase the difficulty of convergence. The specific iterative computation flows are available in refs (68, 69).

The above-mentioned models represent the two research directions of SOFC system models: (1) the black box model, which is applied to evaluate and optimize steady-state thermodynamic performance; and (2) the parameter distribution model, which applied to detailed analysis of steady-state or dynamic performance. Additionally, some improved SOFC models for specific research objects are reported in the literature, most of which have been developed based on the above two models. The next section reviews the SOFC system models established in the literature, identifying their functions and advantages.

#### 2.2.2 Research Progress of SOFC System Models

##### 2.2.2.1 Zero-Dimensional Models

**Figure 12:**
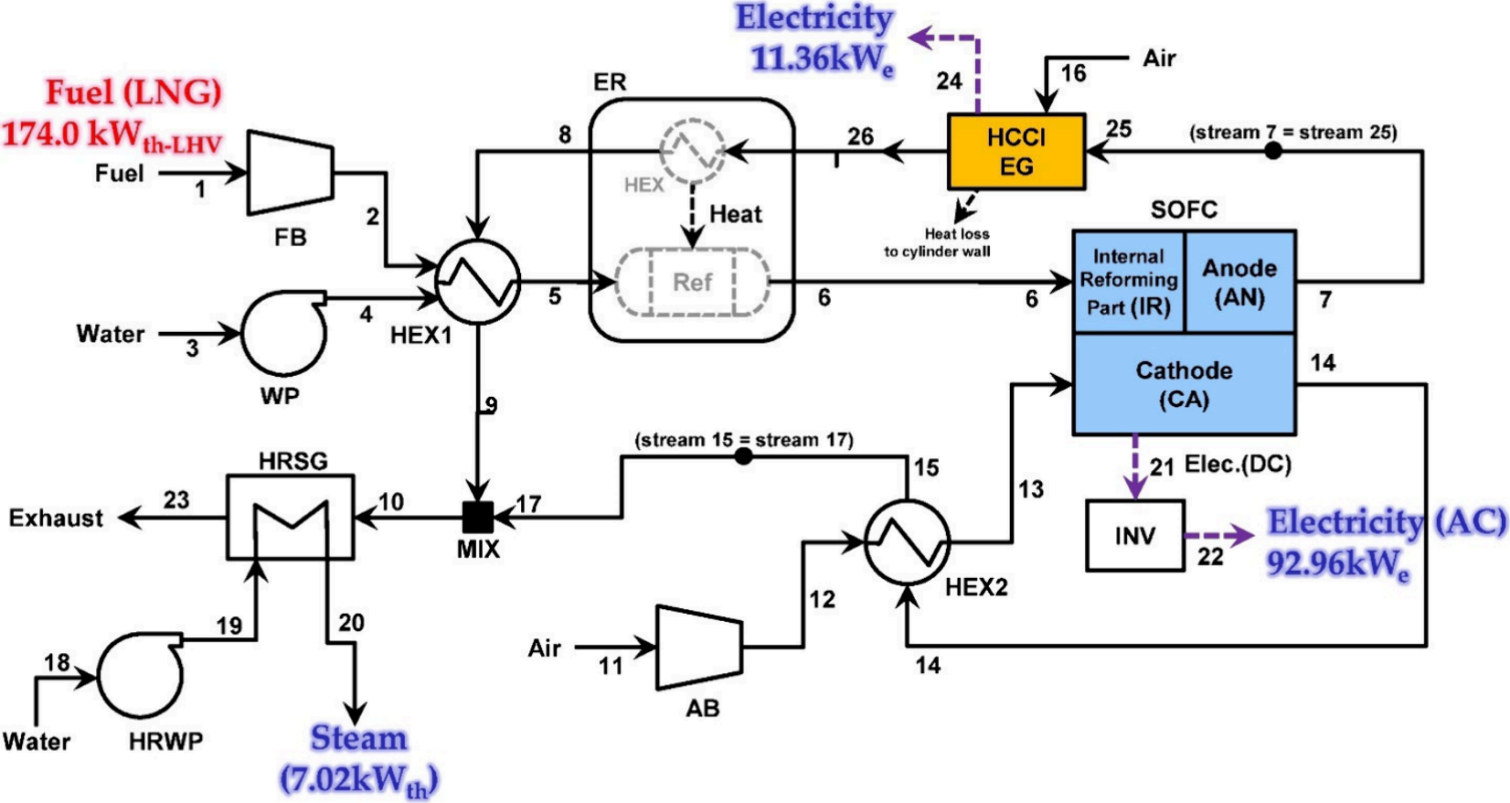
Schematic of the SOFC-Engine hybrid system. Reproduced with permission from ref (47). Copyright 2018 Elsevier.

Black box models, which are generally employed in the thermodynamic design and optimization of large SOFC systems, primarily focus on the composition and thermodynamic state of the inlet and outlet gases but do not involve detailed distributions of the species and temperature within SOFCs. Lee et al.^47^ developed a black box model for thermodynamic simulations of an SOFC-Engine hybrid system. After obtaining the thermodynamic state of each operating point, as shown in Figure 12, the exergy and exergoeconomics of the system were evaluated. Chuahy et al.^77^ proposed the coupling of an SOFC and advanced combustion engine and conducted simulations to determine the electrical efficiency potential of the system. The authors employed a 0D black box model, and the results were validated via experimental electrochemical polarization curves (*I*–*V* curves). Sghaier et al.^77^ developed an integrated energy system combining an internal reforming SOFC, GT, and an absorption refrigeration cycle. A steady-state thermodynamic model was established to investigate the energy and exergy of the system, and the internal reforming SOFC unit was described by a 0D black box model. Koo et al.^55^ modeled a 5 kW SOFC-Engine hybrid power generation system and evaluated the energy and exergy of the system. Although the simulation was performed using Aspen Plus software, the authors did not provide detailed information about the SOFC model. Notably, Aspen Plus is a typical chemical process simulation software in which SOFCs are described by a 0D black box model. Sadeghi et al.^78^ modeled a novel syngas-fueled SOFC power generation system and performed thermodynamic and economic evaluations of the system. A multiobjective optimization method was subsequently applied to balance the cost and efficiency of the system. During the modeling process, the SOFC was described by a 0D black box model and the results were validated experimentally. Shayan et al.^79^ developed a model for an SOFC integrated biomass gasifier system and compared the effects of different agents on system performance from thermodynamic and exergoeconomic perpectives. The performance of the SOFC in this system was described by a black box model. In our previous studies, SOFC-PEMFC^56,80−82^ and SOFC-Engine^53,54,83,84^ hybrid energy conversion systems were proposed, and black box models were employed to model the SOFCs and other components involved in the systems. System performance was evaluated from energy, exergy, exergoeconomics, and environmental perspectives,^54^ and the efficiency and cost of the systems were balanced using multiobjective optimization theory.^83^ These efforts confirmed the benefits of advanced SOFC-based energy conversion systems in terms of high efficiency, low cost, and clean conversion.

**Figure 13:**
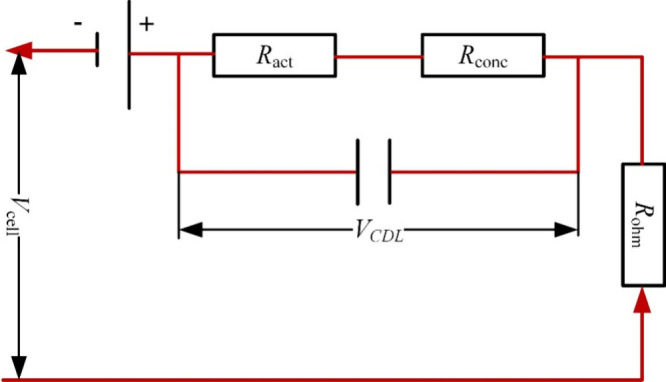
Schematic of the first-order RC circuit. Redrawn and modified with permission from ref (85). Copyright 2019 Elsevier.

Based on black box steady-state models, we previously proposed dynamic models for SOFC hybrid systems.^85,86^ The dynamic response of SOFCs is mainly influenced by the electrochemical reactions, fuel supply, and temperature change. Among them, the dynamic response induced by electrochemical reactions, as an inherent feature, is important for the dynamic behavior of SOFCs. Therefore, the dynamic response characteristics of SOFCs can be obtained by identifying and modeling these three dynamic processes. The charge double-layer phenomenon between the electrode and electrolyte is generally thought to behave like a capacitor. Thus, the electrochemical dynamic response can be explained by the principle of a capacitor.^85^ During the SOFC discharge process, charge accumulates in the capacitor plates, generating voltage. The continuous accumulation of charge leads to the generation of voltage between the plates, which is actually the sum of the activation and concentration overpotentials, as expressed by eq 25. Since charge accumulation takes time, the activation and concentration overpotentials cannot immediately respond to sudden changes in the current density. By contrast, the ohmic overpotential can respond quickly to such changes. Therefore, the dynamic behavior of the SOFC can be modeled as a first-order RC circuit, as illustrated in Figure 13. Therein, the variation in *V*_CDL_ with time *t* can be described by eq 26, and the relationship between *V*_CDL_ and *t* can be obtained by solving the ordinary differential, as expressed by eq 27. Variations in the fuel flow rate cause changes in the gas partial pressure, which affects the output voltage of the SOFC. This is reflected in the calculation of the Nernst voltage and overpotential. The molar flow rate of the gas through the valve is assumed to be proportional to its partial pressure, as expressed by eq 28. Additionally, the change in gas molar flow rate is considered to be a first-order process. Thus, the dynamic response characteristics of different gas partial pressures can be described by eqs 29–31, and the responses of different gas partial pressures with time can be obtained by the inverse Laplace transform of eqs 32–34. The fuel flowrate change process was described by step function.
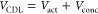
25
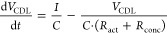
26
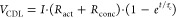
27where τ_c_ is the time constant related to the transient road; *C* is the equivalent capacitance of the double charge layer; *V*_CDL_ is the voltage of charge double layer; *I* is current; *R*_act_ and *R*_conc_ are activation resistance and concentration resistance, respectively.
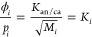
28where *K*_*i*_ is the proportional constant.
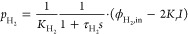
29
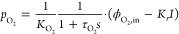
30
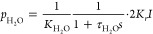
31where *K*_H_2__, *K*_O_2__, and *K*_H_2_O_ are the valve molar constants for H_2_, O_2_ and H_2_O, respectively; *K*_r_ is the constant, which is N/4F; τ_H_2__, τ_O_2__ and τ_H_2_O_ are the response time constants for H_2_, O_2_ and H_2_O, respectively.
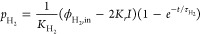
32
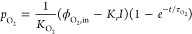
33
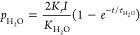
34

The dynamic response characteristics of the whole system can be obtained by coupling the SOFC dynamic model with the dynamic processes of other components. We previously reported the dynamic characteristics of SOFC-WGS-TSA-PEMFC and SOFC-Engine hybrid energy systems.^85,86^ The results indicated that the SOFC voltage needed at least 1 s to restabilize, while the PEMFC voltage needed no more than 0.1 s to restabilize after a load step change. SOFCs exhibit a poor dynamic response due to their high operating temperature. Additionally, SOFCs have slower response characteristics than engines. The dynamic response process of the SOFC is an inertia process, while engine realization is a delay process. The SOFC needed 1.31 s to reach 63.2% maximum power, with a time constant of 1.31 s. By contrast, the delay time for the engine to reach maximum power was 0.2 s. In short, the dynamic response of the SOFC is relatively slow due to its high operating temperature, which dominates the short-term dynamic response of the whole system. Notably, the above SOFC dynamic model only considers the dynamic characteristics of the electrochemical and fuel supply processes and does not consider the slower temperature change process during start-up or state switching. This is also determined by the model’s features as the black box model does not consider the phenomena of heat and mass transfer. However, the model conveniently describes the dynamic response process of the overall system performance with little temperature change. In the next section, we focus on the SOFC dynamic response through more complex and accurate dynamic multiphysics models (MPMs).

##### 2.2.2.2 Spatial Distribution Models

**Figure 14:**
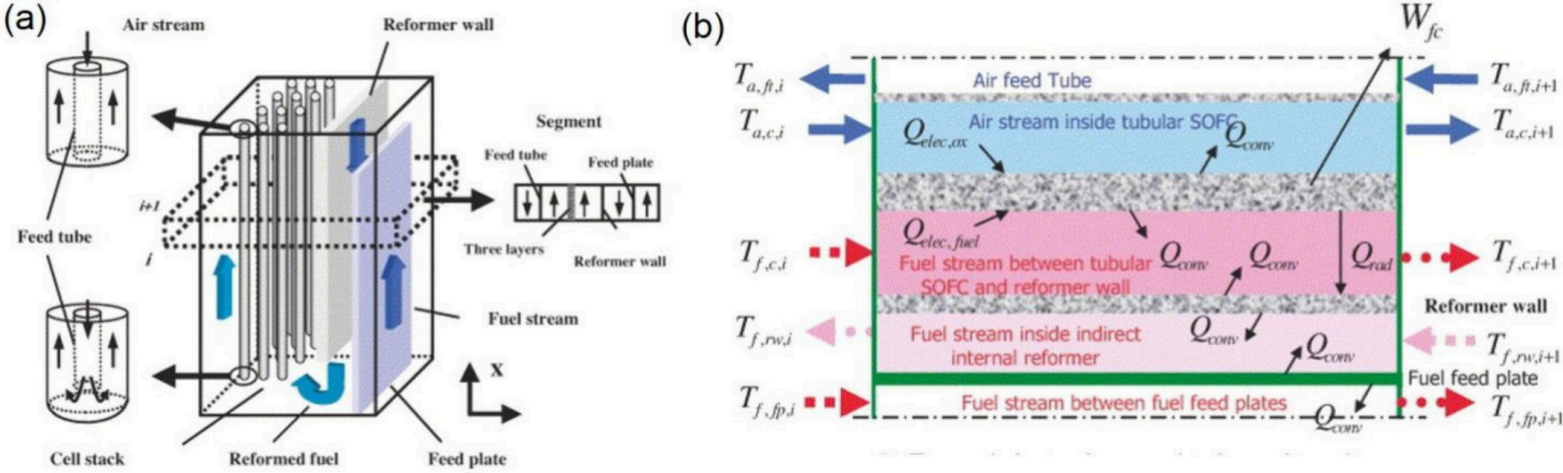
Schematic of (a) SOFC segments along the longitudinal direction and (b) the energy conservation equation with control volumes. Reproduced with permission from ref (67). Copyright 2005 Elsevier.

In addition to black box models, physics-oriented models have been developed to provide more detailed information within the SOFC for system simulations. Many models have been reported in the literature, each with a unique focus, making them challenging to present under a unified framework. However, a common feature of these models is the discretization of the SOFC structure along the flow channel to solve the spatial distributions of the internal parameters. For example, Song et al.^67^ developed a quasi-two-dimensional (2D) tubular SOFC model that considered the heat and mass transfer characteristics, which they employed to evaluated the performance of an SOFC/MGT hybrid power generation system. The model divided the cell and reforming chamber into multiple layers along the longitudinal direction, as illustrated in Figure 14(a). Each segment consisted of several control volumes separated by different walls. Within each segment, the energy conservation and mass transfer phenomena were described using the control volume method, as demonstrated in Figure 14(b). This quasi-2D model could predict the longitudinal temperature field, species concentrations, and current density, making it useful for simulating SOFC-GT systems to analyze their performance.

Stiller et al.^66^ developed a 2D steady-state finite volume model of an SOFC fueled by CH_4_, which was applied to simulate an SOFC-GT system and assess its performance. The authors conducted a detailed analysis of the ohmic resistance and heat transfer processes, including heat conduction, convection, and radiation, which was based on the given basic geometric structure and general simplifications. Additionally, the authors described in detail the effects of the chemical reactions on the mass conservation and energy balance. The final discrete equations derived from the finite volume model were solved using Fortran 90. When comparing the simulation results of the SOFC with other models reported in the literature, the authors found that the maximum deviation of the output voltage between the planar SOFC model and a previously reported model was 2.64%; that between the voltage of the atmospheric tubular SOFC model and the reported experimental results was 0.72%; and that between the pressurized tubular SOFC model and the reported experimental results reached up to 13.3%. The authors attributed these deviation errors to incomplete input parameters and experimental results. In summary, the study analyzed the energy and mass conservations of planar and tubular SOFC models using the finite volume method (FVM) and considered the influence of thermal radiation on heat transfer, a factor often overlooked in most models. The case study analysis confirmed that the model error was within the acceptable range. This study provides a fundamental model for subsequent SOFC-GT system modeling.

**Figure 15:**
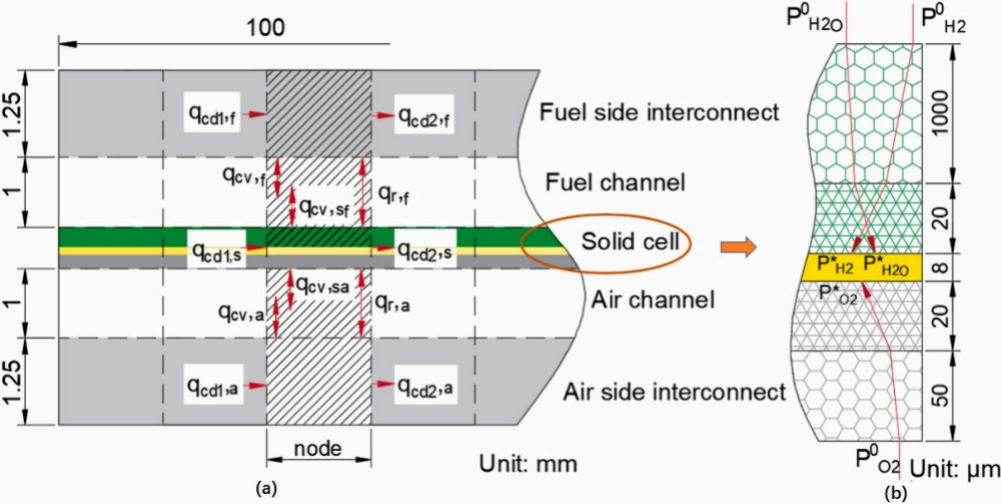
Schematic of SOFC discretization and heat transfer processes at a node. The subscripts cd, cv, and r refer to heat conduction, convection, and radiation, respectively. The subscripts f, a, s, 1, and 2 represent the fuel channel, air channel, solid cell, inlet, and outlet, respectively. Reproduced with permission from ref (69). Copyright 2024 Elsevier.

Ma et al.^69^ constructed a 2D steady-state finite volume model of an SOFC, which was integrated into an SOFC system model. The main feature of the SOFC system configuration was that burner temperature stabilization was maintained by controlling the cooling air bypass flow to the burner, which weakened the thermal shock to the SOFC stack. This system model was mainly utilized to analyze and quantify the effects of the fuel utilization rate, air flow rate, and maximum burner temperature on system performance. In this 2D finite volume model, the SOFC was discretized into 5 × *N* volume nodes, where *N* indicates the number of nodes along the cell length and 5 represents five components, namely, the fuel channel, air channel, PEN, anode interconnector, and cathode interconnector. Each N node was associated with five components in the thickness direction. The specific discrete method is illustrated in Figure 15. Temperature changes were considered in the length and thickness directions, while gas diffusion was only considered in the flow channel direction. Three heat exchange methods must be considered for different SOFC components: heat conduction, convection, and radiation. The SOFC system model was obtained by combining the SOFC model with black box models of other auxiliary components. The modeling results indicated that the cooling air bypass flow could minimize the thermal shock of the stack, and the efficiency of the system could reach >55%. This SOFC model was basically the same as the parameter distribution models described in Section 2.2.1. Parameter distribution models can be used to obtain the stack temperature distribution while ensuring that the system model is not too complicated to solve.

The above three cases are faithful practitioners of the SOFC parameter distribution models described in Section 2.2.1, with slight differences. For example, the above models do not consider the gas velocity distribution caused by the gas pressure drop. In addition, the model reported by Ma et al. did not consider the heat radiation of the PEN and interconnector to the environment and the interconnector–interconnector and PEN–PEN heat radiation. Therefore, the SOFC models presented in Section 2.2.1 can be considered relatively comprehensive models. The models in previous studies were slightly modified based on the model framework presented in Section 2.2.1. Next, we introduce two different cases, which are simplified or expanded based on the previous 2D parameter distribution models.

Botta et al.^65^ developed a 1D dynamic model by discretizing the SOFC in the flow channel direction. Since this study focused on controlling the local temperature gradient and fuel utilization, the temperature was only discretized in 1D along the flow direction and the distribution of species concentration was not considered. The equation reported for solving the SOFC temperature is presented in eq eq 35, where the right side of the equation represents the convection heat transfers between the air and PEN connector and between the fuel gas and PEN connector, as well as the power production of the SOFC itself. The power consists of the sum of the enthalpy flow and electric power.
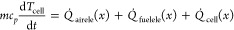
35
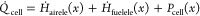
36

Controlling the temperature gradient and fuel utilization is crucial during transient operations because fuel starvation and an excessive temperature gradient can lead to thermomechanical stress, which may cause component fracture or degradation. The authors controlled the variation in air and fuel flows using a proportional-integral (PI) controller to maintain the fuel utilization rate and temperature gradient within a reasonable safety range. Given that the authors only needed to control the local temperature gradient and fuel utilization, they established a 1D equation for temperature and defined the fuel utilization parameter. Hence, the model was greatly simplified and the simulation efficiency was improved based on the premise of realizing the purpose of the study.

**Figure 16:**
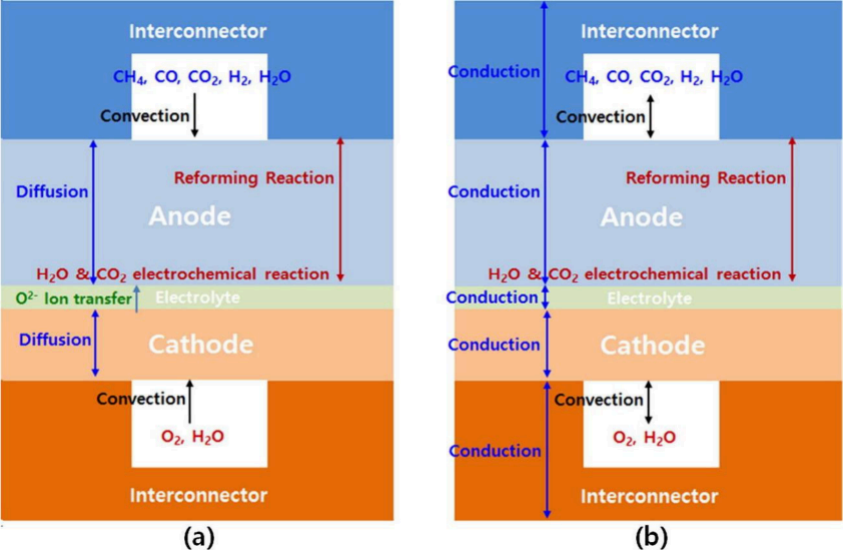
Schematic of volume discretization for solving (a) species conservation and (b) energy conservation. Reproduced with permission from ref (68). Copyright 2017 Elsevier.

Kang et al.^68^ developed a quasi-3D model of a planar SOFC to describe the dynamic performance of an SOFC-Engine hybrid system. Their study mainly focused on the responses of important parameters and spatial distributions of related parameters in the SOFC-Engine hybrid system with changing power demand of the SOFC. The model considered heat transfers in the solid interconnector, gas channel, and the PEN, as well as mass transfer of the gas flowing in the channels. The mass conservation equation was solved by discretizing the SOFC into five control volumes along the cross-sectional direction, namely, the anode channel, anode, electrolyte, cathode, and cathode channel. Similarly, the SOFC was discretized into seven control volumes along the cross-sectional direction to solve the energy conservation equation, including the interconnector, anode channel, anode, electrolyte, cathode, cathode channel, and interconnector, as illustrated in Figure 16. Heat radiation was not considered in the energy conservation equation, as it would lead to an overestimation of the temperature gradient. In addition, the research object was a crossflow SOFC stack; thus, the energy and mass conservation equations were discretized in 3D. Overall, this model was one of the most complex parameter distribution models and was closer to an MPM, which will be discussed in the next section. Compared with the spatial distribution model introduced in Section 2.2.1, the model framework was the same, that is, the equations governing species concentration and temperature were established and then solved. However, this model discretized the governing equations directly in 3D space, which greatly increased the difficulty of finding a solution.

In general, SOFC spatial distribution models employed in system simulations can reflect the spatial distributions of specific parameters and provide more detailed information than black box models. However, the establishment and solving of such models are more complicated. Compared with cell/stack MPMs, spatial distribution models are insufficient for the coupling of multiphysics fields. Therefore, spatial distribution models can be seen as a compromise between black box models and MPMs. Additionally, most spatial distribution models are solved using in-house programs, which can be easily coupled with other system component models. Self-programmed models are commonly utilized, especially when commercial software is not well developed.

Much SOFC system modeling work has been performed. Table 4 summarizes some representative work and elaborates the details that deserve attention.

**Table 4:** Summary of Representative SOFC System Models^a^

authors	dimension	model state	cell type	model type	solution method	model validation	system configuration	system performance	main concerns	ref
Song et al.	0D	Steady	Stack	Black box	MATLAB	-	SOFC-RC-KC	Exergy efficiency = 39.43%	Establishment of thermodynamic model of the system and operating conditions and heat transfer structure optimization.	(87)
Huerta et al.	0D	Steady	Stack	Black box	ThermoLib	Experimental	Diesel prereforming SOFC	Exergy efficiency = 53% and Energy efficiency = 56%	The influence of CO oxidation on OCV and voltage polarization was considered. Heat loss was also considered. The temperature change of the interconnector and PEN was not considered.	(88)
Huang et al.	1D	Steady	Planar Stack	FVM	MATLAB	Experimental	NG-fed SOFC-GT	Energy efficiency = 74%	The temperature distribution of the cell was calculated by 1D spatial discretization along the flow channel direction.	(50)
Wang et al.	3D	Steady	Planar Stack	MPM	DETCHEM	Experimental	Biogas-fed SOFC-CHP	Energy efficiency = 84.8% and electrical efficiency = 55.4%	DETCHEM software was used to build the SOFC MPM, and gPROMS was used to build the system model. Data transfer between the system and SOFC model was implemented through MATLAB.	(89)
Wang et al.	0D	Steady	Stack	Black box	Aspen Plus	Experimental	Diesel CLGH-SOFC-AR CCHP	Electrical efficiency = 54.1% and exergy efficiency = 52.3%	The thermodynamic equilibrium model of the SOFC was established, and the thermodynamic performance of the system was analyzed and optimized.	(90)
Alirahmi et al.	0D	Steady	Stack	Black box	EES	Model	SOFC-ORC-CAES	Exergy efficiency 45.7%	The thermodynamic equilibrium model of the SOFC and its system was established. The ANN was used to identify the system model, and the gray wolf optimization algorithm was used to optimize the system.	(91)
Meng et al.	0D	Steady	Stack	Black box	Aspen Plus	Model	MethanolSOFC-PEMFC	Maximum electrical efficiency = 75%	The polarization voltage calculation method was different from that in Table3. The polarization model was in good agreement with the experimental data.	(57)
Cardenas et al.	0D	Steady	Stack	Black box	**-**	**-**	Pressurized SOFC-GT	Electrical efficiency = 66% and energy efficiency = 84.8%	The polarization voltage of SOFC was calculated by constant area specific resistance (ASR), and the SOFC model was not validated.	(92)
Emadi et al.	0D	Steady	Stack	Black box	MATLAB	Experimental	SOFC-dual ORC	Exergy efficiency = 51.6%	The thermodynamic equilibrium model of the SOFC and its system was established, and the system performance was optimized by multiobjective optimization theory.	(61)
Van et al.	1D	Steady	Planar Stack	FVM	MATLAB	Experimental	Reforming SOFC	Maximum stack efficiency = 65.6% and maximum system efficiency = 61.4%	The mass and energy conservation were discretized along the flow channel. The SOFC polarization voltage was calculated using the equations in Table3. The system model was developed in the Cycle-Tempo software.	(93)
Van et al.	1D	Transient	Planar Stack	FVM	MATLAB	Experimental	Reforming SOFC	-	The model was similar to that above, but this model studied the dynamic behavior.	(94)
Rokni et al.	0D	Steady	Stack	Black box	Dynamic Network Analysis	Experimental	Reforming SOFC	Maximum electrical efficiency = 55%	The thermodynamic equilibrium model of the SOFC was established under different fuel reforming conditions. The polarization voltage calculation was different from the equations in Table3. The pressure was also considered as 1 × 10^–3^ bar.	(95)
Min et al.	1D	Steady	Planar Stack	FVM	C#	Experimental	Reforming SOFC	Efficiency fluctuates greatly with parameter change	The mass and energy balance were discretized along the gas channel. The temperature changes in the interconnector and PEN were not considered. The fuel and air temperatures were almost the same along the cell. The pressure was given as an experimental value.	(96)
Shi et al.	0D	Transient	Planar Stack	Black box	Aspen Plus	Experimental	Micro SOFC-CHP	Maximum electrical efficiency = 44.9%	The polarization voltages were described by the equations in Table 3. The heat loss of SOFC was 1% HHV (High Heat Value) of inlet fuel. The relationship between voltage degradation rate and fuel utilization and current density was proposed.	(97)
Botta et al.	1D	Transient	Planar Stack	FVM	Modelica	Model	SOFC stack	-	A 1D transient SOFC model was established, and PI control was used to maintain the fuel utilization and maximum temperature gradient in a safe range.	(65)
Ma et al.	2D	Steady	Planar Stack	FVM	MATLAB	Experimental/Model	SOFC stack/SOFC system	Maximum electrical efficiency = 55%	A 2D steady-state finite volume model of the SOFC was developed and integrated into the SOFC system model.	(69)

a RC, Rankine cycle; KC, Kalina cycle; NG, natural gas; CHP, combined heating and power; CLGH, chemical looping H_2_ generation; AR, absorption refrigeration; CCHP, combined cooling heating and power; CAES, compressed air energy storage; ORC, organic Rankine cycle.

### 2.3 Comparison of Black Box and Spatial Distribution Models

The methods for simulating SOFCs at the system scale mainly include black box and spatial distribution models. Black box models are primarily utilized in system-scale thermodynamic simulations, facilitating evaluations of fundamental operating thermodynamic data to comprehensively explore the potential of the system for further development. To improve energy conversion performance, more components and thermal cycles can be included in the system. Although the simplicity of black box models makes them convenient for modeling large thermodynamic systems, they do not account for the internal working mechanism of SOFCs, and their validation mainly involves examining apparent *I*–*V* curves, as demonstrated in refs (72, 82, 98). Additionally, owing to the conceptual stage of designed SOFC systems and lack of corresponding experimental platforms, further validating such SOFC models is challenging.

Black box models have demonstrated the great potential of SOFC hybrid systems to generate clean power. Consequently, establishing spatial distribution models is necessary to further investigate the internal working mechanism and obtain important parameter distributions within SOFCs. Compared with black box models, parameter distribution models can provide more detailed information with more accurate simulation results. Parameter distribution models are mainly applied to capture important parameter distributions within the cell, such as temperature, species concentration, etc., so that steady-state electricity production performance can be optimized at the system scale, operating conditions can be identified to achieve long-term stable operation, and a dynamic model or control strategy can be developed. Given that SOFC systems involve several components, adopting spatial distribution models to describe system performance can achieve a balance between model complexity and information. With research progress, experimental setups have gradually been established under the guidance of numerical models, and their reliability can be confirmed experimentally. For instance, Lee et al.^47,76,99^ developed a black box model of an SOFC-Engine system, conducted a comprehensive evaluation of the system’s performance, and confirmed its development potential. Subsequently, the authors established a 3D spatial distribution model of the SOFC to explore the performance of the entire system in detail.^68^ Finally, the authors successfully constructed an experimental platform to validate the model and complete development of the entire system.^55,100^ This approach represented a comprehensive, effective project or technology development practice in which different model types played distinct roles.

In short, black box and spatial distribution models have unique focuses and are applicable in different scenarios. Table 5 summarizes the characteristics of the two models to enable selection of the appropriate model based on the research objective and purpose. Notably, spatial distribution models are actually upgraded black box models. Therefore, the function achieved by a black box model can be entirely realized by a spatial distribution model. However, the process of establishing a spatial distribution model is more complicated than a black box model and such models are difficult to solve. Therefore, the appropriate model should be selected according to the problem to be studied. For example, a black box model is more appropriate than a spatial distribution model if researchers want to design a novel SOFC integrated system and explore its performance potential (efficiency, cost, etc.). Although black box models consider the influences of temperature and gas concentration when calculating the SOFC output voltage, the physical structure and other parameters of the SOFC are not considered. The *I–V* curve of the SOFC can be verified and its typical performance can be obtained as long as the temperature and gas concentration are determined. This is sufficient to explore the performance of novel SOFC system configurations. Black box models are not intended to provide a detailed analysis of the performance of a particular SOFC but simply demonstrate its general performance. At present, most studies focus on *I*–*V* curves for model verification. Nevertheless, the outlet gas composition and temperature should be verified to ensure model reliability.

**Table 5:** Characteristics of Black Box and Spatial Distribution Models

	model type	
criteria	black box	spatial distribution
Information provided	*I*–*V* curve, outlet gas concentration, and temperature	*I*–*V* curve, outlet gas concentration, temperature, spatial distributions of specific parameters
Assumptions	• SOFC temperature distribution is uniform.	• The temperature or species concentration is uniformly distributed in nondiscrete directions.
	• The distributions of species concentrations within the SOFC are not considered.	
Parameters	• Inlet gas composition	• Inlet gas composition
	• Inlet gas flow rate	• Inlet gas flow rate
	• SOFC operating temperature	• SOFC operating temperature
		• SOFC geometry
		• Electrode/electrolyte material
Model application condition	• The temperature difference inside the SOFC is not very large.	• The temperature and species concentrations do not change dramatically in the nondiscrete direction.
	• The species concentrations inside SOFC do not drastically change.	
Construction method	Lumped parameter	Control volume
Construction difficulty	Easy	Difficult
Solution difficulty	Easy	Difficult
Information	Deficient	Abundant
Model function	• System configuration design	• System configuration design
	• Performance evaluation and optimization	• Performance evaluation and optimization
		• Parameter distribution capture
Applied target	• System performance evaluation and optimization	• System performance evaluation and optimization
	• Application potential exploration	• Stability operation strategy development
		• Study on dynamic characteristics or control strategies
Model verification	• Comparison of *I*–*V* curves	• Comparison of *I*–*V* curves
	• Comparison of the temperature and composition of the outlet gas	• Comparison of spatial distributions of important parameters

### 2.4 Challenges and Prospects of System Models

Black box and spatial distribution models have been well developed in terms of model construction and solving. However, these models have some reliability and accuracy issues. Most studies acknowledged that the pressure drop was approximately 5%, which could be ignored, and the equipment was well insulated, with no heat dissipation. Although such assumptions are reasonable and can be utilized to simplify SOFC models, the specific quantified effects of these assumptions on system performance have not been determined. Additionally, the calculation method of outlet gas temperature in black box model needs further discussion. Currently, there are two methods for calculating the outlet gas temperature. One is to ignore the temperature rise of the PEN and interconnector, and all the heat released by the reactions is absorbed by the gas. The other is to assume a definite gas temperature rise, so that the temperature of the outlet gas can be directly determined. The former method overestimates the outlet gas temperature by ignoring the heat capacities of the interconnector and PEN. The latter method requires an estimate of the inlet and outlet gas temperature rise based on the experimental data. The spatial distribution model is more detailed, which calculates the PEN and interconnector temperatures along the length of the cell. Black box models are only validated via *I*–*V* curves, neglecting validation of the temperature and outlet gas composition, which should be improved. Regarding spatial distribution models, important parameter distributions within the SOFC are not well validated in existing models. Such studies typically employ *I*–*V* curves due to their ease of measurement, although temperature distribution measurements can be achieved and more experimental measures are required. By contrast, species concentration measurement is somewhat challenging. Consequently, few simulation studies have compared important parameters such as temperature and gas concentration beyond *I*–*V* curves. Nonetheless, system models retain credibility, as both species concentration and temperature can be calculated from the principles of mass and energy conservation. Moreover, further research on dynamic simulations of SOFC systems is warranted, especially the establishment of nonsteady-state models for start-up or shutdown states or changing working conditions. In summary, subsequent development of SOFC system models should focus on the following aspects:

- Further discussion and improvement are needed for the two current calculation methods of outlet gas temperature. For example, the heat absorbed by PEN and interconnector should be reasonably considered for the first calculation method. For the second calculation method, it is necessary to estimate the inlet gas temperature rise reasonably.
- Making the black box model a meaningful subroutine owing to its inherently stable and consistent framework. It is easily coupled with other system component models to evaluate the performance of advanced SOFC systems.
- Developing transient spatial distribution models for SOFCs, including start-up/shutdown or variable operating conditions, for dynamic simulations of SOFC systems.
- Leveraging system simulation results to develop demonstration systems. Following this, the demonstration system performance can be tested, enabling a comparative analysis with numerical simulation outcomes to assess or refine the model.

## 3 Development of Cell/Stack Models

### 3.1 Overview of SOFC Cell/Stack Models

SOFC system models can be easily coupled with other components to simulate the working state of the whole system. However, system-scale models do not involve the inherent multiphysics coupling mechanism of SOFCs, especially in black box models. Therefore, conducting in-depth investigations into the efficient and stable working mechanism of SOFCs is challenging at the system level. Cell/stack models solve this problem, ensuring efficient and stable operation of SOFC systems. In return, system simulations provide the necessary boundaries or operating conditions for cell/stack models. In fact, cell/stack models are a detailed extension of the spatial distribution models described in Section 2.1. Cell/stack models focus on the full physics field within the whole geometry, including mass/momentum/charge transfers and energy conservation. These processes are coupled, making cell/stack models typical MPMs. While system-scale spatial distribution models also consider spatial distribution to some extent, they mainly focus on temperature and species concentration without considering charge and momentum transfer processes as extensively as MPMs. Thus, a spatial distribution model can be seen as a simplification of an MPM in terms of complexity and accuracy. MPMs accurately describe the physical phenomena within the cell but are more complex and time-consuming to solve than black box and spatial distribution models.

**Figure 17:**
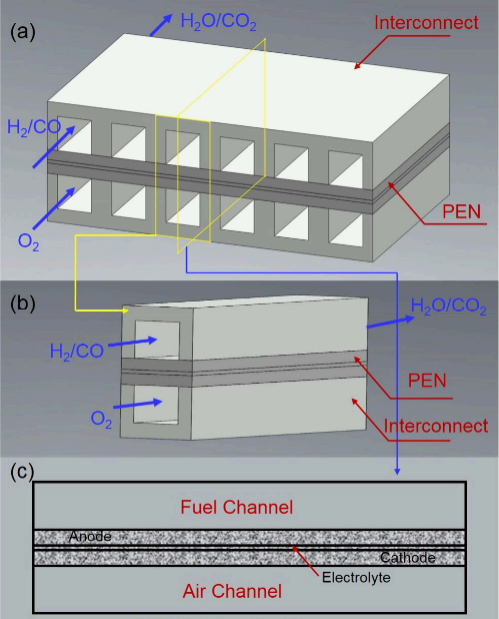
Schematic of (a) 3D planar SOFC unit, (b) 3D single-channel SOFC geometry along the main flow direction, and (c) 2D single-channel SOFC geometry along the main flow direction.

Figure 17(a) presents the 3D structure of a coflow planar SOFC unit, consisting mainly of the interconnector and PEN. Figure 17(b) illustrates the 3D single-channel cell structure derived from an SOFC unit using the periodicity principle. Figure 17(c) depicts the 2D single-channel cell geometry without an interconnector. Notably, only a planar SOFC unit with coflow or counterflow can be simplified to a single-channel cell, whereas the crossflow structure cannot be simplified. Generally, the 3D single-channel geometry illustrated in Figure 17(b), which accurately represents the working process of the SOFC unit, is primarily employed in modeling to reduce the computational load. According to the periodicity principle, the outpower of the SOFC stack is equivalent to that of the single-channel cell multiplied by the number of cells. To further reduce the computational load, the geometry can be simplified to a 2D single-channel structure when the influence of the interconnector is ignored, as illustrated in Figure 17(c) when the influence of the interconnect is ignored. However, the 2D single-channel structure cannot perfectly reflect SOFC performance without considering the interconnector. Nevertheless, adopting a 2D single-channel structure significantly reduces the computational load when dealing with complex transport processes. Despite differing geometry, the physical principles governing SOFC operation remain the same. Fuel gas enters the SOFC through the anode flow channel formed by the interconnector and diffuses to the anode to undergo electrochemical oxidation reactions, while air or O_2_ enters the SOFC through the cathode channel and diffuses to the cathode to undergo electrochemical reduction reactions. O^2–^ produced at the cathode migrates through the dense electrolyte to the electrolyte–anode interface, combines with the anodic fuel gas to produce H_2_O or CO_2_, and generates electricity. Therefore, the fundamental working process of SOFCs involves momentum/mass/charge transfers, energy conservation, and the physical processes of electrochemical reactions. Moreover, fuel reforming is involved if the SOFC is fed by fuel other than H_2_ or CO.

Generally, SOFC MPMs abide by the following assumptions:

- The distribution of electrochemical/chemical reaction active sites is uniform within the porous electrode, meaning a homogeneous model is developed;
- The electronic and ionic conducting phases are uniformly distributed inside the porous electrode;
- Only the electrochemical reactions of H_2_ and CO are considered owing to their fast electrochemical reaction rates;
- Factors influencing SOFC durability, such as carbon deposition and Ni coarsening, are not considered in steady-state models but are included in transient performance degradation models;
- The gas flow in the channel is laminar;
- The gas fed into the SOFC obeys the ideal gas equation of state.

#### 3.1.1 Governing Equations of Mass Transfer

The gas transfer process in the electrode can be divided into gas transfer in the flow channel or in the porous electrode. The mass transfer process of gas is typically described by the concentrated species transport model. The dusty gas model (DGM) is commonly employed to describe the gas mass transfer process within the porous electrode. The mass transport processes in the flow channel and porous electrode can be described by eqs 37 and 38, as follows:
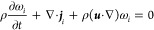
37
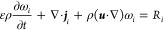
38
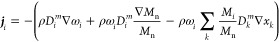
39where ρ is the density of gas mixture (kg·m^–3^); *ω*_*i*_ is the mass fraction of species *i*; ***j***_*i*_ is the mass diffusion flux of species *i* (kg·m^–2^·s^–1^); ***u*** is the velocity of the gas mixture (m·s^–1^); ε is the porosity of electrode; *R*_*i*_ is the rate of generation or consumption of species *i* (kg·m^–3^·s^–1^); *M*_*i*_ is the molar mass of species *i* (kg·m^–3^); *x*_*k*_ is molar fraction of species *k*; *D*_*i*_^*m*^ is mean diffusion coefficient of species *i* (m^2^·s^–1^), which can be calculated by eq 40; and *M*_*n*_ is mean molar mass (kg·m^–3^), which is calculated by eq 41.
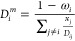
40
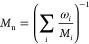
41where *D*_*ij*_ is the binary diffusion coefficient of species *i* and *j*, which can be calculated using eqs 42–44. The mass transfer coefficient of the gas in the porous medium is corrected as the effective mass transfer coefficient *D*_*ij*_^eff^, which is expressed by eq 45, as follows:
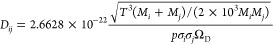
42
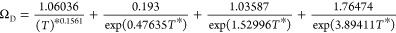
43
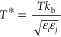
44
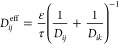
45
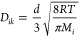
46where *T* is the operating temperature (K); *p* is the operating pressure (Pa); *σ*_*i*_ and *σ*_*j*_ are the potential characteristic length of species *i* and *j*, respectively (m); Ω_D_ is a dimensionless diffusion collision term; *k*_b_ is Boltzmann constant (J·K^–1^); *ε*_*i*_/*k*_b_ and *ε*_*j*_/*k*_b_ are the minimum potential energies of species *i* and *j* (K), respectively; ε is the electrode porosity; τ is electrode tortuosity; and *d* is pore diameter of porous electrode (m). The potential characteristic length of gas molecular σ and the minimum potential energy ε/*k*_b_ can be obtained by searching the gas physical properties database.^101^

It is worth noting that the Fick (FM) and Stefan-Maxwell (SMM) models can also describe the mass transfer process in porous electrodes. The mass diffusion flux of FM and SMM are shown in eq 47 and eq 48.
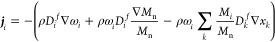
47
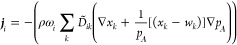
48where *D*^*f*^ is the diffusion coefficient of species *i*; *D*_*ik*_ is Binary diffusion coefficient between species *i* and *k*; *p*_A_ is the partial pressure.

FM is the simplest multicomponent gas transport model and considers gas diffusion and convection.^102^ SMM precisely takes into account binary diffusion and ignores Knudsen diffusion.^103^ DGM is strictly derived based on theory, accurately describing the diffusion behavior of a gas in a porous medium. These three mass transport models are described in detail by Andersson et al.^103^ and Bao et al.,^10^ including their expressions and application scope. The reliability and accuracy of these models have also been compared. Suwanwarangkul et al.^104^ modeled the mass transfer process inside the porous anode to predict the concentration overpotential based on the mass transfer mechanisms of FM, SMM, and DGM. The authors validated the models using H_2_–O_2_–Air and CO–CO_2_ systems and explored the influence of pore size on model prediction. The comparison results indicated that, among the three models, DGM could most accurately describe the multicomponent gas transport process in the porous electrode. Based on the flux ratio method and the fact that the pressure gradient was ignored by Suwanwarangkul et al.,^104^ Bao et al.^105^ compared the three mass transfer models for determining the anode concentration overpotential. The authors concluded that DGM was the best choice for describing the multicomponent gas transfer process in SOFC modeling. In summary, although DGM is the most complex among the three models, it is also the most reliable and accurate method for modeling multicomponent gas transfer in a porous electrode. Consequently, DGM has been widely adopted with the rapid development of computing^105−111^ and is described in detail here.

#### 3.1.2 Governing Equations of Momentum Transfer

The momentum transfer model includes the gas channel and flow in the porous electrode. Momentum transfer in the flow channel is described by the classical N–S equation, as shown in eqs 49 and 50. The gas flow in the porous electrode is described by the Brinkman equation, as shown in eqs 51 and 52.
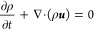
49
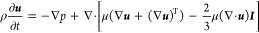
50
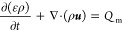
51
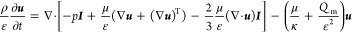
52where ***u*** is the velocity vector; μ is the dynamic viscosity of the gas mixture; ***I*** is the unit matrix; κ is the permeability of the porous media; and *Q*_m_ is the mass source term caused by chemical/electrochemical reactions.

#### 3.1.3 Governing Equations of Energy Conservation

Similarly, energy conservation is described for energy conservation in the flow channel, as expressed by eq 53, and energy conservation in the porous electrode, as expressed by eq 54.
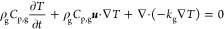
53
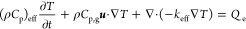
54where *C*_p,g_ is the specific heat capacity of the gas mixture, which is calculated using the mixture rule, as expressed by eq 55; ρ_g_ is the density of the gas mixture; *k*_g_ is the heat conductivity of the gas mixture, as expressed by eq 56; (*ρC*_p_)_eff_ is the heat capacity of the porous medium, determined using eq 57; *k*_eff_ is the effective heat conductivity of the porous medium, determined using eq 58; and *Q*_e_ is the heat source term.
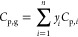
55
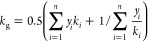
56
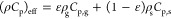
57
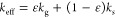
58

The thermophysical parameters of common anode, cathode, and electrolyte materials are summarized in Table 6.^33,112,113^

**Table 6:** Thermophysical Properties of Common Electrode and Electrolyte Materials

materials	specific heat capacity, *C*_p,s_ (J·kg^–1^·K^–1^)	heat conductivity, *k*_s_(W·m^–1^·K^–1^)	density, ρ_s_ (kg·m^–3^)
Ni/YSZ	390	6.23	6870
YSZ	525	2.57	6086
LSM/YSZ	398	3.47	3814.8
LSCF/GDC	430	6.0	3030

Owing to the different electrochemical processes occurring in the anode, cathode, and electrolyte, the heat generation rates in these regions are not the same. The electrochemical heat source terms for the anode *Q*_ea_, electrolyte *Q*_ee_, and cathode *Q*_ec_ are described by eqs 59–62,^33^ as follows:
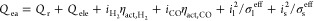
59
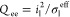
60
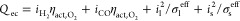
61
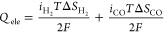
62where *Q*_r_ is the heat source generated from chemical reaction; *Q*_ele_ is the heat source generated from the electrochemical heat source including oxidation and reduction reaction heat; *i*_H_2__ and *i*_CO_ are the currents generated by H_2_ and CO electrochemical reactions, respectively; η_act, H_2__ and η_act, CO_ are the activation polarizations from the H_2_ and CO electrochemical reactions, respectively; *i*_l_ and *i*_s_ are the currents of the ionic and electronic conducting phases, respectively; σ_l_^eff^ and σ_s_^eff^ are the effective conductivities of the ionic and electronic conducting phases, respectively; *T*Δ*S* is the electrochemical irreversible heat source; and Δ*S* is the entropy change of the electrochemical reactions.

#### 3.1.4 Charge Transfer Governing Equations

Charge transfer can be divided into electronic and ionic charge transfers, which are described by eqs 63 and 64, as follows:
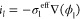
63
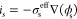
64where *i*_*l*_ and *i*_*s*_ are the currents of the ionic and electronic conducting phase, respectively; ϕ_l_ and ϕ_s_are the electric potentials of the ionic and electronic conducting phases, respectively.

#### 3.1.5 Electrochemical Reactions

The relationship between the electrode potential and current can be described by the Butler–Volmer equation, as shown in eq 65:
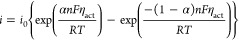
65where *i* represents the electrode current density; *i*_0_ represents the exchange current density; α represents the charge transfer diffusion coefficient; and *n* represents the number of electrons transferred in electrochemical reactions.

As a key parameter of electrochemical reaction kinetics, the exchange current density *i*_0_ represents the electrocatalytic activity of the electrode. The calculation method for *i*_0_ is not standardized given that the kinetics of electrochemical reactions are influenced by various factors, including the temperature, gas concentration, and cell-specific characteristics. Therefore, selection of the appropriate electrochemical reaction kinetics equation determines the accuracy of the SOFC model. Currently, the common method to calculate *i*_0_ is the Arrhenius equation, related to temperature and gas partial pressure, as shown in eqs 66 and 67:^114−116^
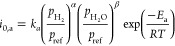
66
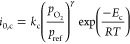
67where *k*_a_ and *k*_c_ are the coefficients of the anode and cathode, respectively; *E*_a_ and *E*_c_ are the activation energies of the anode and cathode, respectively; and α, *β,* and γ are exponential factors.

Bao et al.^10^ summarized typical electrochemical reaction kinetics equations and provided detailed parameter values. Although it can be employed as fuel for electrochemical reactions, CO is considered to be converted to H_2_ through the WGS reaction before participating in electrochemical reactions under a low-CO atmosphere. Therefore, considering the electrochemical reaction kinetics of CO in a low-CO atmosphere is unnecessary.^117−119^ Nevertheless, these kinetics should be considered when the WGS reaction cannot completely convert CO to H_2_ in a high-CO atmosphere, especially when syngas is the fuel. A detailed investigation of the electrochemical reaction kinetics of CO is as important as that of H_2_. Matsuzaki et al.^28^ reported that the electrochemical reaction rate of H_2_ was 2.3–3.1 times larger than that of CO. Currently, the electrochemical reaction kinetics of CO are mainly determined by assuming that the pre-exponential factor of CO is several times smaller than that of H_2_ (generally 2.5 times), or that the exchange current density generated by CO is several times smaller than that of H_2_ (generally 2.5 times). Xu et al.,^33,120,121^ Ni et al.,^122,123^ Andersson et al.,^124,125^ Bao et al.,^70^ and Zhu et al.^32,126^ employed such assumptions and obtained satisfactory simulation results.

The operating voltage *V* of the cell can be obtained by subtracting the polarization overpotentials from the thermodynamic equilibrium voltage (Nernst voltage, *E*), as shown in eq 68. The Nernst voltage can be calculated by eq 69 and eq 70. If only H_2_ participates in the electrochemical reaction, Nernst voltage is calculated by eq 69; if only CO participates in the electrochemical reaction, Nernst voltage is calculated by eq 70. If both H_2_ and CO are involved in electrochemical reaction, Nernst voltage can be calculated by eq 71.
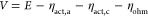
68
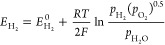
69
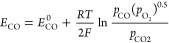
70
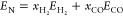
71where η_act,a_, η_act,c_ are the activation overpotentials of the anode and cathode, respectively; η_ohm_ is the ohm overpotential; *E*_H_2__^0^ and *E*_CO_^0^ are the H_2_ and CO standard electrode potentials, respectively.

#### 3.1.6 Chemical Reactions

The fuel flexibility of SOFCs means that they can be operated with fuels other than H_2_ and CO, which are typically reformed and converted into H_2_ and CO for subsequent electrochemical reactions. Therefore, the kinetics equations for fuel reforming and WGS reactions are incorporated into SOFC MPMs. The common reaction kinetics equations for different fuels used in SOFC simulations are summarized in Table 7. Notably, various reforming reaction kinetics equations have been reported for some fuels owing to differences in experimental setups, reaction kinetics models, and other factors. For instance, > 20 different CH_4_ reforming kinetics equations have been reported in the literature. Faheem et al.^127^ provided a detailed summary of these kinetics equations, including type, applicable temperatures, anode materials, steam-to-carbon ratio (S/C), and related parameters. To deeply understand the reaction process and accurately describe the reaction kinetics, some researchers have investigated the elementary reactions of CH_4_, NH_3_, and other fuels during reforming (reviewed in detail in Section 4). Here, we summarize the most commonly used equations for fuel reforming kinetics in SOFC modeling.

**Table 7:** Commonly Used Equations of Fuel Reforming Kinetics in SOFC Modeling^a^

fuel type	chemical reaction equations	reaction kinetics	remark	refs.
CH_4_ or Syngas	MSR:CH_4_ + H_2_O → CO + 3H_2_		Methane carbon dioxide reforming is considered. The MSR and WGS reaction kinetics adopt the Arrhenius model. The MCDR kinetics adopt the LHHW kinetics model.	(32, 128, 129)
	WGS:CO + H_2_O → CO_2_ + H_2_			
	MCDR:CH_4_ + CO_2_ → 2CO + 2H_2_			
				
		*K*_MSR_ = 1.0267 × 10^10^ exp (−0.2513*Z*^4^ + 0.3665*Z*^3^ + 0.5810*Z*^2^–27.134*Z* + 3.277)		
		*K*_WGS_ = exp (−0.2935*Z*^3^ + 0.6351*Z*^2^ + 4.1788*Z* + 0.3169)		
				
				
				
CH_4_ or Syngas	MSR: CH_4_ + H_2_O → CO + 3H_2_		The specific surface area of the catalyst is required to convert the reaction rate unit. η is the catalyst effectiveness factor, which can be found in ref.^130^*k* is reaction rate constant.	(32, 131−133)
	WGS: CO + H_2_O → CO_2_ + H_2_			
	RM: CH_4_ + 2H_2_O → CO_2_ + 4H_2_			
				
Methanol	MDR: CH_3_OH → CO + 2H_2_	*R*_MDR_ = *kp*_CH_3_OH_*E*	The WGS reaction rate expression is the same as that in the second row. *k* is the tuning parameter for model validation.	(33, 121, 134)
	WGS: CO + H_2_O → CO_2_ + H_2_			
Ethanol	EDR: C_2_H_5_OH → CH_4_ + CO + H_2_	*R*_EDR_ = *K*_EDR_*p*_*E*_	*p*_E_ is the ethanol partial pressure. The MSR and WGS kinetics are the same as those in the second row.	(36, 135)
	MSR: CH_4_ + H_2_O → CO + 3H_2_			
	WGS: CO + H_2_O → CO_2_ + H_2_			
Ammonia	AD: 2NH_3_ → N_2_ + 3H_2_		*p* is the gas partial pressure.	(136−139)
Ammonia	AD: 2NH_3_ → N_2_ + 3H_2_		The first equation is applicable to cracking NH_3_ on Ni–YSZ, and the second equation is applicable to cracking NH_3_ on Ni mesh. The meanings and values of the parameters can be found in the literature.	(140,141)
				
Carbon	BR: C + CO_2_ → 2CO		The value of parameters *K*_1_-*K*_6_ can be found in ref.^142^ The WGS kinetics are the same as those in the second row.	(142−144)
	WG: C + H_2_O → CO + H_2_			
	WGS: CO + H_2_O → CO_2_ + H_2_			
Glycerol	GDR: C_3_H_8_O_3_ → 3CO + 4H_2_		*A*_s_ is the catalyst’s metal surface area. The WGS kinetics are the same as those in the second row.	(145, 146)
	WGS: CO + H_2_O → CO_2_ + H_2_			
Biomass	MSR: CH_4_ + H_2_O → CO + 3H_2_	Same as the first row	The gasification unit is integrated with the SOFC stack. Biomass is converted to syngas through the gasification reaction. The syngas is used as the inlet fuel for the SOFC.	(43, 147)
	WGS: CO + H_2_O → CO_2_ + H_2_			

a MSR, methane steam reforming; WGS, water–gas shift; MCDR, methane carbon dioxide reaction; RM, reverse methanation; EDR, ethanol decomposition reaction; AD, NH_3_ decomposition; BR, Boudouard reaction; WG, water gasification; GDR, glycerol decomposition reaction.

The above equations have been utilized to construct the reaction-transfer-conservation MPM framework of SOFCs. Subsequent models have been developed based on extension and modification of the current model framework, such as stress models, carbon deposition models, etc.

### 3.2 Research Progress of Cell/Stack Models

SOFC cell/stack models can be categorized as steady-state and transient models. Steady-state models do not consider transient/dynamic terms in the governing equations, making transient models special cases. These models address different aspects of SOFC behavior. Steady-state models focus on heat and mass transfer mechanisms, structure optimization, fuel flexibility, and stress distribution. By contrast, transient models investigate dynamic performance, durability, start-up behavior, and other time-dependent aspects of SOFC operation. The following sections provide a comprehensive overview of the applications of SOFC cell/stack models, aiming to identify current challenges and future directions in the field.

#### 3.2.1 Development of the Steady-State Models

##### 3.2.1.1 Heat and Mass Transfer Mechanisms

Research on the heat and mass transfer mechanisms of SOFCs primarily relies on developing MPMs. The models are employed in parametric analyses, which help in understanding the internal physical mechanisms of heat and mass transfer. Additionally, these models are utilized to optimize operating parameters and structural configurations. Consequently, the models employed to study the heat transfer mechanisms of SOFCs align closely with the framework outlined in Section 3.1.

**Figure 18:**
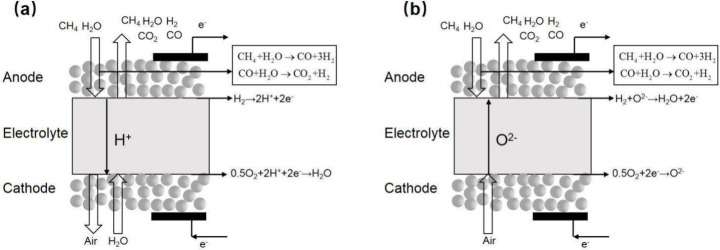
Schematic of (a) the SOFC-H structure and (b) the SOFC-O structure. Reproduced with permission from ref (30). Copyright 2008 Elsevier.

Ni et al.^30^ developed a 1D MPM for an SOFC using CH_4_ as the fuel, comparing the performance of the SOFC with proton-conducting electrolyte (SOFC-H) and O^2–^-conducting electrolyte (SOFC-O). The model focused on the diffusion and reaction of the PEN cross-section along the electrode thickness without considering momentum transfer, as depicted in Figure 18. Typically, SOFCs employ O^2–^-conducting electrolytes; however, research on proton-conducting electrolytes for SOFCs is ongoing. The key distinction between SOFC-O and SOFC-H lies in the type of electrolyte utilized for ionic conduction. The electrode reactions for SOFC-O are expressed by eqs 1–3, while those for SOFC-H are expressed by eqs 72 and 73. SOFC-H can only utilize H_2_ as the fuel for electrochemical reactions, producing H_2_O at the cathode. The results of the study indicated that the performance of SOFC-H was significantly lower than that of SOFC-O owing to its higher ohmic overpotential. The study also explored the effects of temperature and porosity on cell performance. Ni^29^ further developed a 2D planar single-channel SOFC multiphysics model fueled by CH_4_ to investigate heat and mass transfer mechanisms. This model considered methane steaming reactions, an electrochemical model, mass transfer, momentum transfer, and energy conservation and examined the effects of operational potential and inlet gas velocity on cell performance.
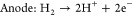
72
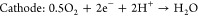
73

**Figure 19:**
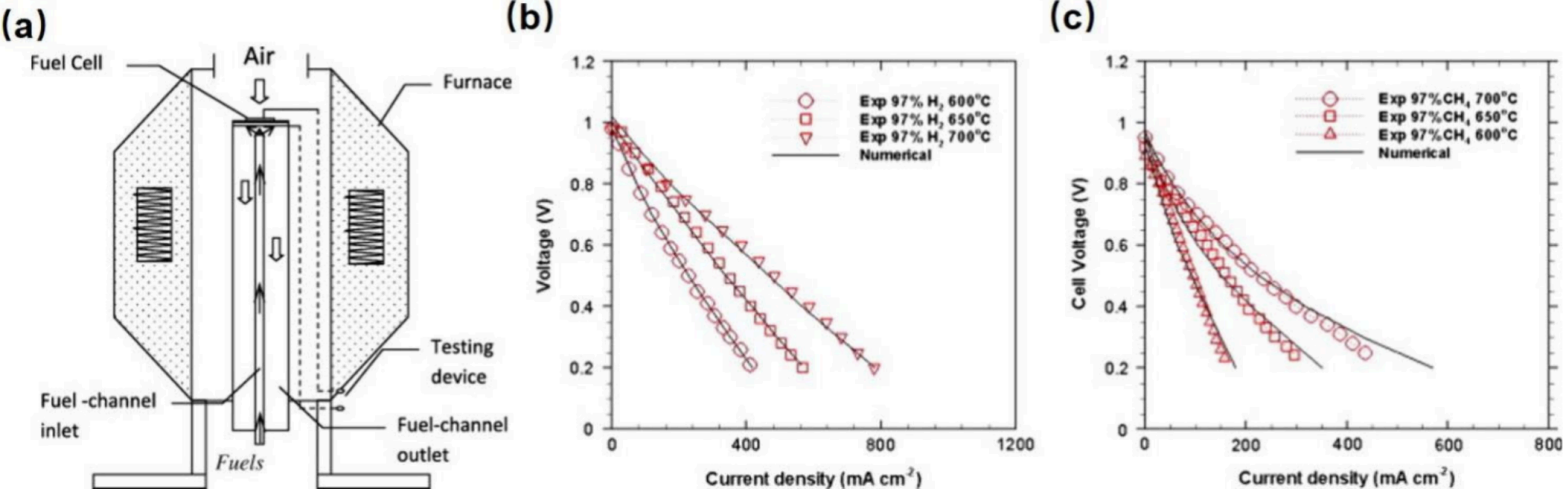
(a) Experimental test bench of a button cell; (b) comparison of experimental and model *I–V* curves of a button cell fueled by humidified H_2_; (c) comparison of experimental and model *I–V* curves of a button cell fueled by humidified CH_4_. Reproduced with permission from ref (149). Copyright 2013 Elsevier.

Hussain et al.^148^ developed a 2D electrode–electrolyte-assembly model, similar to the structure shown in Figure 18, without considering the gas flow process in the flow channel. The novelty of this model lay in dividing the electrode into reaction and support layers, wherein electrochemical reactions occurred in the reaction layer, while reforming and WGS reactions occurred in the support layer. This study carefully analyzed the effects of three kinds of overvoltage (activation, concentration, and ohmic) on electrochemical performance. Xie et al.^149^ developed a 2D button cell model using syngas as the fuel and considered the electrochemical oxidation process of H_2_ and CO. The model was validated using a self-built cell test bench, as depicted in Figure 19(a). The authors compared the measured and computed *I*–*V* curves of SOFCs fueled by humidified H_2_ and CH_4_ at different temperatures, as illustrated in Figure 19(b, c). This study also presented the molar fraction distributions of various gases, pressure, and field spatial distributions. The authors concluded that anode reaction processes were slower than the cathode O_2_ reduction process in direct CH_4_-fueled SOFCs. Alhazmi et al.^150^ established a 3D button cell model using H_2_ as the fuel and validated it using a self-built experimental bench. The experimental *I*–*V* curves indicated good agreement with the simulation results. The study also explored the effects of temperature and H_2_ and O_2_ flows on current density. The same framework was employed in the above-mentioned studies. First, the MPM was established, and then the distributions of concentration, temperature, and reaction rate were analyzed to explore the reaction-transfer mechanism. The research development was very clear, from 1D to 3D, from the PEN to single cell or button cell, and from simple to complex.

To address the previous limitation of validating SOFC models only against *I*–*V* curves, Aydın et al.^151^ developed a model of a 2D tubular SOFC fueled by H_2_. The authors constructed a tubular SOFC test bench to validate the *I*–*V* curves and temperature field, ensuring the model’s reliability. Measurements of current density and temperature were conducted midstream, upstream, and downstream of the SOFC, and the *I*–*V* curves and temperatures in these regions were compared between experimental and simulation data. The study findings highlighted the considerable influence of radiant heat transfer on the spatial distribution of temperature gradients in microtubular SOFCs. Wang et al.^152^ developed a 2D model of a button cell fueled by CH_4_. The numerical simulations revealed that the OCV was sensitive to CH_4_ steam reforming (MSR) kinetics for low-steam CH_4_ fuel. The authors improved the MSR kinetics equation by comparing experimental and simulation OCV values, demonstrating that its use without additional parameter adjustments yielded *I*–*V* curves that closely matched the experimental data. Mirahmadi^153^ validated *I*–*V* curves and temperature distribution through experimental data. Notably, experimental validation of temperature field simulation results is rare owing to the inherent challenges of in situ temperature measurements.

Because the equations utilized to describe electrochemical reaction kinetics are not unique and such kinetics are affected by several factors, investigating the kinetics of different electrochemical reactions is necessary to ensure model reliability. Takino et al.^154^ reported that the electrode reaction kinetics equation for humidified H_2_ was not suitable to describe the electrochemical oxidation of H_2_ under a prereformed CH_4_ atmosphere. The authors experimentally determined an anode exchange current density suitable for the prereformed CH_4_ gas atmosphere, modifying the anode exchange current density equation to consider the effect of the S/C ratio. The related parameter values in the equation differed when the S/C ratio = 2.0 or 2.5. The modified expression of the electrochemical reaction kinetics is given by eq 74. The modified model was obtained by applying the modified equation to an existing SOFC MPM. The prediction reliability of the *I*–*V* curves and temperature distribution using the modified model was significantly better than that using the previous model. Zhang et al.^155^ developed MPMs with different electrode reaction kinetics to evaluate the influence of the three most frequently used electrode reaction kinetics on SOFC models. Their study provided a reference for selecting the appropriate kinetics equation for electrochemical reactions in SOFC modeling.
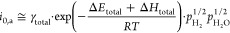
74where γ_total_ = 401 and Δ*E*_total_ + Δ*H*_total_ = 4.4 × 10^4^ J · mol^–1^at S/C = 2.0; γ_total_ = 262, and Δ*E*_total_ + Δ*H*_total_ = 4.0 × 10^4^ J · mol^–1^ at S/C = 2.0.

To enhance the reaction-transfer process and improve cell performance, several studies have developed models through parametric analysis. For example, Sayadian et al.^156^ constructed an MPM of a 2D planar proton-conducting SOFC. The authors derived the dimensionless forms of the governing equations and specified the dimensionless parameters affecting the power generation performance of the SOFC, including the Sherwood, Peclet, Reynolds, Darcy, Butler–Volmer, first Damkohler, and third Damkohler numbers. Subsequently, they investigated the influence of these dimensionless parameters on electrochemical performance through parametric analysis. Zhu et al.^32^ constructed a 2D planar SOFC MPM using syngas derived from biomass gasification as the fuel. The authors explored in detail the influences of the gasification syngas composition, operating voltage, temperature, fuel flow rate, and other operating parameters on the internal reaction, species distribution, and current density distribution. Dang et al.^157^ established a 3D single-channel SOFC model with H_2_ as the fuel. The authors investigated the influence of the operating and structural parameters, such as cathode flow rate and porosity, on electrochemical performance. Andersson et al.^124^ developed a model of a 2D planar SOFC fueled by syngas, considering the electrochemical oxidation reaction of CO. This model was employed to analyze the gas molar fraction, current density, and Nernst voltage distributions, shedding light on their coupling mechanism. Mirahmadi^153^ developed a tubular SOFC model and constructed a corresponding test bench to study the thermal effects of tubular SOFCs. The authors investigated the influences of direct and gradual internal reforming on temperature distribution within the SOFC. In gradual internal reforming, insufficient steam supply led to a slower steam reforming reaction rate than in direct internal reforming, which reduced the temperature gradient in the SOFC anode. The simulation results indicated that the axial temperature distribution was more stable with gradual internal reforming than with direct internal reforming. While the ohmic polarization of pure H_2_ was lower than that of CH_4_ during internal reforming, the concentration loss of pure H_2_ was larger. Jeon^158^ developed a comprehensive overpotential 3D model for an intermediate-temperature anode-supported SOFC using open-source fuel cell code developed by OpenFOAM. The model was validated using experimental data from various sources. While the *I*–*V* curves from this model did not match some experimental data in the literature, the author provided a detailed explanation and analysis. Additionally, the author employed the validated model to determine the contributions of different overpotentials to the total overpotential. The study reported that the anode overpotential was greatest at 800 °C, the cathode overpotential was dominant at 600 °C, and the contributions of the anode/cathode/electrolyte overpotentials to the total overpotential were similar at 700 °C. Finally, the study investigated the feasibility of improving the electrochemical performance of intermediate-temperature stacks by adjusting electrolyte thickness and replacing the electrolyte to reduce electrolyte overpotential.

**Figure 20:**
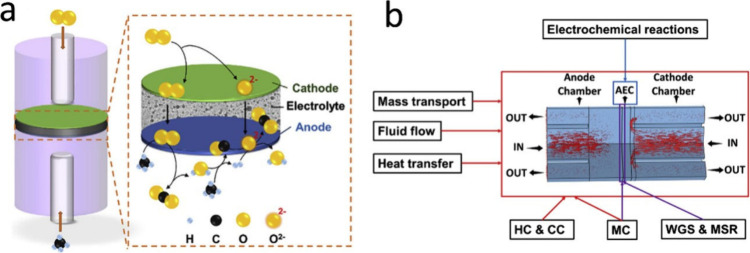
(a) Schematic of a button cell with all-porous structure; (b) geometric structure of the all-porous cell for modeling. Reproduced with permission from ref (31). Copyright 2019 Elsevier.

To prevent SOFC carbon deposition, Guo et al.^159^ proposed an all-porous SOFC, which was later simulated by Xu et al.^31^ The model’s geometric structure is illustrated in Figure 20. The all-porous SOFC design allowed O^2^ transfer from the cathode to the anode chamber, inhibiting carbon formation. Therefore, in addition to MSR and WGS, the model considered the oxidation reactions of H_2_, CH_4_, and CO. Xu et al.^31^ developed an MPM of an all-porous SOFC and studied the effects of operating voltage, inlet gas temperature, electrolyte porosity, support layer type (anode or electrolyte), and inlet gas concentration on electrochemical and thermal performance.

Commercial SOFCs are presented as stacks; thus, the ultimate goal of numerical simulations is to establish a practical stack model, analyze the operating mechanism of the stack, and optimize performance. Li et al.^160^ developed a 3D single-channel SOFC model with H_2_ as the fuel. The authors explored the effects of interconnector width, cathode substrate thickness, and cathode stoichiometry on electrochemical performance and temperature distribution. To improve parameter uniformity in the cross-multichannel direction, the authors simulated the performance of a 67.2 cm^2^ SOFC stack considering side cooling. Guo et al.^161^ developed a kW-class planar SOFC stack model using H_2_ and CH_4_ as the fuels. The model included all of the physical processes described in Section 3.1, namely, chemical/electrochemical reactions, mass/momentum transfers, and energy conservation. Notably, the gas flow in the channel was described by the *k*–ε turbulence model and the electrochemical process was described by the *I*–*V* relationship instead of the Butler–Volmer equation. To reduce the computational load, half of the stack was taken as the computational domain by adopting a symmetric boundary. The model was solved in approximately 10 h using a small 2-CPU/16-core workstation. Through this model, the authors studied the temperature, species, current density, and voltage distributions, flow uniformity, and energy conversion efficiency characteristics of the stack. Li et al.^162^ constructed a 3D planar SOFC stack MPM consisting of 30 cells, which was solved in approximately 40 h. The model provided the distributions of fuel gas pressure, temperature, current density, and fuel gas species in the 3D stack. The findings of the study emphasized the necessity to model the actual stack, given that simplifying the stack geometry or reducing multiphysics coupling was unreliable. Ding et al.^163^ constructed a 5-cell SOFC stack MPM with interdigital fuel channels to capture the internal work details. The model successfully obtained the gas molar fraction, temperature, electrochemical active site, and current distributions in the stack. This model was an important step in optimizing stack component structures to balance the physicochemical processes. As is evident from the aforementioned research, stack simulations offer a more precise representation of the actual SOFC structure but substantially prolong the computation time. This is not conducive to computer-aided optimization and design of stacks. Therefore, reducing the computational load and time during stack simulations is essential. This point is discussed in detail in Section 3.3.

Research on the heat and mass transfer mechanisms of SOFCs is relatively mature, and many studies have been published to date. The representative studies have been elaborated upon in detail above. To facilitate comparative analysis, the main points of these studies are listed in Table 8, which tabulates simulations involving various geometric structures, including 2D cross sections, button cells, tubular cells, 2D/3D single channels, and 3D stacks. However, different model types have different concerns. For example, the studies investigating button cells and micro tubular cells mainly compared simulation and experimental results because button cells are easily fabricated and tested;^149−151,153^ the 2D cross-section studies mainly focused on the mass transfer process and ignored the flow process;^30,148^ the studies describing single-channel cell simulations focused on the spatial distributions of parameters;^32,124,156,157,160^ and the stack-related studies focused on actual stack performance.^161−163^ Additionally, some studies focused on the applicability of different electrochemical/chemical reaction kinetics in SOFC models,^152,154,155^ which is important because these kinetics directly affect the mass transfer source term and electrochemical performance. The selection of suitable and reliable reaction kinetics remains challenging in SOFC modeling.

**Table 8:** Representative Studies on SOFC Heat and Mass Transfer Mechanisms

authors	cell type	dimension	fuel	CO electro-oxidation	radiation	mass transfer	energy conservation	software	validation	main concerns	ref
Ni et al.	Cross-section	2D	CH_4_	No	No	DGM	No	-	Model	SOFC-O and SOFC-H performance comparison and parameter analysis on porosity and temperature.	(30)
Ni	Single channel	2D	CH_4_	No	No	DGM	Yes	FORTRAN	Model	Heat and mass transfer performance investigation and parameter analysis on potential and gas velocity.	(29)
Hussain et al.	Cross-section	2D	CH_4_	No	No	DGM	Yes	-	Experimental	Effects of temperature, chemical reactions, anode reaction layer thickness, tortuosity, and volume fraction of electron-conducting particles on *I–V* curves and overpotential.	(148)
Xie et al.	Button cell	2D	Syngas^a^	Yes	No	DGM	Yes	COMSOL	Experimental	A button cell model was established, and the model was validated using a self-built cell test bench.	(149)
Aydın et al.	Tubular cell	2D	H_2_	No	Yes	SMM	Yes	COMSOL	Experimental	A 2D tubular SOFC model was constructed. The *I*–*V* curves and temperature distribution were verified using the self-built experimental test bench.	(151)
Andersson et al.	Single channel	2D	Syngas	Yes	No	DGM	Yes	COMSOL	-	A 2D planar SOFC model was established, and the oxidation reaction of CO was considered.	(124)
Wang et al.	Button cell	2D	CH_4_	No	No	DGM	Yes	COMSOL	Experimental	The MSR kinetics with low-steam CH_4_ were corrected by comparison of experimental and simulation OCV values.	(152)
Xu et al.	Button cell	2D	CH_4_	Yes	No	DGM	Yes	COMSOL	Experimental	An MPM of an all-porous SOFC was developed and the effects of parameters on electrochemical and thermal performance were investigated.	(31)
Li et al.	Single channel and multichannel	3D	H_2_	No	No	DGM	Yes	COMSOL	Experimental	3D single-channel and 3D multichannel SOFC models were developed. The effect of side cooling on the uniformity of stack parameter distribution was explored.	(160)
Takino et al.	Single channel	3D	Prereformed gas^b^	No	No	SMM	No	COMSOL	Experimental	The reaction kinetics of existing humidified H_2_ were modified experimentally to make them more suitable for the electrochemical oxidation process under a prereformed CH_4_ gas atmosphere.	(154)
Zhang	Single channel	3D	H_2_	No	No	SMM	Yes	COMSOL	-	The effects of three frequently used H_2_ electrode reaction kinetics were compared for an SOFC model.	(155)
Alhazmi	Button cell	3D	H_2_	No	No	SMM	Yes	Fluent	Experimental	A 3D button cell model was established, and the *I*–*V* curves were validated using a self-built experimental bench.	(150)
Guo et al.	Planar stack	3D	H_2_ and CH_4_	No	No	-	Yes	Fluent	Experimental	A 3D kW-class planar stack SOFC model was developed. The important parameters were investigated.	(161)
Li et al.	Planar stack	3D	H_2_ and CH_4_	No	No	-	Yes	Fluent	Experimental	A 3D stack model was constructed to model the actual stack.	(162)
Ding et al.	Planar stack	3D	H_2_	No	No	DGM	Yes	-	Experimental	A 3D actual stack model was constructed and the distributions of important parameters were investigated.	(163)
Sayadian et al.	Single channel	2D	Syngas	No	No	DGM	Yes	In-house code	Experimental	A 2D single-channel proton-conducting SOFC model was constructed. The dimensionless forms of the governing equations were derived and the influence of the dimensionless numbers on the electrochemical performance of the SOFC was studied.	(156)
Zhu et al.	Single channel	2D	Syngas	Yes	No	DGM	No	COMSOL	Experimental	A 2D planar isothermal model was established to study the influences of the operating parameters on the current density and species concentration distributions.	(32)
Dang et al.	Single channel	3D	H_2_	No	No	SMM	Yes	-	Experimental	Construction and parametric analysis of 3D single-channel model.	(157)
Mirahmadi et al.	Tubular cell	2D	H_2_ and CH_4_	No	Yes	FM	Yes	FEMLAB	Experimental	A 2D tubular SOFC model was established and an experimental testing workbench was built. The temperature field was experimentally validated.	(153)
Jeon	Planar stack	3D	H_2_	No	No	DGM	Yes	OpenFOAM	Experimental	The 3D stack model was developed based on open-source code and validated by comparison with different experimental data.	(158)

a Syngas represents a gas mixture of H_2_, CH_4_, CO, H_2_O, and CO_2_.

b The prereformed is a gas mixture of H_2_, CO, H_2_O, and CO_2_.

Validating models through experimental data is common practice in simulation-related research; however, the rationale and reliability of such validations need to be strengthened. Experimental data reported in the literature often lack clear geometric structures and operating conditions, limiting their use for model validation. Ensuring matching experimental and simulation conditions is crucial for model validation. For example, Jeon^158^ constructed a 3D stack model, which was validated using experimental data from Zhao et al.^164^ However, Zhao et al.^164^ only provided the *I*–*V* curves of the button cell and thicknesses of the electrode and electrolyte without specifying the cell’s radius or arrangement of the flow channels. Jeon^158^ did not elaborate on how these experimental conditions were applied to validate the planar stack model, raising concerns about the validation process. Furthermore, most existing models focus on comparing *I*-*V* curves, without validating the species concentration and temperature distributions, which reduces the overall credibility of the models to some extent. Given the current difficulty of measuring the internal parameters of SOFCs in situ, addressing this issue should be prioritized in future research. Fortunately, numerical simulation researchers have increasingly begun to verify their models through self-built experimental platforms, greatly promoting model validation.^149−151,153^ Furthermore, SOFC experimental researchers are encouraged to disclose as comprehensively as possible the cell geometric structure, experimental conditions, and other pertinent data to provide a reliable foundation for the validation of numerical simulations, thereby fostering synergy between experimental and simulation-based research efforts.

At present, the commercial software for SOFC MPMs mainly includes Fluent and COMSOL Multiphysics. Fluent is preferred for large stacks owing to its short solution time and good convergence, while COMSOL is favored for small cell mechanism analysis owing to its convenience. Fluent performs calculation based on the FVM, which is mainly utilized in heat transfer and fluid simulations. On the basis of traditional heat transfer and flow modules, electrochemical, chemical, and mass transfer modules can be added to efficiently simulate fuel cells. COMSOL employs the finite element method (FEM) to discretize the governing equations, which is suitable for calculating coupling simulations between various physics fields and has a wide range of applications. Although COMSOL can easily simulate the multiphysics coupling phenomenon, its computational efficiency is not high. Despite the considerable progress achieved in simulating SOFCs to explore the reaction-transfer mechanism, the following limitations need to be addressed in future research:

- Identifying the applicable scenarios for different chemical/electrochemical reaction kinetics. Selecting the appropriate chemical/electrochemical reaction kinetics can enhance the accuracy and reliability of SOFC MPMs.
- Evaluating the effects of current simplifications (including assumptions and geometric structure simplifications) on the modeling results to determine the rationality of these simplifications.
- Validating not only the *I*–*V* curves of cells but also the species concentration and temperature distributions for comprehensive model validation.
- Developing an SOFC stack model and utilizing the simulation results to aid the design and manufacturing process and optimize cell performance.

##### 3.2.1.2 Structure Optimization

The purpose of optimizing the SOFC structure can be summarized in two main points: (1) optimizing mass transfer to reduce concentration polarization and species distribution nonuniformity; and (2) optimizing heat transfer to reduce temperature gradients and avoid mechanical failure. Additionally, much structure optimization work focuses on modifying the flow channel given that the structure of the PEN is relatively fixed.

**Figure 21:**
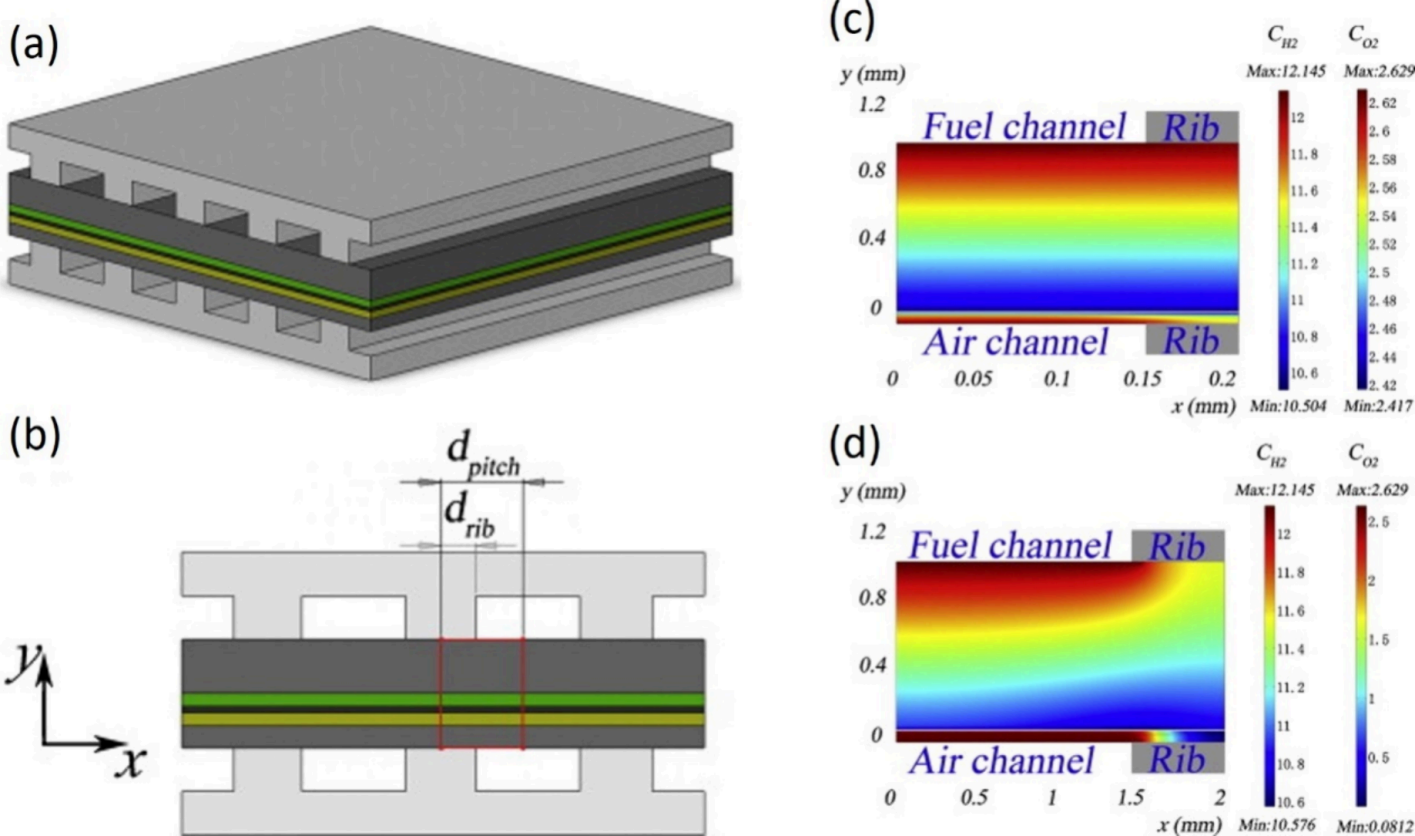
Schematic of (a) an anode-supported planar SOFC stack; (b) cross-section of the stack; (c) distributions of H_2_ and O_2_ concentrations in a single cell; (d) distributions of H_2_ and O_2_ concentrations in the stack. Reproduced with permission from ref (165). Copyright 2012 Elsevier.

Lin et al.^165,166^ developed a PEN cross-section model to explore the influence of rib shape on cell performance. The authors found that the O_2_ concentration in the lower end area was very low when the rib width was too large, resulting in poor local performance that affected the average cell performance, as illustrated in Figure 21. The output current density of the stack was 6011 A·m^–2^, which was 19% lower than the current density of a single cell. To identify the optimal rib width, the output current density under different rib widths was given, and the function between the optimal rib width, electrode–interconnector contact area specific resistance (ASR), and pitch was fitted.

**Figure 22:**
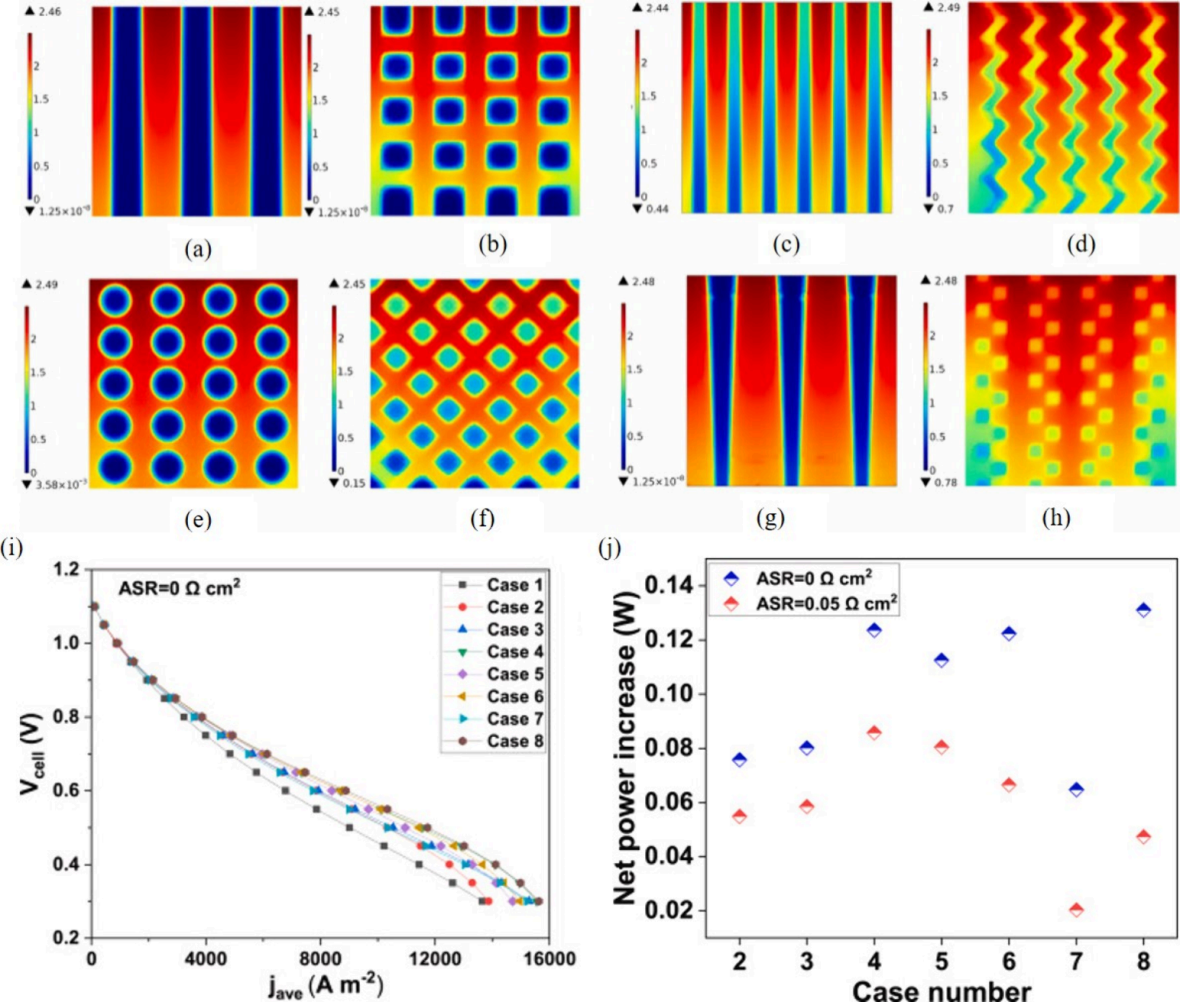
Distributions of O_2_ concentrations at the cathode–electrolyte interface of SOFC stacks with (a) conventional straight channel, (b) discrete rectangular ribs; (c) discrete cylindrical ribs; (d) discrete rhombus ribs; (e) straight flow channels; (f) corrugated ribs; (g) trapezoid ribs; and (h) crisscross rectangular ribs; (i) *I*−*V* curves without considering the effect of ASR; (h) net power gain. Reproduced with permission from ref (167). Copyright 2022 Elsevier.

Guo et al.^167^ proposed different interconnector structures to achieve uniform gas distribution and improve SOFC stack performance, as illustrated in Figure 22. The MPMs of these different flow channel structures were established to evaluate the influence of flow channel design on SOFC performance. Compared to the traditional flow channel, the proposed novel flow channel greatly improved the uniformity of O_2_ distribution at the cathode and the peak power density could be increased by 27.86%. The improvement in stack performance was attributed to the increased O_2_ concentration below the rib. Additionally, the power loss due to the pressure drop caused by the new rib design accounted for only 0.1% of the total increased electric power, making the power loss caused by the pressure drop negligible. Saied et al.^168^ designed helical, single-entry serpentine, traditional parallel, modified parallel, double-entry serpentine, and triple-entry serpentine novel flow channels for planar SOFC stacks. Subsequently, MPMs were developed to compare the performance of stacks with different flow channels. The simulation results indicated that a backflow phenomenon occurred at the outlet side of the cathode owing to the premature consumption of fuel in the single-inlet channel design (including the serpentine flow channel). The backflow could be avoided by increasing the number of ports at the inlets and outlets, which increased the inlet gas mass flow rate. Additionally, the three-inlet serpentine channel exhibited more uniform fuel and O_2_ distributions than the other channel structures, with a current of up to 23.3 A, which was 5.18% higher than that of the other structures. The findings of the study indicated that the SOFC stack achieved optimal performance.

**Figure 23:**
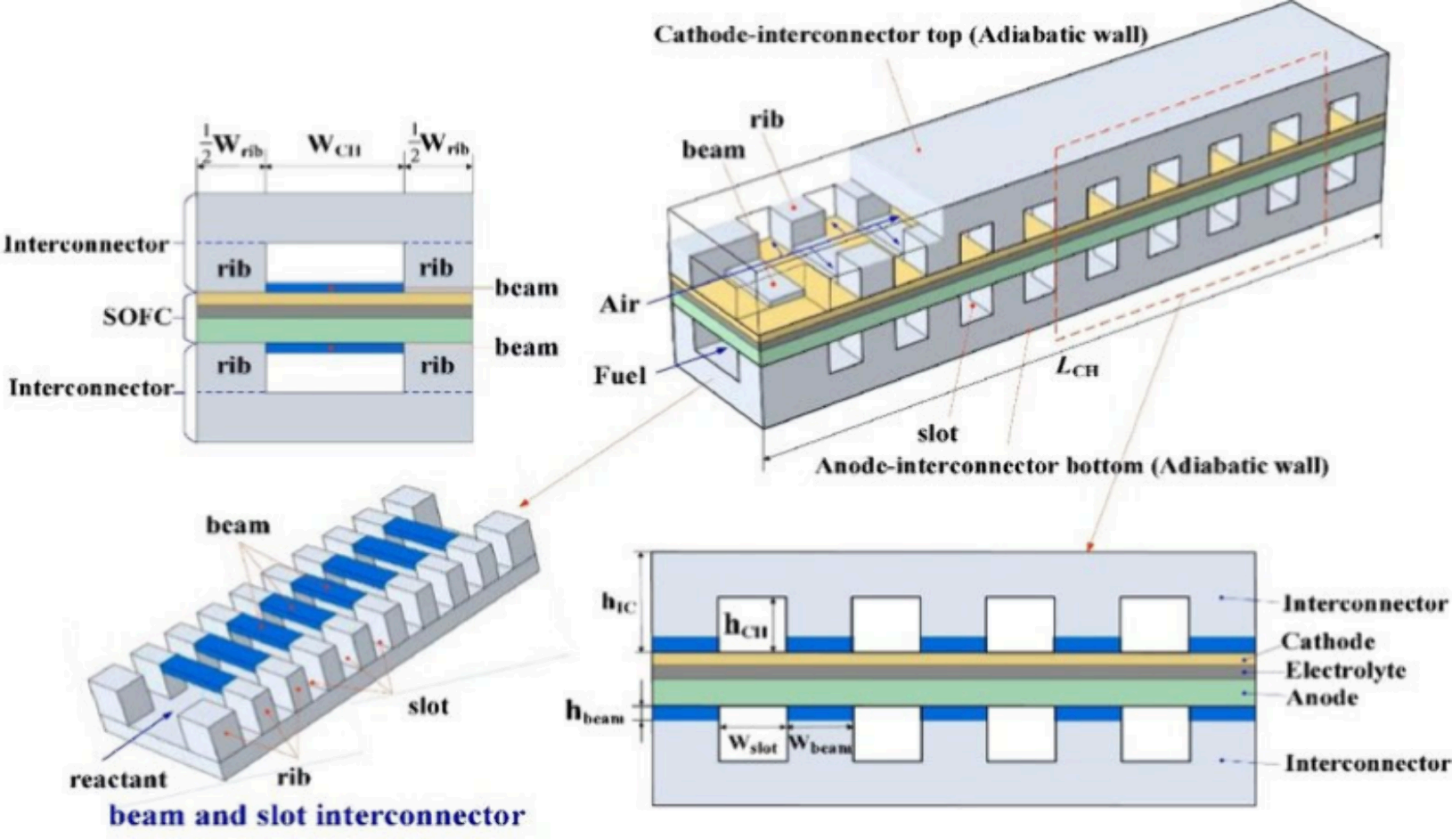
Schematic of the novel beam and slot interconnector designed by Fu et al. Reproduced with permission from ref (169). Copyright 2021 Elsevier.

Fu et al.^169^ proposed a novel beam and slot interconnector design, as illustrated in Figure 23. The authors developed a 3D MPM and validated it using a self-built test platform. The transfer characteristics and electrochemical performance of a conventional stack with straight channels were compared with those of a stack with the novel interconnector. The results indicated that the novel interconnector could reduce the gas diffusion resistance, shorten the charge transport path, and decrease the contact resistance between the electrode and interconnector. In terms of electrochemical performance, the activation, concentration, and contact overpotentials of the SOFC with the novel interconnector could be reduced by 8.5%, 47.1%, and 96.4%, respectively, compared to those of the conventional interconnector under the operating conditions of 700 °C at 0.5 A·cm^–2^. The contact overpotential refers to the potential loss caused by resistance between the interconnector and anode support layer (ASL), interconnector, and cathode current collector layer. The total polarization loss could be reduced by 20% using the novel beam and slot interconnector.

**Figure 24:**
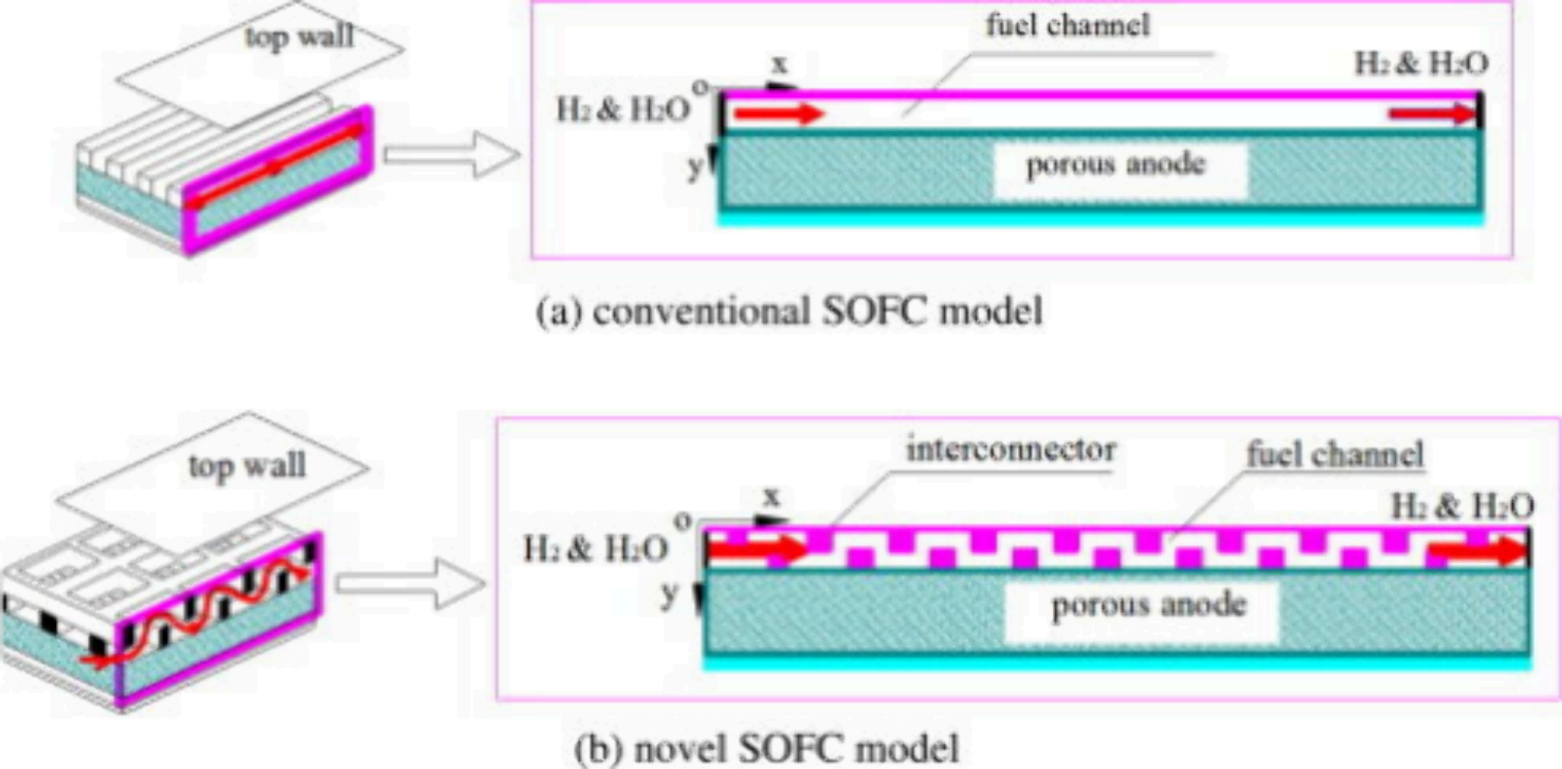
(a) Conventional SOFC; (b) novel SOFC with bi-layer interconnector. Reproduced with permission from ref (170). Copyright 2011 Elsevier.

Chen et al.^170^ introduced a novel double-layer interconnector SOFC structure, as depicted in Figure 24, and developed a half-cell model, comparing the performance of the novel SOFC with that of a traditional SOFC. The simulation results demonstrated that the novel interconnector could increase the gas flow velocity and H_2_ molar fraction at the porous anode, thereby reducing concentration polarization. Specifically, the concentration overpotential of the SOFC with the novel interconnector was reduced by 5% compared to that of the conventional SOFC. Zhao et al.^171^ designed three different SOFC gas inlet ducts: top-inlet, center inlet, and the buffer chamber inlet. They constructed a computational fluid dynamics model of the stack and analyzed the flow field inside the stack in detail. The simulation results indicated that the manifold geometry substantially influences the uniformity of the flow field. The buffer chamber inlet can redistribute the gas to improve flow uniformity. Optimizing the manifold geometry can reduce the flow velocity ratio, indicating improved flow distribution uniformity. Further, Zhao et al. also^172^ designed three different inlet tube shapes, including a straight inlet tube, flared inlet tube, and round perforated sheet manifold, and developed a half-cell model to study the flow field uniformity and pressure distribution.

**Figure 25:**
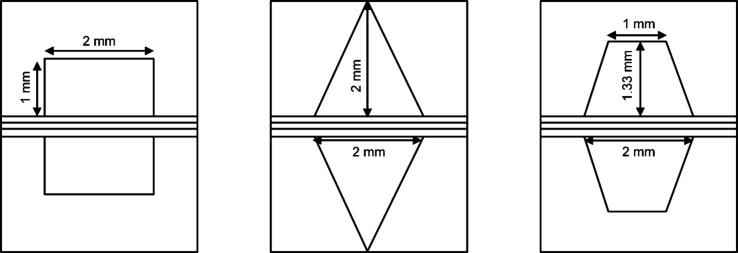
Cross sections of rectangular, triangular, and trapezoidal single-flow channel SOFCs. Reproduced with permission from ref (173). Copyright 2017 Elsevier.

Khazaee et al.^173^ compared the H_2_ molar distribution along the cell length and *I–V* curves of a planar SOFC with rectangular, triangular, and trapezoidal flow channel shapes via simulations, as illustrated in Figure 25. The simulation results indicated that the rectangular flow channel exhibited the highest H_2_ concentration along the cell length and best electrochemical performance, while the trapezoidal flow channel demonstrated the worst performance under the same conditions. The peak power density of the SOFC with a rectangular flow channel was approximately 27.6% higher than that of the SOFC with a trapezoidal flow channel. Notably, this study did not report the O_2_ concentration distributions in different flow channels, making it difficult to quantify the influence of the flow channel structure on O_2_ concentration distribution uniformity. However, the previous discussion made by Guo et al.^167^ has suggested that O_2_ concentration distribution has an important effect on cell performance. Manglik et al.^174^ compared the heat transfer characteristics of these three flow channel structures, reporting that, among the three flow channel shapes, the rectangular flow channel provided better cooling of the planar stack, resulting in more uniform temperature distribution of the SOFC. Andersson^175^ compared the performance of three rectangular channels with different sizes to study the influence of channel size on the cell’s heat and mass transfer performance. The results showed that the wider and thinner channel structure promoted gas flow, improved current density, and increased electrochemical heat production and the SOFC’s maximum temperature. Recknagle et al.^176^ compared the effects of different flow channel modes on the temperature gradient of a planar SOFC, including counterflow, coflow, and crossflow. The maximum temperature differences of the PEN at a voltage of 0.7 V corresponding to the crossflow, coflow, and counterflow were 269 °C, 184 °C, and 267 °C, respectively. The average current densities at a voltage of 0.7 V corresponding to the crossflow, coflow, and counterflow were 0.69, 0.71, and 0.73 A·cm^–2^, respectively. The authors concluded that the coflow SOFC had the smallest temperature gradient, and the average current density could be maintained at a high level. Xu et al.^120^ and Kamvar^177^ also concluded that coflow resulted in a smaller temperature gradient than counterflow via simulations of a single-channel SOFC. Additionally, the electrochemical performance of the coflow SOFC would be better under the same operating conditions owing to the higher temperature of the SOFC under counterflow mode than with coflow and crossflow modes.

**Figure 26:**
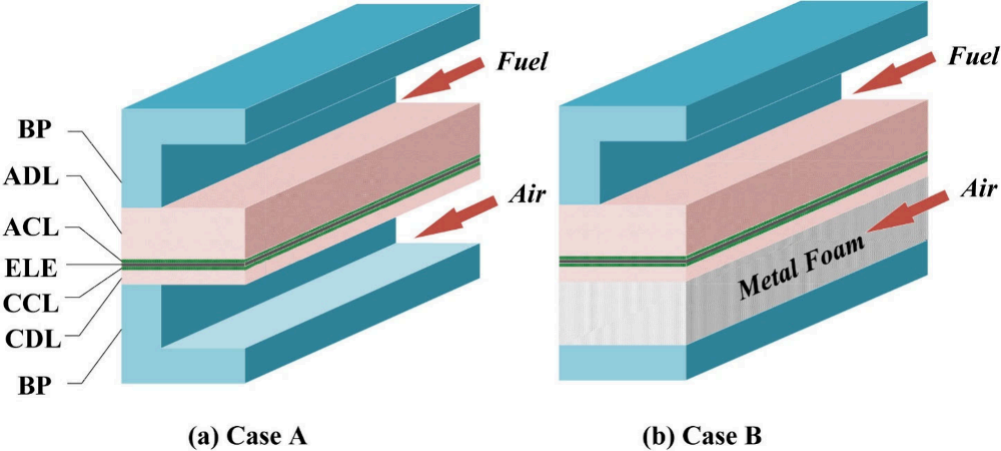
Schematic of (a) the SOFC with a conventional interconnector and (b) the SOFC using metal foam instead of a cathode rib. Reproduced with permission from ref (178). Copyright 2020 Elsevier.

Zhan et al.^178^ designed a novel SOFC cathode flow channel and compared its performance with a traditional SOFC using an MPM. This new channel design employed metal foam as the cathode air distributor and eliminated the cathode rib, as illustrated in Figure 26. The simulation results indicated that the power density of the SOFC with the metal foam distributor could be increased by 13.74% compared to the traditional SOFC when the current density was 5000 A·m^–2^. Moreover, the metal foam could reduce the concentration and ohmic polarization compared to those of the traditional SOFC and was more suitable for thin cells with a cathode gas diffusion layer. The SOFC with metal foam had a more uniform O_2_ concentration and lower temperature distribution than the traditional SOFC.

**Figure 27:**
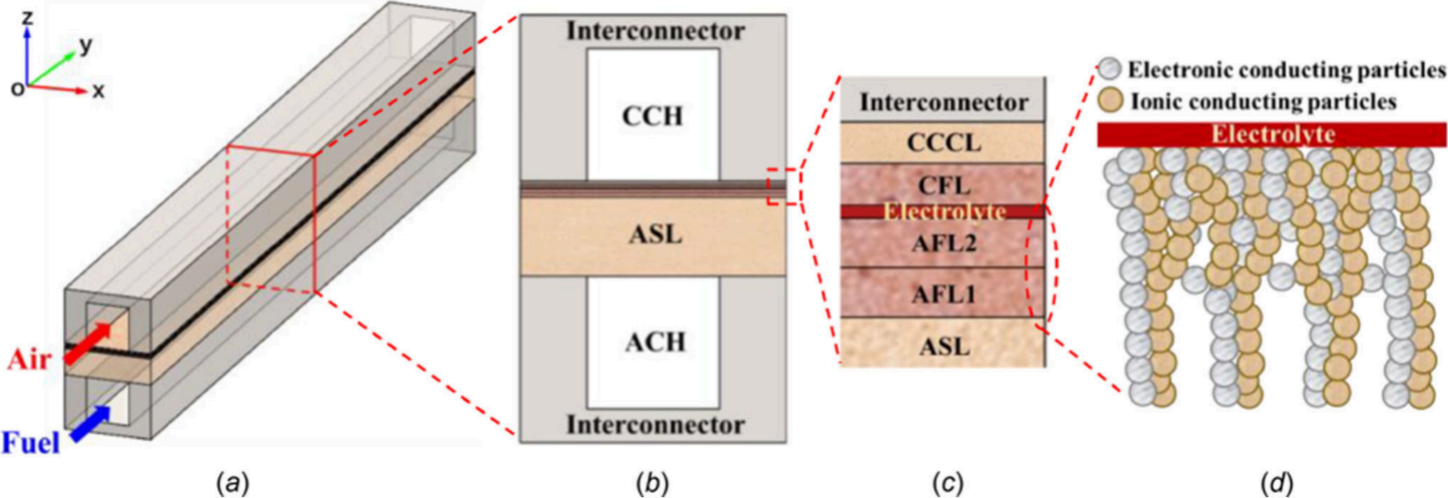
Schematic of (a) the 3D structure of a single-channel SOFC; (b) cross-section of the *x–z* plane; (c) enlarged view of the PEN structure; (d) schematic diagram of an anode with gradient porosity. Reproduced with permission from ref (180). Copyright 2020 ASME.

Our group^179^ first proposed the concept of a gradient electrode (including gradient porosity and particle size) and established the corresponding 1D SOFC model in 2007. The model only considered the electrochemical reactions and mass transfer along the PEN thickness. The authors reported that the gradient electrode structure could significantly enhance gas transport. However, the gradient electrode structure also affected the ohmic and activation overvoltages, especially for thick electrodes. Comprehensive examination revealed that the gradient electrode structure demonstrated significantly improved performance under high current density. At present, gradient electrodes are only effective for small button cells owing to the low current density of large cells or stacks. However, gradient electrodes are expected to work effectively if continuous technological progress yields large cells or stacks that can work under high current density. Fu et al.^180^ designed a novel SOFC structure with gradient anode porosity, as demonstrated in Figure 27, and established the corresponding model to understand the effect of the gradient porosity anode on SOFC performance. Generally speaking, high porosity facilitates gas transport while low porosity contributes to an increased electrochemical reaction active area. Therefore, a reasonable arrangement of the porosity of anode functional layers (AFL) could balance the enhanced mass transfer and increased electrochemical reaction active area, thereby improving SOFC electrochemical performance. The simulation results provided important guidance for the design of gradient porosity anodes. Fu et al.^181,182^ constructed an experimental test workbench for a gradient anode, developed an SOFC numerical model of gradient anode button cells, and confirmed the reliability of the model through experimental data. Based on the above physical model, a neural network was employed to establish the functional relationship between continuous gradient porosity and electrical performance (maximum power density). Finally, a genetic algorithm (GA) was adopted to optimize the continuous gradient porosity distribution. The peak power densities corresponding to optimal porosity design and particle size design were increased by 17.1% and 37.42% compared to initial gradient electrode design, respectively, and the optimal porosity and particle size distributions were reported. Notably, the aforementioned studies only focused on the improvement of cell electrochemical performance and did not consider the effect of gradient electrode design on the mechanical strength of the electrode. High porosity will decrease the electrode’s mechanical strength, leading to electrode failure. In subsequent studies, mechanical strength constraints should be considered when optimizing porosity distribution.

**Figure 28:**
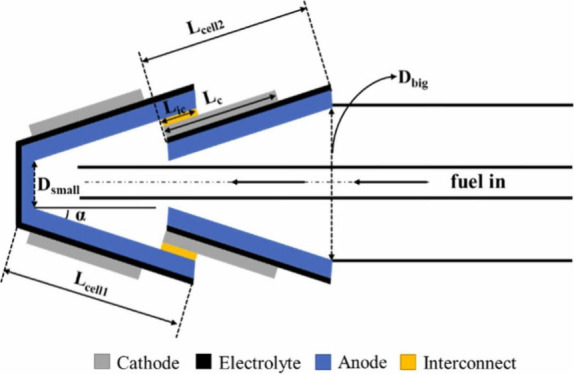
Geometric structure diagram of a cone-shaped tubular segmented SOFC. Reproduced with permission from ref (183). Copyright 2024 Elsevier.

The current long transmission path of tubular SOFCs is problematic; thus, a cone-shaped tubular segmented SOFC was proposed based on a traditional tubular SOFC, with the advantages of a short current path, better thermal shock resistance, thermal cycling ability, etc., making it suitable for lightweight and small device application scenarios. The geometric structure of the cone-shaped tubular segmented SOFC is illustrated in Figure 28. Li et al.^183^ conducted multiphysics simulations of an SOFC with such a structure and investigated the relationship between electrochemical performance and geometric structure. Subsequently, the cell geometry structure was optimized to improve electricity production performance.

**Figure 29:**
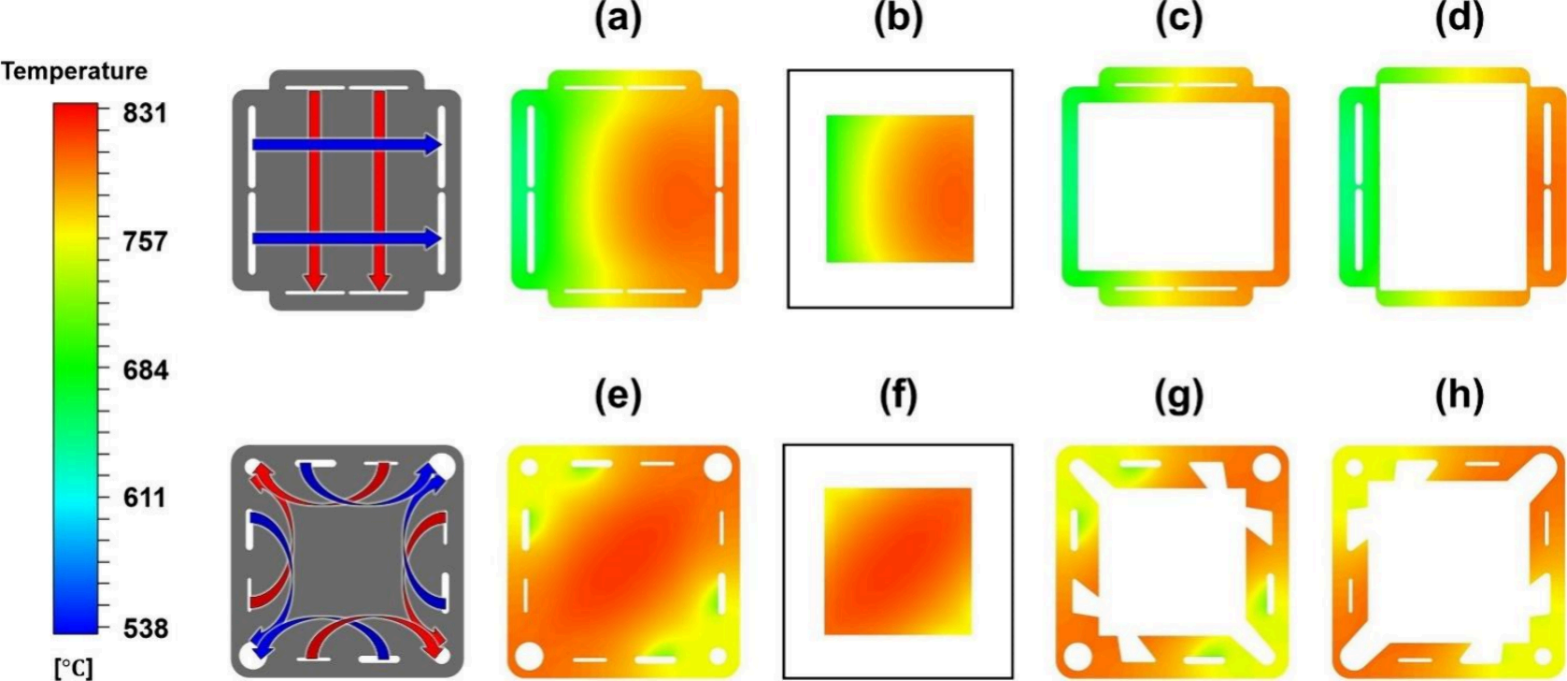
Schematic of conventional and symmetrical intake manifold designs. The blue arrows represent the air flow and the red arrows represent the fuel flow. (a) and (e) illustrate the interconnector temperature distribution in repeating units. (b) and (f) illustrate the cell temperature distribution in repeating units. (c) and (g) illustrate the anode sealant temperature distribution in repeating units. (d) and (h) illustrate the cathode sealant temperature distribution in repeating units. Reproduced with permission from ref (184). Copyright 2021 Elsevier.

Kim et al.^184^ designed a kW-scale commercial stack with a novel interconnector for effective thermal management and constructed an MPM to study the temperature distribution. The inlet pipe was placed at one vertex in the novel interconnector and the outlet pipe was placed at the opposite vertex of the same side, as illustrated in Figure 29. This symmetrical design helped to evenly distribute heat to the four areas of the stack, and the simulation results demonstrated that the novel SOFC stack experienced reduced horizontal heat transfer thermal resistance, leading to a reduction in the in-plane temperature deviation by 35 °C–60 °C. Compared to the SOFC stack with traditional interconnector, the average temperature in the vertical direction of the 30 unit–cell stack increased by 30 °C, and the vertical average temperature difference was reduced by 50 °C.

**Figure 30:**
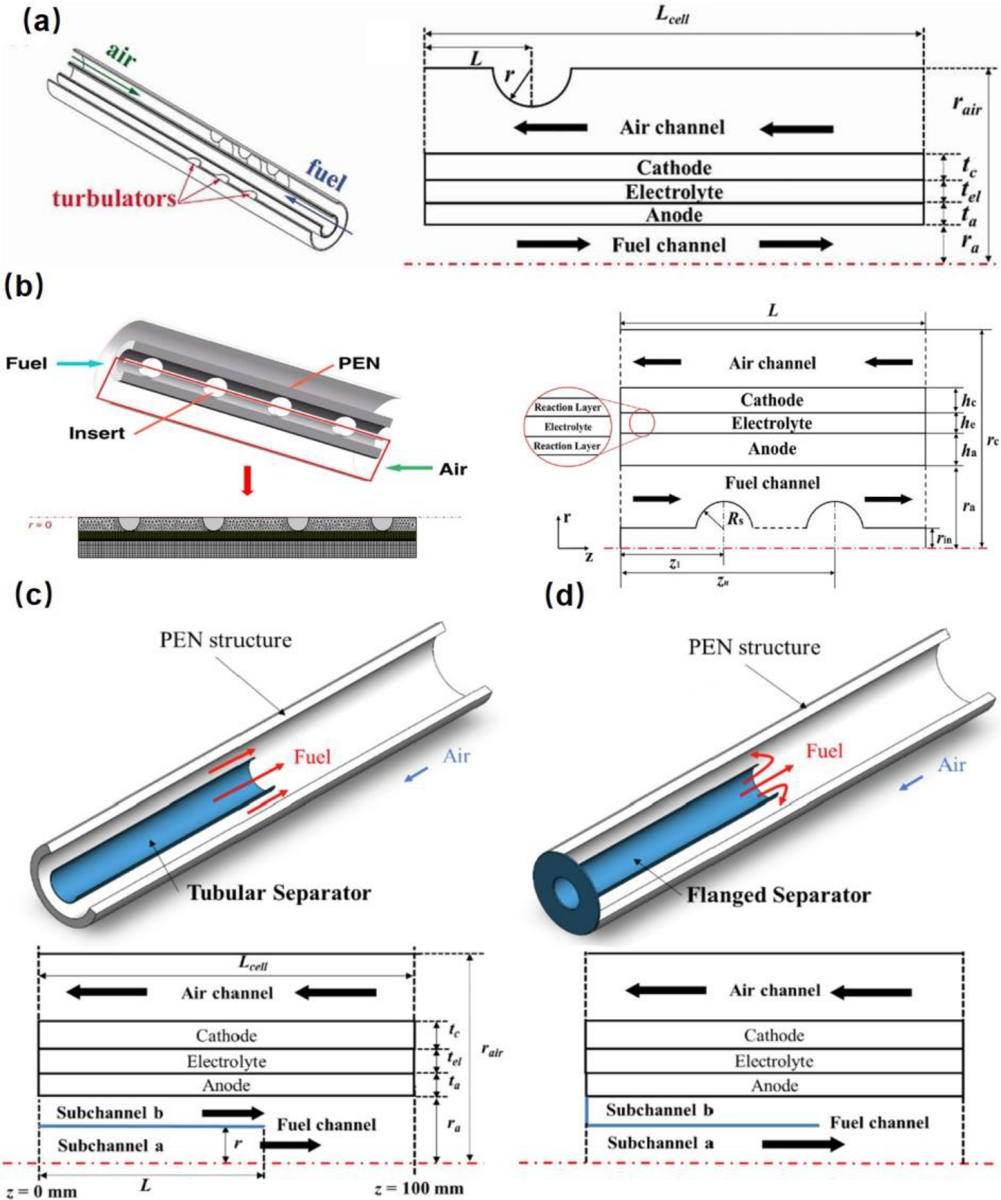
Schematic of circular disturbances in (a) the cathode flow channel^187^ and (b) the anode flow channel;^185^ schematic diagrams of (c) tubular separator and (d) flange separator inserted in the anode flow channel.^186^ Reproduced with permission from refs (185−187). Copyright 2022 Elsevier. Copyright 2022 Wiley. Copyright 2022 Taylor & Francis.

Zhang et al.^185−187^ have extensively investigated the optimization of tubular SOFC flow channels to enhance heat and mass transfer processes. Initially, Zeng et al.^187^ introduced circular disturbances into the cathode flow channel to improve heat transfer, reduce the temperature gradient, and prevent mechanical failure. The specific structure of the circular disturbances is illustrated in Figure 30(a). The cell was fueled by H_2_, and the cathode and inlet gas velocities were 5.0 and 1.5 m·s^–1^, respectively. The numerical results demonstrated that the addition of circular disturbances induced recirculation flow, enhanced the local heat transfer coefficient, and improved heat transfer from the electrode to the gas. The circular disturbances effectively reduced the temperature gradient, narrowing the region where the temperature gradient was >30 °C/cm. Compared with the gas channel without disturbances, the disadvantage of the gas channel with circular disturbances was that the average current density was slightly reduced and the pressure drop was increased. The average current density of the cell with a 2 mm radius turbulator was reduced by 5.2% compared to that without a turbulator. This study contributes to the design of reasonable internal disturbances for controlling temperature gradients. Additionally, Zhao et al.^185^ incorporated circular disturbances into the anode flow channel to enhance anode mass transfer processes, as depicted in Figure 30(b). The simulation results indicated that the circular disturbances amplified the radial secondary flow, facilitating enhanced H_2_ flow into the porous anode while inducing greater H_2_O outflow from the anode, ultimately enhancing electrical performance. Furthermore, the simulation results confirmed that the increased electrical power resulting from the turbulator was sufficient to fully compensate for the additional pump power required. Utilizing four turbulators and a disturbance radius of 3.39 mm, the net power density and CH_4_ conversion were increased by approximately 4.25% and 11.8% compared to microtubular without an insert, respectively. In summary, the electrochemical performance of SOFCs can be effectively improved through the addition of internal disturbances. Furthermore, Hao et al.^186^ proposed tubular and flange separators in the anode flow channel to regulate the generation of electrochemical heat, as illustrated in Figure 30(c). The numerical simulation results demonstrated that both schemes could reduce the cell temperature gradient; however, the flange separator achieved a more uniform temperature distribution than the tubular separator. Compared to a conventional SOFC, the 1.8 mm radius turbulator separator could reduce the temperature gradient from 50 °C/cm to 36 °C/cm with 36% power density loss, while the flanged separator could reduce the temperature gradient from 50 °C/cm to 18.7 °C/cm with 17% power density loss. The influences of separator length and radius on the heat transfer process were also explored. This study provides a novel solution for the thermal management of tubular SOFCs. Additionally, Zhang et al.^188−190^ studied the effects of different inset shapes, including straight rods with circular, trapezoidal, and trumpet-shaped humps and helical rods, in the gas channel on the heat and mass transfer performance of SOFCs using MPMs.

The above content provides a comprehensive review of previous research achievements in optimizing SOFC structures using numerical simulation techniques. To facilitate comparative analysis, the above-mentioned studies are summarized in Table 9, demonstrating that most of the models utilized in structural optimization are 3D or 2D axisymmetric models, primarily because the interconnector can only be adequately represented in a 3D model. Studies have explored the optimization of stack flow channels, single-channel cell interconnector structure, flow mode, tubular SOFC flow channels, and even gradient anodes. These ideas and principles essentially encompass all aspects of SOFC structure optimization. Notably, some novel structural designs are preliminary ideas, and their suitability for practical applications warrants further investigation. The goals of structure optimization are straightforward: (1) enhance mass transfer to reduce concentration polarization and species distribution nonuniformity, and (2) improve heat transfer to reduce temperature gradients. Subsequent studies on structural optimization have primarily focused on these two objectives. Additionally, structural optimization modeling is predominantly performed using commercial software. For example, the two most widely used software packages are COMSOL Multiphysics and Fluent. Based on the above-mentioned studies, the key focus of future SOFC structure optimization should be the development of novel interconnectors or flow channel structures through modeling. These structures should be easy to apply or manufacture and induce substantial positive effects.

**Table 9:** Representative Studies on SOFC Structure Optimization

authors	cell type	dimensions	fuel	structure	target	software	ref
Guo et al.	Planar stack	3D	H_2_	Rectangular rib, cylindrical rib, rhombus rib, straight flow channel, corrugated rib, crisscross rectangular rib	Uniform cathode O_2_ distribution and reduced gas diffusion and current collection path.	COMSOL	(167)
Saied et al.	Planar stack	3D	H_2_	Helical, single-entry serpentine, traditional parallel, modified parallel, double-entry serpentine, triple-entry serpentine	Uniform fuel and O_2_ concentration distributions to improve electrochemical performance.	Fluent	(168)
Fu et al.	Single Channel	3D	H_2_	Novel beam and slot interconnector	Reduce the gas diffusion resistance, shorten the current transfer path, and reduce contact resistance.	COMSOL	(169)
Chen et al.	Single Channel	2D	H_2_	Double-layer interconnect	Increase air flow velocity, strengthen mass transfer, and reduce concentration polarization.	COMSOL	(170)
Zhao et al.	Planar stack	3D	H_2_	Top inlet, center inlet, and buffer chamber inlet ducts	The computational fluid dynamics model was established to analyze flow field uniformity.	Fluent	(171)
Kim et al.	Planar stack	3D	H_2_	Symmetry inlet and outlet manifolds	Effective thermal management was implemented to reduce the temperature gradient.	Fluent	(184)
Khazaee et al.	Single Channel	3D	H_2_	Rectangular, triangular, and trapezoidal flow channels	Comparison of anode H_2_ distribution and electrochemical performance in different flow channels.	COMSOL	(173)
Manglik et al.	Single Channel	3D	H_2_	Rectangular, triangular, and trapezoidal flow channels	Heat transfer characteristics of flow channels with different geometries.	-	(174)
Andersson	Single Channel	3D	H_2_	Rectangular flow channels of different sizes	Comparison of heat and mass transfer performance.	COMSOL	(175)
Recknagle et al.	Planar stack	3D	Prereformed gas	Crossflow, coflow, and counterflow modes	Comparison of temperature fields under different flow modes.	STAR-CD	(176)
Zhan et al.	Single Channel and Planar stack	3D	Syngas	Metal foam replaces cathode rib	Enhanced SOFC performance due to the metal foam distributor.	Fluent	(178)
Fu et al.	Single Channel	3D	H_2_	Anode with gradient porosity	Influence of gradient porosity on electrochemical performance and selection of optimal gradient porosity.	COMSOL	(180−182)
Zeng et al.	Tubular SOFC	2D axisymmetric	H_2_ and syngas	Circular disturbance in flow channel	Enhance heat and mass transfer, reduce the temperature gradient.	COMSOL	(185, 187)
Hao et al.	Tubular SOFC	2D axisymmetric	H_2_	Tubular and flanged separators in the anode flow channel	Heat production was regulated to reduce the temperature gradient.	COMSOL	(186)
Li et al.	Tubular SOFC	2D axisymmetric	H_2_	Cone-shaped tubular segmented SOFC	Optimization of geometric structure to improve electrochemical performance.	COMSOL	(183)

##### 3.2.1.3 Fuel Flexibility

One of the key features of SOFCs is fuel flexibility, a characteristic not commonly found in low-temperature fuel cells. The representative simulation studies of SOFCs fueled by H_2,_ CH_4_, and syngas were summarized and discussed in Section 3.2.1 (Heat and mass transfer mechanism). Similar models have been developed for different fuels. The standard practice involves incorporating the reforming or decomposition reaction sources into the equations governing mass or heat transport. Currently, popular fuels for SOFCs include methanol, ethanol, NH_3_, glycerol, and even solid carbon. Different research groups have investigated the fuel flexibility of SOFCs, evaluating SOFC performance via simulations.

Xu et al.^121^ established an isothermal model of an SOFC fueled by methanol, which aimed to explore the reaction-mass transfer mechanism within the cell and the influence of structural and operating parameters on cell performance. The authors found that the maximum power density could reach 1.2 W·cm^–2^ at an operating temperature of 800 °C, which was significantly higher than that of direct methanol-fueled cell at room temperature (typically <0.1 W cm^–2^). Subsequently, Xu et al.^33^ developed an nonisothermal model of a methanol-fueled SOFC, focusing on thermal effects. The study findings confirmed that the power density of an SOFC could exceed 1.022 W·cm^–2^ at an inlet gas temperature = 800 °C and S/C ratio = 1.0. The power density obtained using the nonisothermal model was lower than that obtained using the isothermal model owing to the inlet fuel of the nonisothermal model containing steam, which resulted in the production of less H_2_ via methanol decomposition. This study further confirmed that cell temperature distribution is substantially influenced by the operating conditions, which was attributed to the electrochemical/chemical reaction heat sources. The difference between the inlet and outlet gas temperatures was approximately 180 °C. The temperature could be reduced by increasing the S/C ratio and air flow rate to ensure long-term stability but at the expense of decreased SOFC efficiency. Based on the nonisothermal model, Xu et al.^120^ investigated the effect of the operating conditions on the temperature gradient.

Dokamaingam et al.^191^ constructed a 1D axial model of a tubular SOFC using methanol as the fuel. This model included a separate reforming chamber, which differed from the model reported by Xu et al.,^33^ and considered possible side reactions during methanol decomposition. The simulation results indicated that the methanol-fueled SOFC with a reforming chamber could achieve autothermal operation, and coflow mode could achieve higher voltage and smoother temperature distribution than counterflow mode.

**Figure 31:**
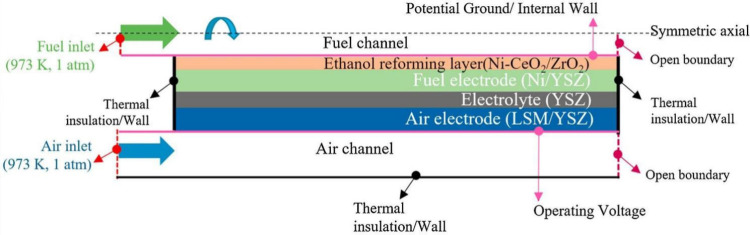
Schematic of 2D tubular SOFC structure with added catalytic functional layer. Reproduced with permission from ref (36). Copyright 2019 Elsevier.

Chen et al.^36^ developed an ethanol-fueled SOFC model with a NiZrO_2_/CeO_2_ functional layer, which provided a suitable site for ethanol reforming. The specific structure is illustrated in Figure 31. The simulation results indicated that the conversion rate of ethanol could reach 90.3% at 700 °C. The current and power densities of the cell were 4385.6 A·m^–2^ and 2631.4 W·m^–2^, respectively, at an operating voltage of 0.6 V. The study findings indicated that adding a reforming layer was an effective way to convert conventional hydrocarbon fuel into H_2_-rich fuel, such as ethanol. Maintaining the steam-to-ethanol ratio >3 was recommended to prevent carbon deposition.

**Figure 32:**
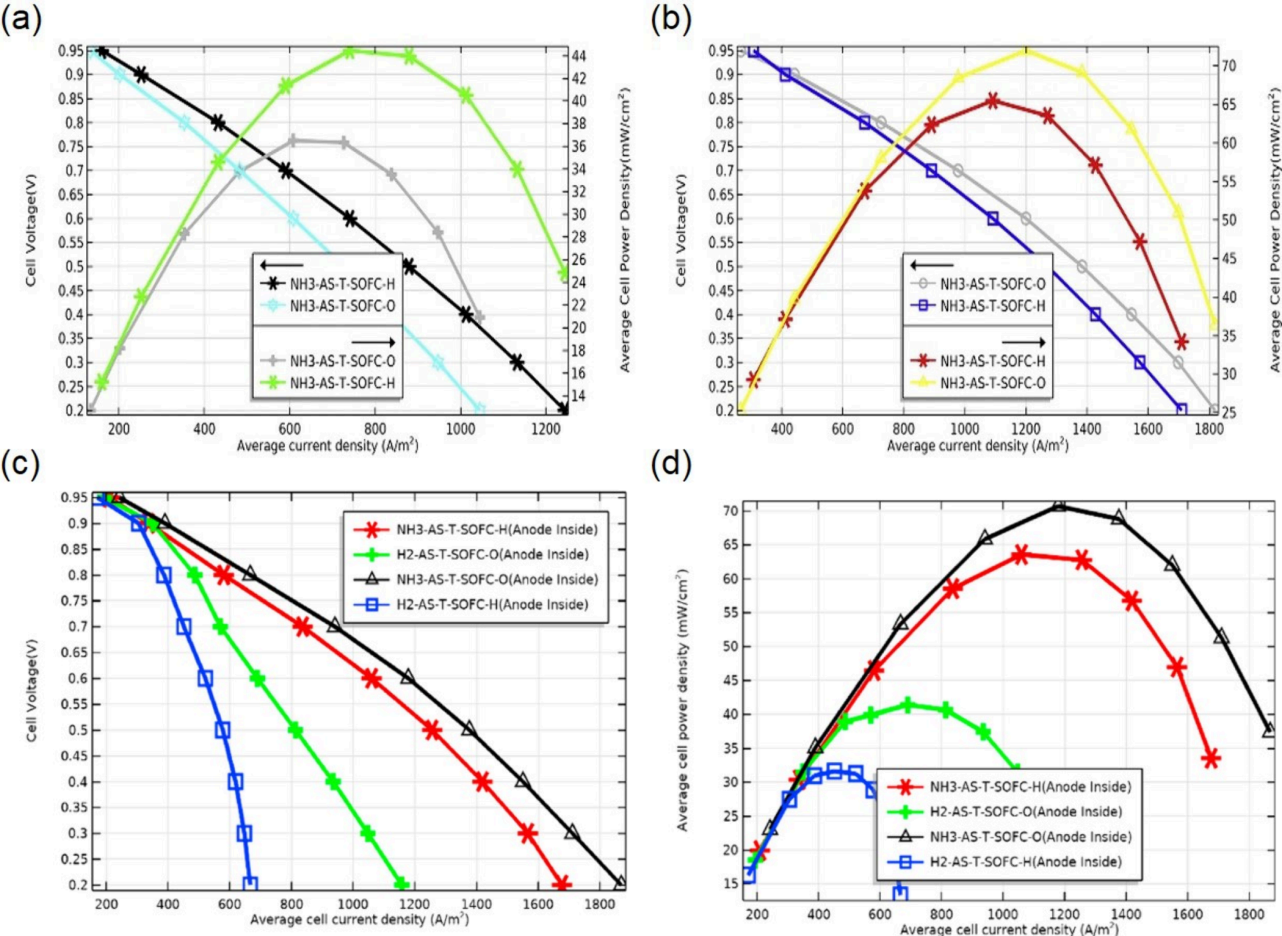
Comparison of *I–V* and power density curves of NH_3_-fed SOFC-O and NH_3_-fed SOFC-H at operating temperatures of (a) 700 °C and (b) 800 °C. Comparison of (c) *I–V* curves and (d) power density curves for four cell types at an operating temperature of 800 °C (AS: anode supported). Reproduced with permission from ref (198). Copyright 2021 Elsevier.

NH_3_ can act as a carrier for H_2_ and used as a fuel for SOFCs. Twenty years ago, researchers employed NH_3_ as the fuel for SOFCs to generate power, reporting good performance.^192^ No NO_*x*_ was detected during SOFC operation, indicating that fueling SOFCs by NH_3_ was feasible. Numerical simulations of NH_3_–SOFCs have rapidly advanced. Ni et al.^193−195^ conducted early simulation work on NH_3_-SOFCs. Ni et al.^194^ developed a PEN cross-section model for an NH_3_-fed SOFC with proton-conducting electrolyte, which considered the mass transfer and electrochemical reactions. The authors found little difference in voltage and power density between H_2_- and NH_3_-fueled SOFCs with electrolyte support structures. This finding was mainly attributed to the extremely high ohmic overvoltage caused by the electrolyte in SOFC-H. For an anode-supported SOFC-H with a thinner electrolyte layer, the output voltage of the NH_3_-fed SOFC was significantly lower than that of the H_2_-fed SOFC. This finding was mainly attributed to NH_3_ decomposition producing N_2_, which diluted the H_2_ partial pressure and led to substantially increased concentration overvoltage. Kalinci^196^ established a similar PEN cross-section model and compared the electrochemical performance of NH_3_-fed SOFC-H and H_2_-fed SOFC-H, obtaining similar results. Ni et al.^193^ further developed a cross-section model of an NH_3_-fed SOFC and compared the electrochemical performance of NH_3_-fed SOFC-O and SOFC-H. Compared with SOFC-O, SOFC-H demonstrated a larger cathode concentration overvoltage, which was mainly attributed the electrochemical reactions in SOFC-H generating H_2_O, which diffused to the cathode, diluting the O_2_ partial pressure and weakening the O_2_ mass transfer process. At an operating temperature of 800 °C, the electrochemical performance of NH_3_-fed SOFC-O was better than that of NH_3_-fed SOFC-H. Ishak et al.^197^ compared the thermodynamic and electrochemical performances of an anode-supported NH_3_-fed SOFC-O and SOFC-H through single-channel SOFC modeling. The authors concluded that the electrical performance of NH_3_-fed SOFC-H was better than that of NH_3_-fed SOFC-O at an operating temperature of 800 °C, while the opposite was true at an operating temperature of 1000 °C. Ni^195^ developed a 2D single-channel NH_3_-fed SOFC MPM and explored the coupling relationship between species concentration, temperature, and current density distributions. Moreover, the authors proposed several measures to reduce temperature differences. Notably, NH_3_ decomposition absorbs a large amount of heat, which can easily cause a local temperature drop in the SOFC, resulting in the appearance of a “cold spot.” Asmar et al.^198^ developed a 3D tubular SOFC MPM and compared the performance of NH_3_-fed SOFC-H, H_2_-fed SOFC-H, and NH_3_-fed SOFC-O. The authors reported that the performance of NH_3_-fed SOFC-H was better than that of NH_3_-fed SOFC-O at an operating temperature of 700 °C, but the opposite was true at an operating temperature of 800 °C, as shown in Figure 32. This finding was mainly attributed to the higher conductivity of the O^2–^ electrolyte than the H^+^ electrolyte. The authors’ conclusions agreed well with those of Ni et al.^193^ and contradicted those of Ni et al.^199^ and Ishak et al.^197^ at and operating temperature of 800 °C. The inconsistency between the conclusions reached in these studies may have been caused by different operating conditions, cell structures, NH_3_ decomposition kinetics, and other factors. At present, NH_3_ decomposition kinetics are described by three equations: in the first case, NH_3_ completely decomposes at temperatures >600 °C,^193^ the other two equations can be found in Table 7. Selection of the appropriate and accurate NH_3_ decomposition kinetics equation is important for the reliability of NH_3_-fed SOFC models. Masashi et al.^141^ tested the NH_3_ decomposition rate on the Ni-YSZ anode and confirmed that the second NH_3_ decomposition kinetic formula in Table 7 better agreed with the experimental data. These findings suggested that the second NH_3_ decomposition kinetics equation can more accurately describe the practical NH_3_ decomposition process than the other two equations. In subsequent modeling, adopting the second NH_3_ decomposition reaction kinetics equation in Table 7 is recommended. The above analysis suggested that the performance differences between SOFC-O and SOFC-H need to be further explored to reach a unified conclusion.

Asmar et al.^198^ reported that NH_3_-fed SOFC performs better than H_2_-fed SOFC, as shown in Figure 32, which seemed to contradict the conclusion of Ni et al.^194^ Asmar et al.^198^ attributed their findings to NH_3_ having a greater volumetric power density than H_2_, leading to the production of higher H_2_ concentrations in the case of complete NH_3_ decomposition. The authors suggested that the contradiction between the two studies was due to their different model settings. In the model by Ni et al.,^194^ the composition of the anode inlet fuel was directly set to 66.7% H_2_ and 33.3% N_2_. Moreover, the model by Ni et al.^194^ focused on mass transfer and electrochemical processes and did not consider gas flow. By contrast, the model by Asmar et al.^198^ considered the gas flow in the flow channel, and the anode inlet gas was pure NH_3_. The H_2_ concentration may have increased due to the decomposition of NH_3_ during the flow process. In short, the reasons for such contradictions need to be further explored. In addition, Asmar et al.^200^ compared the influences of anode channel position, support type, operating temperature, and operating pressure on cell performance. Asmar et al.^200^ attempted to validate the reliability of the NH_3_-fed SOFC model with experimental data. However, the experimental data adopted by Asmar et al.^200^ were obtained by testing H_2_-fed SOFC, not NH_3_-fed SOFC, leading to imperfect model validation results.

Ilbas et al.^201^ established an MPM for a tubular SOFC, reporting that the H_2_-fed cathode-supported SOFC performed better than NH_3_-fed SOFC and H_2_-fed SOFC with anode or electrolyte supports. Further, Ilbas et al.^202^ developed a flat tubular SOFC-O MPM with six fuel channels, demonstrating that the performance of the NH_3_-fed flat tubular SOFC was better than that of the H_2_-fed flat tubular SOFC under the same conditions. The performance of the flat tubular SOFC was better than that of the tubular SOFC with the same active area, channel length, and operating conditions.

In practical SOFC operation, the fuel supply is generally in excess to achieve high power output, with a fuel utilization of <80%. These operating conditions reduce the energy conversion efficiency of SOFCs, requiring recirculation of the anode off-gas. Therefore, the suitable anode off-gas recirculation ratio to enhance the efficiency of SOFC systems needs to be determined. Nemati et al.^140^ established a 3D NH_3_-fueled SOFC MPM and focused on the following three issues: (1) comparing the performance of a direct NH_3_-fueled SOFC with that of a precracked NH_3_-fueled SOFC, with the experimental and simulation results indicating that latter performed better than the former; (2) comparing the cell performance at different anode off-gas recirculation rates, with the results revealing that the fuel savings and power reduction rates were 21%–27% and <1%, respectively, when the anode recirculation rate was in the range of 75%–90% compared to without anode recirculation; and (3) evaluating the Ni nitriding degradation potential under different operating conditions. The Ni nitriding degradation potential *K*_*n*_ is defined by eq 75. When *K*_n_ is greater than the critical Ni nitriding potential *K*_n,cr_, Ni nitriding degradation occurs. The critical Ni nitriding potentials at different temperatures were reported by Nemati et al.^140^ and the authors also observed the ASL via scanning electron microscopy (SEM) after testing for 1000 h, reporting that Ni nitriding occurred when *K*_n_/*K*_n,cr_ > 1. Moreover, the larger the value of *K*_n_/*K*_n,cr_, the more severe the Ni nitriding degradation. The study findings indicated that Ni nitriding could be basically ignored when the fuel utilization was 70%, especially at operating temperatures of 750 °C–850 °C, which is practical for industrial applications.
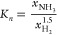
75where *x* is gas molar fraction.

Carbon is a crucial component of fossil fuels, and its efficient utilization is critical for the efficient and clean conversion of fossil fuels. Traditionally, carbon has been utilized to generate electricity by thermal power plants or to produce syngas via the gasification process. However, these conventional methods often lead to pollution and inefficiency. To address this challenge, Ni^41,42,112,203−205^ proposed and numerically evaluated a direct carbon SOFC (DC-SOFC), in which the carbon fuel is gasified in the anode of the SOFC to produce CO for subsequent electrochemical reactions.

Xu et al.^41^ developed a 2D tubular model of a DC-SOFC and focused on electricity and CO coproduction. The authors investigated how different structures and operating parameters affected cell performance and explored the reaction-transfer mechanism inside the DC-SOFC. Despite the large distance between the carbon bed and porous anode, the DC-SOFC demonstrated good performance, highlighting the possibility of large-scale application of DC-SOFCs. Anode-supported DC-SOFCs are more favorable for electricity production but less favorable for CO production compared to electrolyte-supported DC-SOFCs. Owing to the high-temperature requirement of carbon gasification by CO_2_, the operating temperature of DC-SOFCs is usually ≥850 °C, which causes serious catalyst sintering and poor cell durability. To reduce the operating temperature, Xu et al.^205^ proposed using H_2_O instead of CO_2_ for carbon gasification in DC-SOFCs to decrease the operating temperature and maintain good performance. Through numerical simulations and experimental testing, the DC-SOFC with the H_2_O gasification agent outperformed the conventional DC-SOFC with the CO_2_ gasification agent, which was attributed to (1) the rate of carbon gasification by H_2_O was higher than that of CO_2_; and (2) carbon gasification by H_2_O also produced H_2_, which participated in electrochemical oxidation reactions for power generation with a lower activation loss compared to carbon gasification by CO_2_. Further, Xu et al.^42^ developed a nonisothermal model for a tubular DC-SOFC to study the thermal effects and temperature distribution in detail. In another study, Xu et al.^204^ compared the performance of a DC-SOFC with or without an in situ catalyst through numerical simulations. The authors investigated the impacts of operating voltage, anode flow rate, and operating temperature on electricity and syngas production. The simulation results indicated that a suitable catalyst could greatly improve the steam gasification reaction rate, thereby enhancing cell performance. At an operating temperature of 727 °C (1000 K), the DC-SOFC with an in situ catalyst could produce a certain amount of electricity; however, the DC-SOFC without an in situ catalyst could hardly deliver useful electricity. He et al.^112^ investigated the thermal effects and temperature distributions of CO_2_- and H_2_O-assisted DC-SOFCs. The authors explored in detail the influences of various operating parameters (inlet gas flow and temperature, operating voltage) and structural parameters (distance between carbon chamber and anode) on the thermal effects of DC-SOFCs. The study introduced the concept of thermal neutral voltage (TNV), the voltage at which the heat production due to overpotential loss and heat demand for electrolysis reactions are balanced, requiring no external heating or cooling. In summary, the aforementioned studies extensively examined the internal working mechanisms of DC-SOFCs, offering a wealth of insight for optimizing their performance and managing their thermal behavior. Consequently, future studies on DC-SOFCs should prioritize investigating the gasification kinetics and practical applications.

Glycerol is one of the byproducts of biodiesel production, the production of which increases as the production of biodiesel continues to increase.^206^ To efficiently utilize glycerol, Wang et al.^146^ proposed a glycerol-fueled SOFC and evaluated its performance using an MPM. Similar to CH_4_/NH_3_/methanol/ethanol-fed SOFCs, glycerol is introduced to the anode chamber for reforming to produce CO and H_2_. Then, the electrochemical reactions of H_2_ and CO produce electricity. The simulation results indicated that the current density of the glycerol-fed SOFC was 7827 A·m^–2^ and the glycerol conversion was 49% at an operating voltage of 0.6 V and inlet gas temperature of 800 °C. The findings of this study confirmed the feasibility of using glycerol as a fuel for SOFCs.

In general, the high-temperature operation of SOFCs substantially enhances their fuel flexibility, enabling the use of fuels beyond small-molecule gases such as H_2_ and CO. Numerous modeling efforts have explored operational mechanisms and strategies for optimizing SOFC performance with various fuels, laying the foundation for the development of SOFCs suitable for diverse fuel types. Table 10 summarizes the representative simulation studies on SOFC with different fuels. Despite considerable progress in simulating SOFC fuel flexibility, several challenges remain. First, the equations for fuel decomposition and reforming reaction kinetics require further confirmation and verification. Different studies have adopted various reaction kinetics equations for the Boudouard reaction process,^41,205^ necessitating an evaluation of their applicability and reliability. Additionally, experimental data for specific fuel–fed SOFCs should be selected for comparison during model validation. For instance, Chen et al.^36^ only validated the conversion rate and product gas distribution of ethanol decomposition without validating the *I–V* curves of an ethanol–fed SOFC. Ni et al.^194^ only validated the *I–V* curves of a H_2_-fed SOFC without validating the NH_3_-fed SOFC model. This situation arises owing to a lack of suitable experimental data for validating specific fuel–fed SOFCs. To facilitate the further development of models for SOFCs fueled by different fuels, the following issues require further consideration:

- The reliability of reforming or decomposition reaction kinetics equations is crucial for SOFC models using different fuels. Appropriate reaction kinetics equations should be selected based on the operating conditions, catalyst type, and state of fuel reforming or decomposition.
- Model validation should be enhanced, especially for specific fuel models, including validation of electrochemistry and mass/heat transfers. Detailed validation using self-built experimental platforms is preferable.
- Comprehensive comparisons of SOFCs running on different fuels are necessary, followed by discussions of their application scenarios and recommendations for the most suitable fuel for current SOFC development.
- Although the previous works compared the performance of SOFC-O and SOFC-H at the same temperature. However, the comparison of the performance of these two kinds of SOFC is less significant. Because SOFC-O usually works at higher temperatures, while SOFC-H works at lower temperatures.

**Table 10:** Representative Studies on Simulations of SOFCs Fueled by Different Fuels

fuel	authors	cell type	dimension	energy	kinetics	software	main concerns	ref
Methanol	Xu et al.	Tubular	2D	No	MDR, WGSR	COMSOL	Reaction-mass transfer mechanism and parameter optimization.	(121)
	Xu et al.	Tubular	2D	Yes	MDR, WGS	COMSOL	The coupling relationship between temperature distribution, reaction heat, and current density under different operating conditions.	(33)
	Xu et al.	Tubular	2D	Yes	MDR, WGSR	COMSOL	Reduce the temperature gradient by different measures.	(120)
	Dokamaingam et al.	Tubular	1D	Yes	MDR, DME. WGS, MFF, MF, MSR	MATLAB	A 1D model considering the side effects of methanol decomposition was developed. The SOFC structure contained a separate reforming reaction chamber.	(191)
Ethanol	Chen et al.	Tubular	2D	Yes	EDR, MSR, WGS	COMSOL	The SOFC fuel was converted from H_2_ to ethanol by adding a functional layer. The reactions, transfer, temperature distribution, and carbon deposition of ethanol-fed SOFC were studied.	(36)
NH_3_	Ni et al.	Cross-section	2D	No	AD	In-house code	The mass transfer and electrochemical performances of NH_3_-fed SOFC-H, H_2_-fed SOFC-H, and NH_3_-fed SOFC-O were compared.	(193, 194)
	Ni	Single channel	2D	Yes	AD	In-house code	The coupling characteristics of current density, substance concentration distribution, and temperature distribution.	(195)
	Ishak et al.	Single channel	2D	No	AD	MATLAB	Comparison of thermodynamic and electrochemical performances of anode-supported NH_3_-fed SOFC-O and SOFC-H.	(197)
	Asmar et al.	Tubular	3D	No	AD	COMSOL	The performances of four cell types were compared under different parameters and structures: NH_3_-fed SOF-H, H_2_-fed SOF-H, NH_3_-fed SOF-O, and H_2_-fed SOFC-O.	(198)
	Asmar et al.	Tubular	3D	Yes	AD	COMSOL	Comparison of NH_3_-fed SOFC model and experimental results.	(200)
	Ilbas et al.	Tubular	3D	Yes	AD	COMSOL	Performance comparison between NH_3_-fed and H_2_-fed tubular SOFC, anode-, electrolyte-, and cathode-supported SOFCs.	(201)
	Ilbas et al.	Flat tubular	3D	Yes	AD	COMSOL	Performance comparison between NH_3_-fed flat tubular SOFC and H_2_-fed flat tubular SOFC.	(202)
	Nemati et al.	Single channel	3D	Yes	AD	COMSOL	Performance comparison between the direct NH_3_-fueled SOFC and the precracked NH_3_-fueled SOFC. Performance comparison at different anode off-gas recirculation rates. Evaluation of the Ni nitriding degradation potential under different operating conditions.	(140)
Carbon	Xu et al.	Tubular SOFC	2D	No	BR	COMSOL	DC-SOFC was proposed for the coproduction of electricity and CO, and the reaction-mass transfer-flow mechanism was explored.	(41)
	Xu et al.	Button cell	2D	No	BR, WG, WGS	COMSOL	Comparison of H_2_O-supported and CO_2_-supported DC-SOFC and performance optimization of DC-SOFC.	(205)
	Xu et al.	Tubular SOFC	2D	Yes	BR	COMSOL	DC-SOFC thermal effects and temperature distribution were studied in detail.	(42)
	Xu et al.	Button cell	2D	No	BR, WG, WGS	COMSOL	Performance comparison of DC-SOFC with or without an in situ catalyst.	(204)
	He et al.	Tubular SOFC	2D	Yes	BR, WG, WGS	COMSOL	Comparison of thermal effects of CO_2_-assisted DC-SOFC and H_2_O-assisted DC-SOFC and parametric analysis on thermal effects.	(112)
Glycerol	Wang et al.	Tubular SOFC	2D	Yes	GDR, WGS	COMSOL	The reaction-transfer mechanism of an SOFC fueled by glycerol.	(146)

##### 3.2.1.4 Stress Distribution

Stress simulations primarily aim to determine the internal stress distribution to prevent SOFC failure caused by excessive internal stress. SOFCs may suffer from considerable temperature gradients in a high-temperature environment with chemical/electrochemical reactions, resulting in substantial thermal stress. The thermal stress of SOFCs can be modeled by incorporating the solid mechanics module into existing thermochemical models. The following is a brief introduction to thermal stress calculations.

The calculation of thermal stress is based on the theory of linear elasticity. Cell material is considered to be isotropic. In the elastic framework, the strain ε induced by the temperature gradient can be calculated using eq 76, as follows:
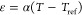
76where α is the thermal expansion coefficient; and *T*_ref_ is the reference temperature. *T*_ref_ is the sintering temperature and usually set as 800 °C (1073 K). Thermal stress occurs at temperatures >800 °C.^207^

The stress σ is equal to the strain multiplied by the elastic modulus *D*, as expressed by (77).
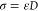
77where *D* is Elastic modulus matrix, which is expressed by eq 78,^208,209^ as follows:
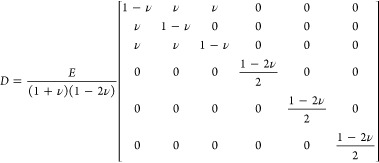
78where *E* is the Young’s modulus and ν is the material’s Poisson ratio. Table 11 presents the mechanical properties of commonly used SOFC materials, including *E*, ν, and α.

**Table 11:** Mechanical Properties of Commonly Used SOFC Materials^210−212^

components	*E* (GPa)	ν (1)	α (10^–6^ K^–1^)
Cathode interconnector	205	0.28	12.3
Cathode support layer	42.7	0.28	12.4
Cathode function layer	60.8	0.3	11.4
Electrolyte	205	0.3	10.3
Anode function layer	81.1	0.3	11.4
Anode support layer	84.2	0.3	12.5
Anode interconnector	205	0.28	12.3

Generally speaking, regions under large stress are most likely to experience microcracks, leading to cracking and fracturing of brittle ceramic materials. To prevent possible component failure, Weibul^213^ proposed the probabilistic failure theory to estimate the failure probability *P* of brittle ceramic materials, as shown in eq 79.
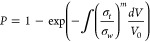
79where σ_t_ is the tensile stress; *m* is the Weibul modulus; σ_w_ is the Weibull strength of the material; *V*_0_ is the reference volume; and *V* is the ceramic material volume.

If the brittle material is subjected to multiaxial stresses, the total *P* can be calculated using eq 80.
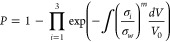
80where *σ*_*i*_ is the *i*-th tensile stress of three principal stresses.

Given that SOFCs comprise multiple components, the overall *P* can be calculated using 81.
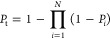
81where *N* is the number of components of the SOFC; and *P*_i_ is the failure probability of the *i*th component.

The values of related parameters involved in the Weibull probability failure theory are summarized in Table 12.

**Table 12:** Values of Related Parameters Involved in the Weibull Probability Failure Theory^214−217^

components	Weibull strength σ_w_ (MPa)	Weibull modulus, *m*	reference volume, *V*_0_ (mm^3^)
Ceramic anode	115.2	6	0.578
Ceramic electrolyte	282	8	0.27
Ceramic cathode	52	4	2.81

**Figure 33:**
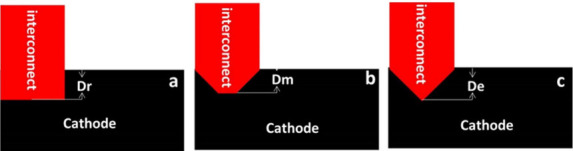
Different interconnector shapes: (a) rectangular; (b) trapezoidal; (c) triangular. Reproduced with permission from ref (220). Copyright 2018 Elsevier.

Based on stress calculations and the probabilistic failure theory, many researchers have calculated the stress distribution in SOFCs to predict their potential failure areas. Li et al.^218^ developed a 3D single-channel MPM of an SOFC running on prereformed fuel and evaluated its thermo-electro-chemo-mechanical behavior. The simulation results indicated that a high maximum temperature of 1311 K and high thermal stress of up to 669 MPa resulted in a *P* value of approximately 1. Despite maximum stress occurring in the electrolyte region, mechanical failure was most likely to occur in the porous ceramic cathode owing to the electrolyte’s greater Weibull strength. Xu et al.^210^ developed a 3D MPM of a single-channel anode-supported SOFC fueled by 30% prereformed natural gas to study thermal stress distribution. The counterflow configuration exhibited a larger temperature gradient and higher maximum thermal stress than the coflow configuration. Mechanical mismatch analysis indicated that stress on both sides of the cell gradually increased under fixed constraints. Similarly, Zeng et al.^219^ developed a 3D single-channel SOFC model to investigate the effect of electrochemical active sites on thermal stress. The authors defined the active area coefficient *Ra* to represent the change in the electrochemical active area, with *Ra* being the multiple increase of the initial electrochemical active area. The simulation results indicated that the first principal stress gradually increased with *Ra*, while a dramatic stress change occurred when *Ra* reached 1.5. This led to large opposite tensile stresses in the electrolyte layer, possibly causing electrolyte cracking. Additionally, Zeng et al.^219^ proposed a strategy to adjust the thickness of the anode functional layer to suppress sharp stress fluctuations caused by the increase in *Ra*. In another study, Zeng et al.^220^ developed a 3D model of a single-channel SOFC fueled by syngas. The authors designed three different interconnector shapes, as illustrated in Figure 33, and explored the influences of interconnector shape and insertion depth on electrochemical performance and stress distribution. The simulation results indicated that, among the three interconnector shapes, the SOFC with the triangular insertion had the best electrochemical performance due reduced active specific resistance at the cathode–electrolyte interface. Additionally, the triangular tips of the interconnector induced the maximum first principle stress at the electrode–electrolyte interface. The maximum first principal stress at the cathode–electrolyte interface increased from approximately 23.7 to 24.1 MPa when the insertion depth of the triangular tip increased from 1 to 10 μm.

**Figure 34:**
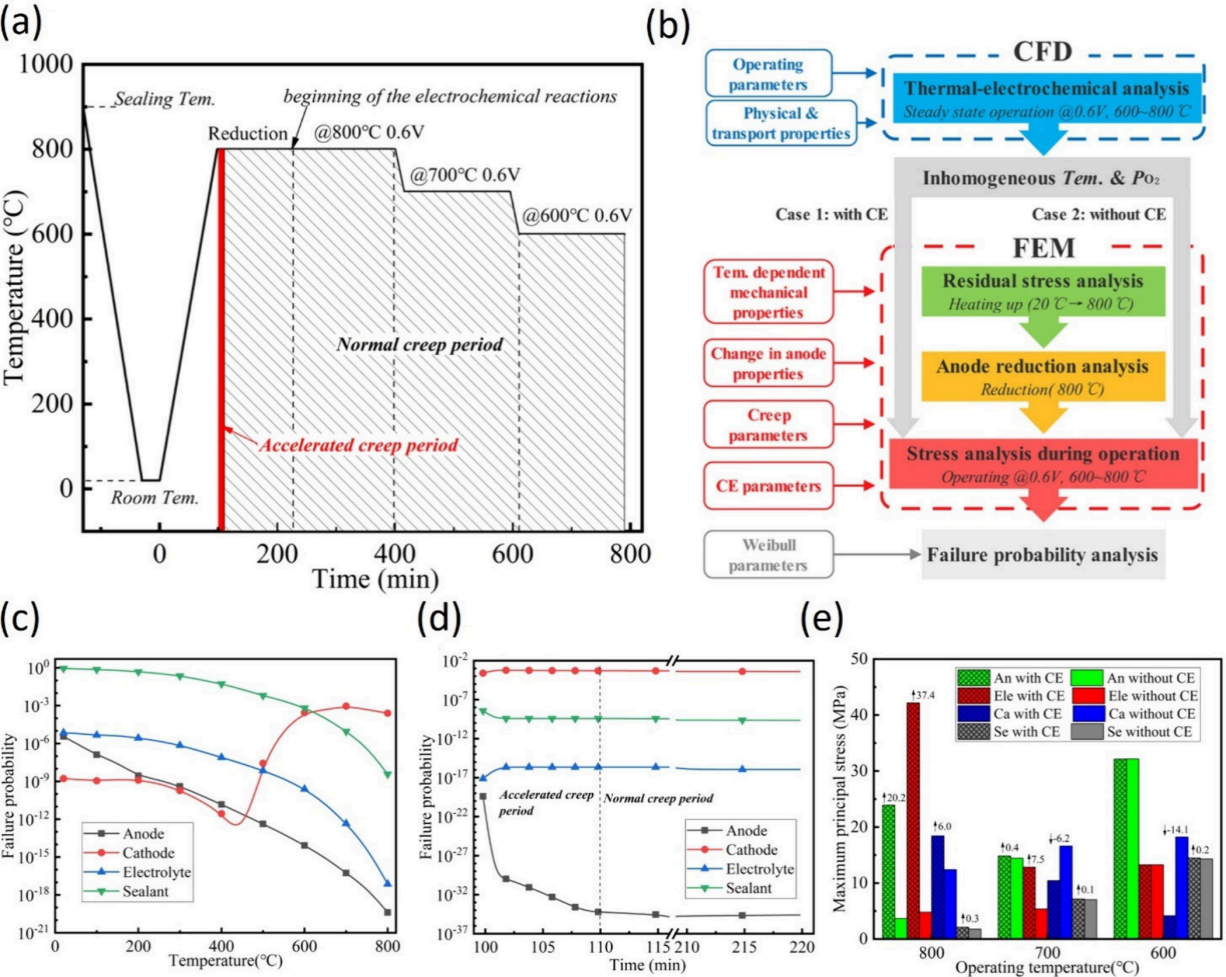
Four stages of SOFC fabrication and operation. (b) SOFC stress simulation analysis process. (c) the failure probability of different components during the heating process. (d) the failure probability of different components during the reduction process. (e) the maximum principal stress of different components at different operating temperatures. Reproduced with permission from ref (221). Copyright 2021 IOP science.

Wu et al.^221^ developed a thermo-electro-chemo-mechanical MPM of a 3D assembled button cell. The study focused on stress and failure risks during cell fabrication and operation processes, which were divided into four stages, namely, fabrication, heating, reduction, and normal operation, as depicted in Figure 34(a). Four factors that could lead to cell failure were considered, namely, thermal stress changes in material properties caused by anode reduction, creep, and chemical expansion, as illustrated in Figure 34(b). The simulation results revealed that the sealant was prone to breaking during the fabrication process, as shown in Figure 34(c), while the cathode was susceptible to failure during the heating-up process, as shown in Figure 34(d). Given that chemical expansion plays an important role in determining the cell failure, the study findings suggested operating the cell at 700 °C to minimize the probability of failure owing to the smallest maximum principal stress occurring at this temperature, as illustrated in Figure 34(e).

**Figure 35:**
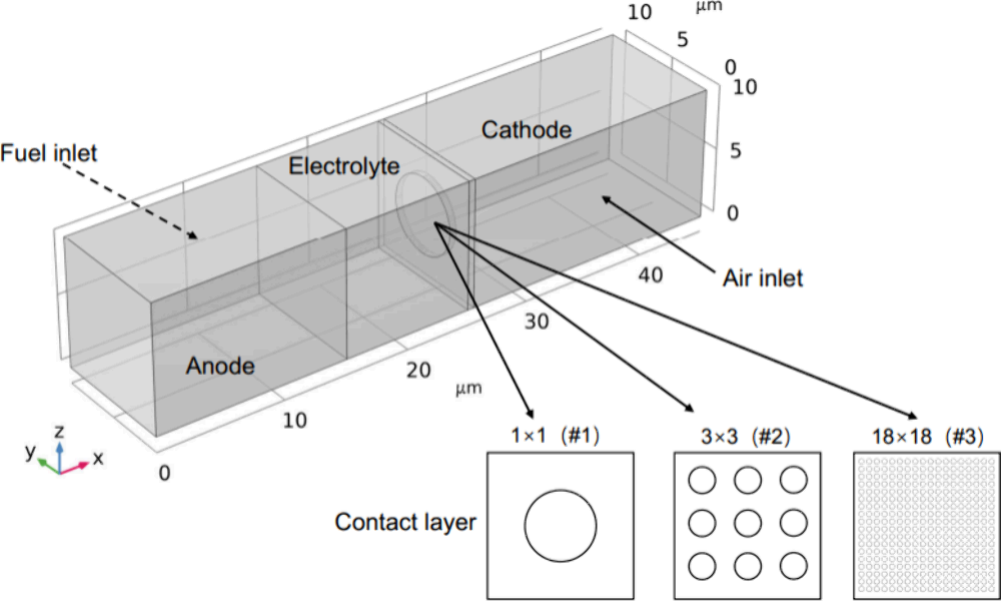
Schematic of geometric structure of an SOFC with three different contact modes of the cathode–electrolyte interface. #1, 1 × 1 active site; #2, 3 × 3 active sites; #3, 18 × 18 active sites. Reproduced with permission from ref.^208^ Copyright 2020 Elsevier.

SOFC interconnectors are generally made of ferritic stainless steel and Ducrolloy.^222^ At high temperatures, chromium (Cr) in the interconnector is vaporized, forming CrO_3_(*g*) and CrO_2_(OH)_2_(g) in dry or wet atmospheres. Cr-containing species in the gas phase will further react with the LSM of the cathode to form the (Cr, Mn)_3_O_4_-type spinal phase, which is deposited in the form of Cr_2_O_3_ at the cathode–electrolyte interface. Cr_2_O_3_ not only reduces O_2_ reduction active sites but also changes the physical properties of the cathode material.^208^ Zhang et al.^223^ developed a model of a 3D single-channel SOFC fueled by syngas to study the thermal stress distribution in the case of cathode Cr poisoning. The authors found that Cr poisoning generated Cr_2_O_3_, which affected the exchange current density, effective conductivity, and physical properties of the cathode, leading to reduced current density, temperature, and stress. Additionally, Zhang et al.^208^ developed a microscopic thermal stress model considering Cr poisoning and temperature gradients. This model divided the cathode–electrolyte interface into three different modes, as illustrated in Figure 35, each with the same total contact area (10.17 μm^2^). The simulation results indicated that electrochemical performance and thermal stress were closely related to the number and area of the contact sites. The first principal stress, third principal stress, and von Mises stress gradually increased with increasing number of contact sites. Moreover, Cr poisoning increased stress and generated disordered stress distribution, reducing the current density and durability of the SOFC.

Sulfur (S) poisoning, similar to Cr poisoning, affects cell performance. S poisoning is mainly caused by SO_2_ in the air, which is first adsorbed on the cathode surface, and then gradually enriched. With increasing SO_2_ partial pressure, S reacts with LSFC grains in the cathode to produce SrSO_4_, La_2_O_2_SO_4_, and MnSO_4_, resulting in decreased conductivity and O_2_ reduction. Xu et al.^224^ examined stress distribution under the influence of S poisoning at the electrolyte–cathode interface. After S poisoning, the first principal stress, third principal stress, and von Mises stress at the contact layer between the cathode and electrolyte decreased due to decreased electrochemical performance. The stress changes at different positions of the electrode–electrolyte interface were not synchronous, potentially leading to delamination of the cathode–electrolyte interface and the appearance of cell cracks.

**Figure 36:**
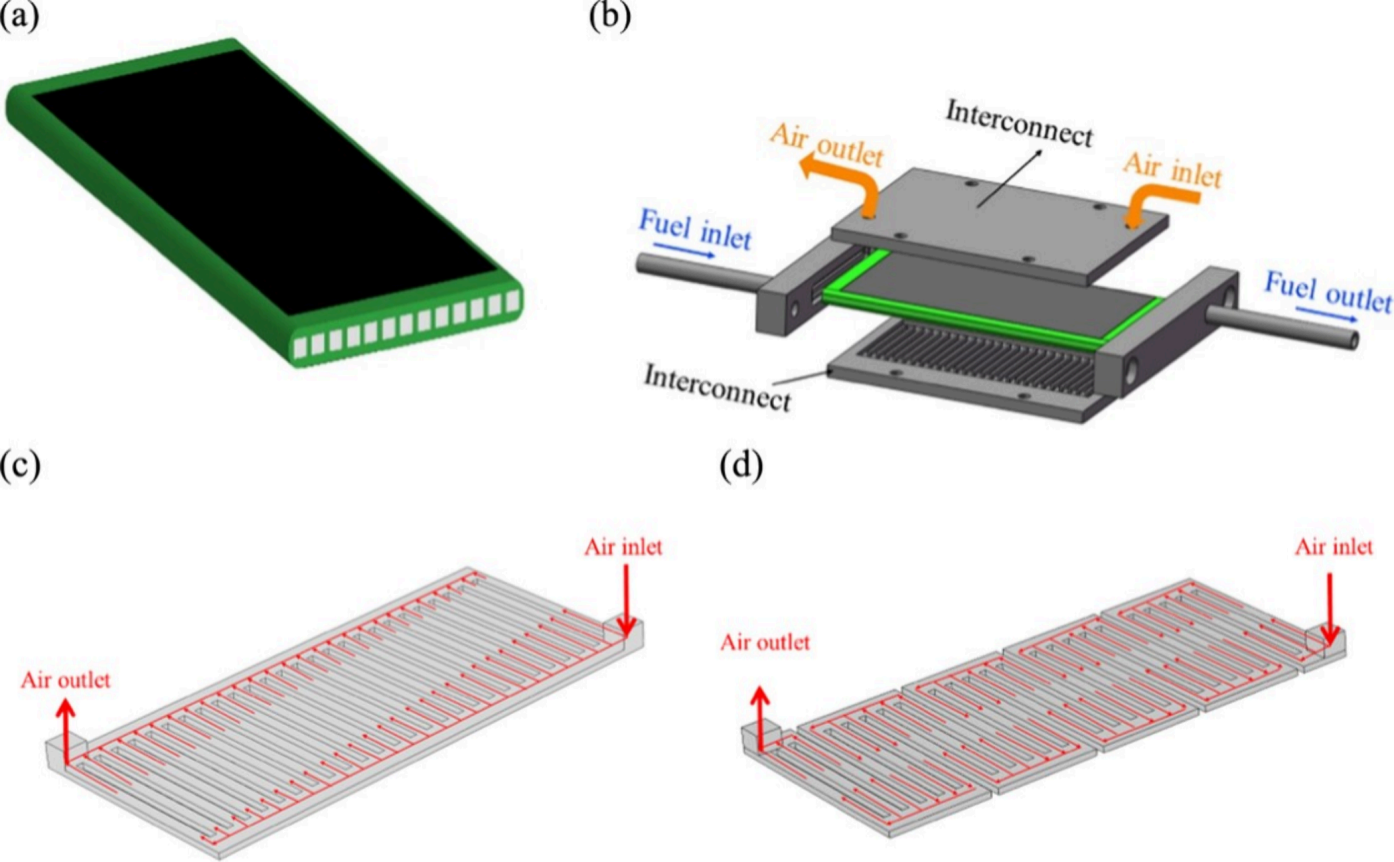
Schematic of a SOFC stack with (a) double-sided cathode channel; (b) stack assembly; (c) Z-type parallel cathode flow channel structure; (d) triple-parallel serpentine cathode flow channel structure. Reproduced with permission from ref (231). Copyright 2020 IOP science.

Guo et al.^225^ created a 3D stack MPM and designed four novel interconnectors to optimize and balance electrochemical and mechanical performance, reporting that the discrete ribs could increase the maximum power density by 12.96% compared with traditional rectangular ribs. The maximum principal stress of the PEN structure exhibited little correlation with interconnector design; however, stress distribution in the SOFC was mainly controlled by interconnector structure. The novel design reduced the PEN failure probability by 28.97% compared with traditional interconnector. Discrete cylindrical and cubic ribs with rounded corners were the preferred designs to balance electrochemical performance and mechanical stability. Guo et al.^226^ developed a 3D MPM for a planar SOFC stack to analyze thermomechanical behavior under different CH_4_-to-steam prereforming ratios. Regardless of the R value, the glass ceramic sealing material was consistently the most vulnerable component, while the cathode remained in a subvulnerable state. The SOFC stack was in a relatively stable working state when the R value was between 0.4 and 0.7. This study provided data support for the safe operation of SOFC stacks using CH_4_ prereforming gas as the fuel. Similarly, Wang et al.^227^ developed a 3D planar SOFC stack thermomechanical model with an external manifold structure, focusing on the compression load effects, seal design, and thermal expansion coefficient mismatch of adjacent components on stress distribution. Their orthogonal experiments confirmed that thermal expansion coefficient mismatch between the cell and interconnector substantially impacted thermal stress, more so than compressive load and seal design. Choudhary et al.^228^ established an MPM of an anode-supported planar SOFC stack fueled by syngas, considering internal reforming of CH_4_. Further, the simulation results indicated that a high air ratio (8.5–14.6), which refers to the ratio of air flow supplied to that required for electrochemical reactions, helped to achieve uniform temperature distribution and reduce thermal stress. An air ratio of 8.5 resulted in relatively high power density and electrical efficiency, with low average PEN temperature and stress, making it the most suitable ratio. Additionally, the crossflow SOFC exhibited lower thermal stress and less carbon deposition than the counterflow SOFC. Yuan et al.^229^ developed a 3D planar SOFC stack model and investigated the influence of cathode air flow rate distribution and flow direction on thermal stress. The results indicated that a change in inlet air flow distribution would affect stack thermal stress, while a change in air flow direction would reduce thermal stress without affecting the power generation of the stack. Zhen et al.^230^ developed a 3D planar SOFC stack model to study the effects of different flow modes (coflow, counterflow, and crossflow) and electrolyte thickness on thermal stress. The results indicated that the maximum first principal stress was highest in the counterflow mode, followed by that in the crossflow mode, and lowest in the coflow mode. These findings aligned with the conclusion that SOFCs with a coflow structure have the lowest temperature gradient, as reported by Recknagle et al.^176^ Decreasing the electrolyte layer thickness increased the power generated by the stack, the temperature, and the temperature gradient, leading to higher corresponding stress levels. Jiang et al.^231^ developed an MPM of a planar SOFC stack with a double-sided cathode structure considering the pressure drop. The authors compared the stress distributions of SOFCs with Z-type parallel and triple-parallel serpentine cathode flow channels. The flow channel structures are illustrated in Figure 36. The results indicated that the difference in maximum first principal stress between these two SOFC types was small under the same operating voltage, with that of the SOFC with a triple-parallel serpentine channel being smaller than that of the SOFC with a Z-parallel channel under the same current density and electrochemical power output. However, the opposite conclusion was reached under the same power output.

The above-mentioned studies comprehensively summarize the main directions of research on SOFC thermal stress distribution. The primary goal of such research is to determine the maximum stress in SOFCs and ensure that it is exceeded by the material strength. Additionally, maintaining consistent thermal expansion rates among SOFC components is crucial to prevent component delamination. Therefore, numerical simulations are commonly employed to determine stress distribution and guide the design of SOFC materials, structures, and operating parameters to prevent damage. Thermal stress in SOFCs primarily arises from temperature gradients, making reaction-transfer MPMs fundamental to stress modeling. Zeng et al.^232^ summarized the thermal management methods adopted to reduce SOFC temperature gradients. Cr and S poisoning and other factors may lead to the reduction of local electrochemical performance, thereby reducing local thermal stress and causing local thermal expansion mismatch. Hence, considering the stress distribution under changing local electrochemical performance is essential.^208,223,224^ Notably, most stress distribution simulation studies are based on 3D stacks or single-channel cells as 2D models have difficulty reflecting the influence of interconnector structure on SOFC stress distribution. Based on the above-mentioned studies, Future research on stress simulations should focus on the following aspects:

- The main factors affecting stress distribution should be identified to simplify the modeling process and quickly obtain the stress distribution to guide SOFC design and operation. It is generally believed that cell ceramic materials undergo elastic, thermal, creep, cathode chemical, and initial strains during the working process.^209,221,233^ Identifying the main stress contributions can not only simplify the model to a certain extent but also control those with the greatest contributions.
- Similarly, local factors that have substantial impact on the stress distribution of SOFCs should be summarized and identified, such as Cr and S poisoning, etc., thereby focusing on those that most contribute to stress.
- Electrochemical performance and strain reduction must be balanced. Controlling thermal stress will reduce the cell temperature and thereby reduce the electrochemical performance of SOFCs. Selecting appropriate operating parameters while maintaining good electrochemical performance and reducing stress is an important concern for SOFC operation.

#### 3.2.2 Development of Transient Models

Transient models of SOFCs have been developed based on steady-state models by incorporating nonsteady terms into the governing equations. Transient models mainly have three application scenarios: (1) considering the dynamic response of SOFCs under variable operating conditions; (2) studying the response during the heating-up process; and (3) studying the SOFC failure mechanism and predicting operational lifetime. To comprehensively understand the current status of transient model research, these three aspects are reviewed and summarized in the following section.

##### 3.2.2.1 Dynamic Response under Variable Operating Conditions

**Figure 37:**
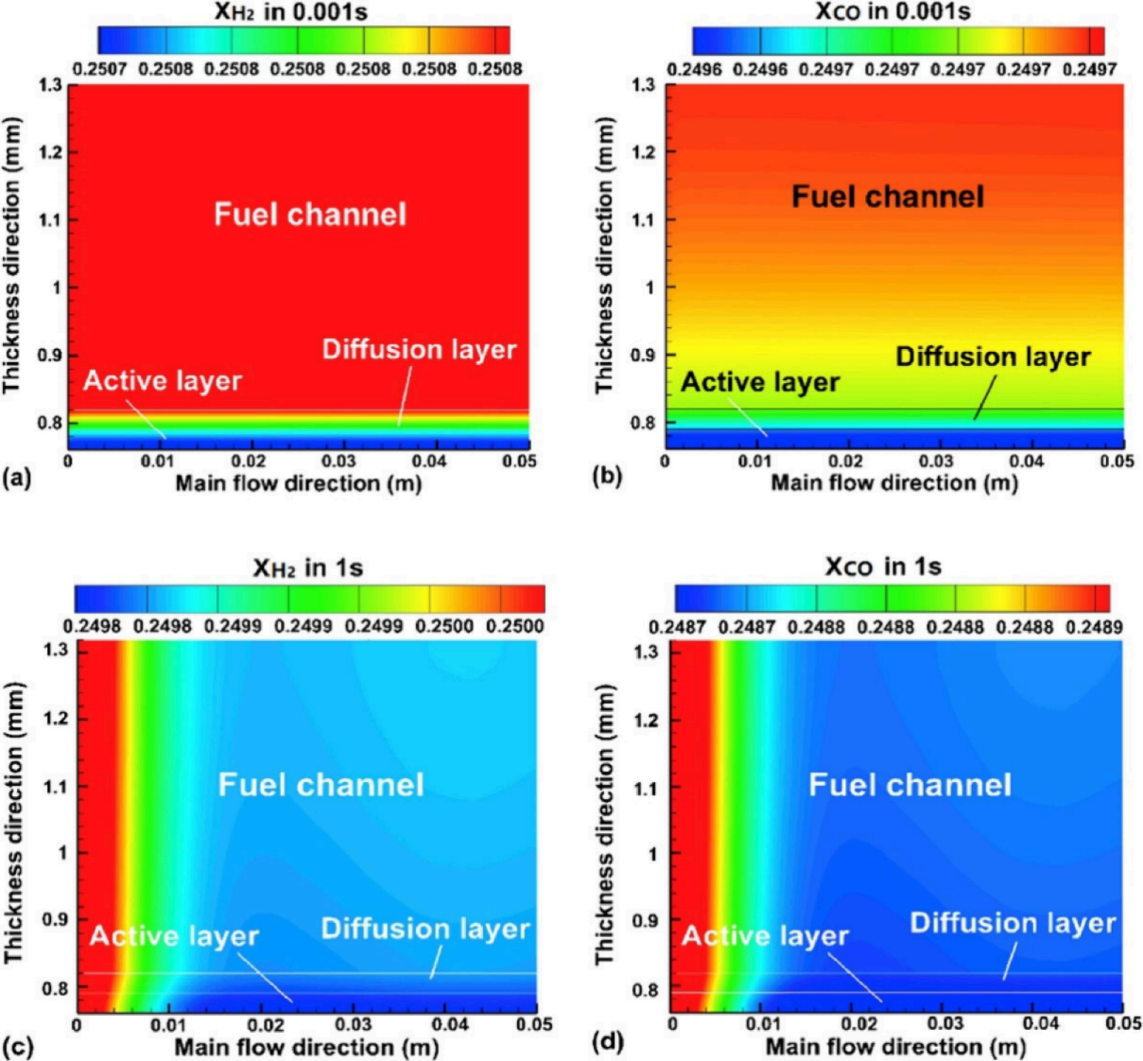
(a) H_2_ concentration distribution in 0.001 s after cell start-up. (b) CO concentration distribution in 0.001 s after cell start-up. (c) H_2_ concentration distribution in 1 s after cell start-up. (d) CO concentration distribution in 1 s after cell start-up. Reproduced with permission from ref (234). Copyright 2019 Elsevier.

Reversible SOFCs can be utilized for power generation and H_2_/CO fuel gas production, offering considerable advantages in managing intermittent energy supply and storage challenges. Consequently, investigating the dynamic response of reversible SOFCs during mode switching is crucial. Yang et al.^234^ developed a transient MPM of a 2D single-channel reversible SOFC to investigate the dynamic responses of an SOFC and solid oxide electrolysis cell (SOEC) during mode switching and start-up operations. The reversible SOFC model was validated using a self-built experimental platform. The study examined the effects of inlet gas temperature, gas composition, and mode switching on current density, gas concentration distribution, and WGS reaction rate. As illustrated in Figure 37, the H_2_ concentration was evenly distributed along the flow channel immediately after cell start-up (0.001 s); however, a gradient H_2_ concentration distribution became clear after 1 s, indicating that H_2_ diffusion gradually developed owing to increased H_2_ consumption. This phenomenon highlighted that the dynamic behavior was dominated by gas diffusion rather than reaction kinetics. Moreover, the dynamic and operating stability performance of the SOFC using syngas as the fuel was found to be inferior to that of the SOFC using H_2_/H_2_O as the fuel. The dynamic response of the electrochemical reactions, reflected by changes in current density, was substantially influenced by operating voltage variations. Additionally, operating under high-temperature conditions could enhance the dynamic response performance of reversible SOFCs.

**Figure 38:**
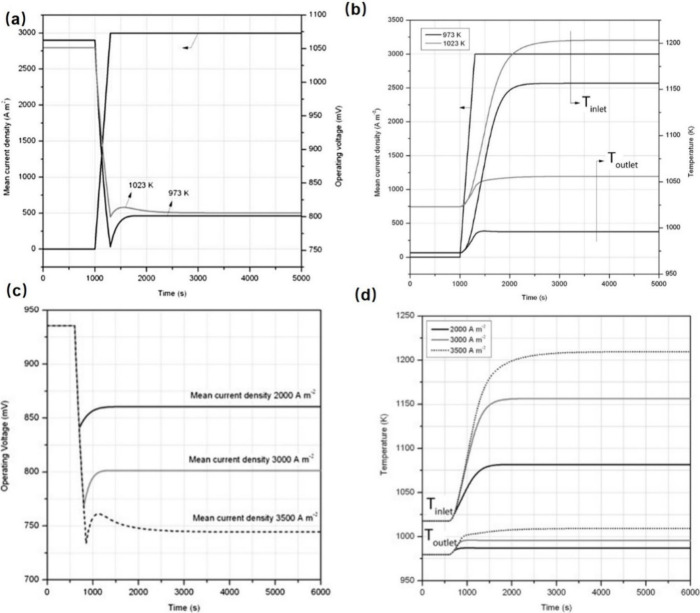
Dynamic responses of the (a) operating voltage and (b) gas temperatures at the inlet and outlet positions during start-up; and the (c) operating voltage and (d) gas temperatures at the inlet and outlet positions during load change. Reproduced with permission from ref (236). Copyright 2015 Elsevier.

Dynamic responses under variable operating conditions are crucial for safe and flexible operation of SOFCs. Tseronis et al.^235^ developed a 2D single-channel transient model of SOFCs and analyzed the dynamic response of current density to step changes in voltage and inlet gas temperature. The simulation results indicated that the current density response caused by a step change in inlet gas temperature was slower than that caused by a step change in operating voltage. This difference was attributed to the slower dynamics of energy transport than of charge transfer. Xenos et al.^236^ developed a 3D single-channel transient model of SOFCs and compared the results with those obtained from a previous model. The authors attributed the differences in model results to variations in model assumptions. Using their developed model, the authors explored the dynamic response of SOFCs to start-up, load, and inlet gas temperature changes. In the start-up scenario, the current density increased from 0 to 3000 A·m^—2^ at a rate of 0.001 A·cm^—2^·s^—1^. The responses of the operating voltage and inlet and outlet gas temperatures during start-up are illustrated in Figure 38(a, b) demonstrating that the operating voltage gradually decreased with lower inlet gas temperatures, corresponding with greater voltage undershoot. This undershoot phenomenon was attributed to the inability of the temperature of the PEN to keep up with the current change. Immediately after a change in current load, the temperature of the PEN was lower than that under steady-state conditions, leading to higher polarization loss and lower operating voltage. The outlet and inlet gas temperatures increased with gradually increasing current density. A lower inlet gas temperature resulted in faster stabilization of the outlet and inlet gas temperatures. Load changes involved the current density increasing from 1000 to 2000 A·m^—2^ or from 3000 to 4000 A·m^—2^. Figure 38(c, d) depict the operating voltage and gas temperature changes at the inlet and outlet positions during load changes.

**Figure 39:**
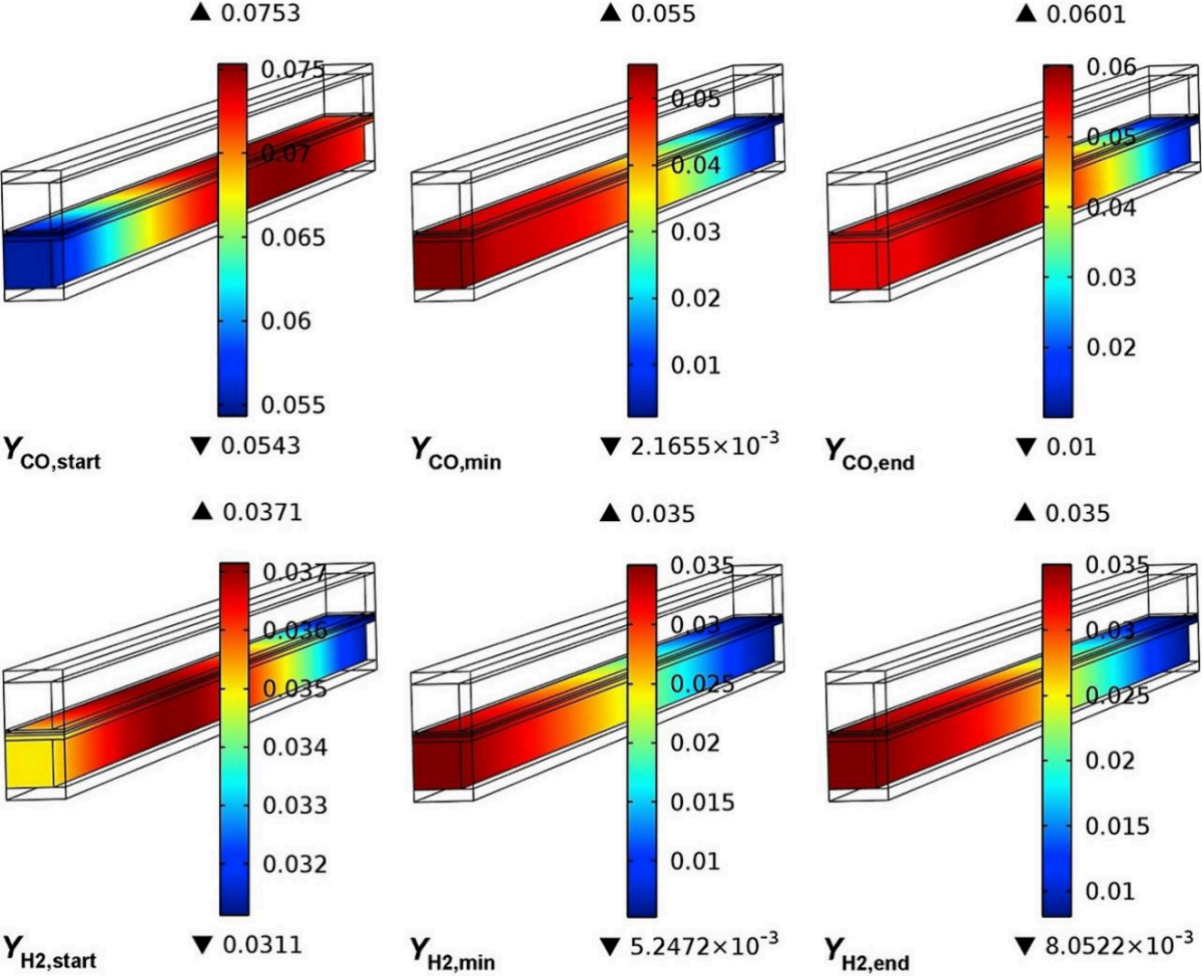
Mass fraction distributions of H_2_ and CO before the load change, the minimum concentration stage, and when stable after the load change. Reproduced with permission from ref (238). Copyright 2017 Elsevier.

Choudhary et al.^237^ established a transient model of a 3D planar SOFC and investigated the dynamic response to changes in current density load. Their explanation for the overshoot phenomenon in operating voltage was consistent with that of Xenos et al.^236^ Additionally, the authors analyzed and compared the dynamic responses of average temperature, fuel utilization, electrical efficiency, average current density, and power density under coflow and counterflow modes. The results indicated that the power density (8.23%) and average current density (8.82%) of the counterflow stack were higher than those of the coflow stack under the same voltage and fuel utilization; however, the electrical efficiency was lower. Compared to the counterflow configuration, the coflow configuration required less time to reach the steady state (2875 s). Nerat^238^ developed a transient MPM of a 3D single-channel SOFC to study the dynamic responses of current density, power density, fuel utilization, and electrical conversion efficiency to step changes in operating voltage. The authors highlighted that excessive voltage variation led to fuel starvation in the anode functional layer, accelerating SOFC performance degradation. Therefore, Nerat^238^ focused on the fuel starvation phenomenon during large voltage changes. The simulation results indicated that fuel starvation occurred when the current density approximately doubled and fuel utilization exceeded 0.85 at the final stable current density. A very thin ASL (approximately 0.1 mm) was more susceptible to fuel starvation with large load changes than a thicker ASL. The fuel concentration distribution was assessed before, during, and after load changes, as illustrated in Figure 39. At the time of overshoot caused by load change, the H_2_ and CO concentrations at the outlet of the SOFC anode were very low, close to 0. Hence, a thicker ASL (approximately 0.5 mm) was recommended to avoid fuel starvation. The thicker ASL increased the mass and heat transfer resistance, leading to a reduced overshoot in fuel utilization and decreased fuel consumption in the anode active layer, thereby mitigating the likelihood of fuel starvation. Unfortunately, this study did not discuss the effect of rib size on the fuel starvation phenomenon, which should be addressed in subsequent research.

Yuan et al.^239^ developed a transient model of a crossflow planar SOFC using syngas as the fuel based on the FlexPDE platform. The simulation results indicated that all parameters, except temperature, could reach a steady state within a similar time frame. Peksen et al.^240^ developed a transient thermomechanical model of a 3D planar SOFC stack and predicted the temperature and stress distribution evolution during the full thermal cycle. This study fully demonstrated the transient thermal behavior of the SOFC stack during the heating-up, operation, and shutdown stages, and the causes of thermomechanical stress during the full thermal cycle were analyzed. Menon et al.^241^ developed 2D and 3D transient models of SOFCs, which were applied to SOFC stacks fueled by prereformed hydrocarbon fuel, and the transient response to step changes in voltage and current was explored. Additionally, the effects of boundary conditions and stack materials on the response time and internal temperature distribution were presented. The simulation results indicated similar response times of the adiabatic and Riemannian boundary conditions to voltage and current load changes. Ho^242^ constructed a 3D transient planar SOFC model to study the responses of temperature, current density, and activation overvoltage to step changes in operating voltage and fuel composition. The study findings indicated that the response time of temperature was larger than that of current density and activation overvoltage to a voltage step change. This conclusion was consistent with that of Choudhary et al.^238^ and Xenos et al.^236^ In our previous report,^243^ a 2D SOFC model was developed to investigate the dynamic characteristics under operating condition changes, including the fuel flow rate, inlet fuel temperature, and fuel composition. Owing to the hysteresis of temperature change, the time taken for the current density to reach stability was generally 10 min later than that required for the inlet gas temperature, while flow rate change induced a difference of only approximately 100 s. This study provided data support and theoretical guidance for dynamic operation and performance control of SOFCs.

Jin et al.^244^ developed a transient model of a 2D single-channel reversible SOFC to investigate the dynamic response of relevant parameters during SOFC/SOEC switching. The results indicated that the distribution of internal parameters, such as H_2_ concentration and charge potential, would be correspondingly reversed when the operation mode was switched. The mass fractions of O_2_ and H_2_ and charge potential could respond quickly without an overshoot phenomenon. However, the mass fraction of steam decreased and overshoot occurred for a short period of time after the SOFC was switched to SOEC. This finding was mainly attributed to the higher molar mass of H_2_O than of H_2_, and the large inertia of H_2_O led to the overshoot. The research performed by Yang et al.^234^ and Jin et al.^244^ demonstrated that the internal parameters reached stability within a few seconds when the SOFC and SOEC were switched, which basically did not affect the normal operation of reversible SOFCs.

The above-mentioned studies fully explored the dynamic response of SOFCs to changes in load, operating conditions, and mode switching. In general, the internal parameter distribution of SOFCs can respond quickly and reach a new steady state, except for temperature distribution. Owing to its large inertia, temperature cannot quickly follow the changes in other parameters, resulting in the overshoot or undershoot phenomenon. Notably, large operating condition or load changes will extend some parameters beyond the normal working range (such as the phenomena of fuel starvation, overheating, and cooling of the cell), making the SOFC operate outside of the safe operating range. This point can be avoided through numerical simulations. The above-mentioned studies indicate that dynamic simulations should be linked with actual operation in follow-up research as not all operating conditions or load changes can be described by step functions. For example, changes in inlet gas temperature, inlet gas flow, and other operating parameters are not instantaneous but require a certain action time. Considering the dynamic response under actual operating conditions and ensuring that SOFCs are always maintained within the safe operating range are worthy of further research.

In addition, the validation process of dynamic models requires strengthening. Currently, most simulation studies focus on dynamic characteristics and only validate steady-state *I*–*V* curves. Few studies compare the dynamic characteristics obtained experimentally with those obtained from simulations. Experimental testing of cell dynamic characteristics requires considering practical issues such as the switching operating conditions, long-term data recording, high cost, and performance fluctuations. Therefore, few studies have considered experimental validation of dynamic characteristics. The next step in dynamic characteristics research is to overcome the difficulties of experimental validation as much as possible and confirm the reliability of simulation data.

##### 3.2.2.2 Preheating Studies

Preheating studies aim to explore the working mechanism of SOFCs from the beginning of the heating process to reaching the electrochemical reaction temperature. Heating start-up is mainly achieved through direct heating by a high-temperature electric furnace and heating by high-temperature gas. The electric heating method primarily utilizes the radiant heat from the inner wall of the electric furnace to heat the cell, with thermal conduction mainly responsible for heat transfer within the cell. These different heating methods lead to a lag in temperature transfer, resulting in the formation of a large temperature gradient between the inside and surface of the cell.^245^ Notably, the cell will suffer from high thermal stress^246,247^ if the heating rate and furnace temperature are not reasonably controlled, thereby damaging its structure.^248^ High-temperature gas heating, which involves heating the SOFC by introducing heating gas into the flow channel, has advantages such as low cost, simplicity, and ease of operation, leading to its wide adoption.^249−251^ The heating process greatly impacts SOFC performance. The key to the preheating process is utilizing an external heat source to heat the cell safely and quickly to the starting temperature. Few factors can be controlled when heating by a high-temperature electric furnace, mainly the heating rate, final temperature of the cell, and position in the furnace, making this approach simple to operate but difficult to optimize. In terms of the high-temperature gas heating approach, factors such as the heating gas temperature, flow state, and size/structure of the flow channel can affect the preheating process. Therefore, research on optimizing the heating-up process has emerged based on the high-temperature gas heating approach. Since such experimental work is challenging, numerical simulations have become the primary tool for studying this process.

During the preheating process, the cell temperature has not yet reached the required temperature for chemical/electrochemical reactions; therefore, such reactions are not considered in the SOFC preheating model. Only the flow and heat transfer processes are considered. Generally, the preheating time and temperature gradient are employed to evaluate the performance of the heating start-up process. Therefore, most simulation work focuses on the optimization of these two points. The following studies summarize previous simulation work on the SOFC preheating process.

To study the heating-up and start-up processes of SOFCs, Kim et al.^252^ developed a 1D direct internal reforming SOFC transient model along the channel direction. The SOFC was heated by air with constant high temperature in the cathode channel. The simulation results indicated that a large temperature gradient could be avoided by adjusting the air flow rate and temperature; however, the heating time would also be increased. The heating time and temperature gradient could be balanced through the flow rate and inlet gas temperature.

Chen et al.^253^ investigated the process of heating an SOFC using an external CH_4_ burner to provide heat power. The inlet air flow rate was adjusted to maintain a constant difference between the inlet gas temperature and the cell’s minimum temperature. The authors compared the temperature distribution, effective maximum temperature gradient, and heating time under different heating powers and flow modes using a 2D single-channel SOFC transient heating model. The results indicated that the heating time (approximately 1439 s) of the single-channel heating mode was too long for practical applications. Moreover, the heating time under counterflow mode and the total power required were 25% and 20% less than those under coflow mode with fixed burner power, respectively. However, the mean temperature gradient was 17% higher under counterflow mode than under coflow mode.

**Figure 40:**
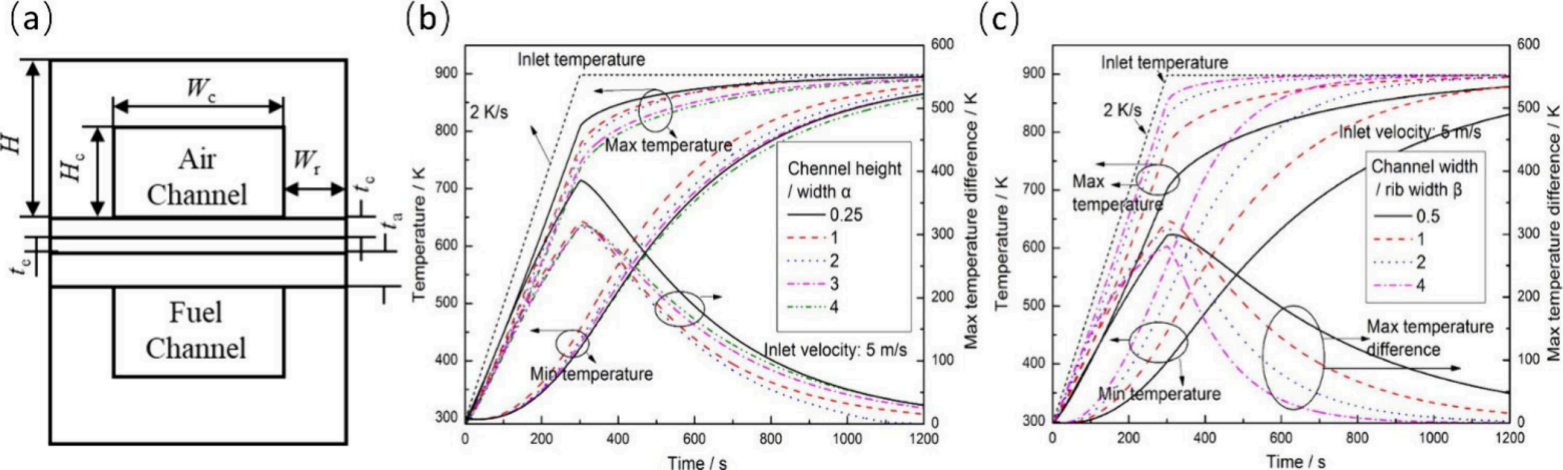
(a) Cross-section of a single-channel cell. The maximum temperature, minimum temperature, and maximum temperature difference in the SOFC unit with time for different (b) channel height/width (α) and (c) channel width/rib width (β) values. Reproduced with permission from ref (254). Copyright 2019 AIP.

Zheng et al.^254^ developed a 3D single-channel SOFC transient heating-up model. The SOFC was heated from 273.15 to 898.15 K by counterflow heating with cathode and anode channels. The cathode and anode channels were fueled by hot air and hot fuel gas, respectively, although the fuel gas composition was not specified. The gas temperature was increased from room temperature 298.15 K and to 898.15 K at a rate of 2 K·s^–1^ and then fixed at 898.15 K. The heating time was defined as the time required for the minimum electrolyte temperature to increase from room temperature to 95% of the target temperature. The results indicated that the bipolar plate volume determined the temperature rise rate and heating-up time, as it absorbed the most heat within the cell. Moreover, the effect of bipolar plate geometry (channel width-to-height and channel width-to-rib width ratios: *α = W*_c_/*H*_c_ and β = *W*_c_/*W*_r_) on the heating-up time and temperature distribution was investigated in detail, as shown in Figure 40(b, c). Shen^255^ established a similar 2D high-temperature gas preheating transient model to explore the distribution of the maximum temperature gradient and mechanism responsible for the formation of the abnormally high-temperature gradient at the inlet. The simulation results indicated that the maximum temperature gradient appeared on the electrode surface at the inlet. The abnormal temperature gradient at the inlet was attributed to the uniform average velocity and temperature, which strengthened heat transfer between the gas and cell. Prolonging the length of the inlet section could fully develop gas velocity and reduce the maximum temperature gradient.

Colpan et al.^256^ established a 3D single-channel heating-up and start-up transient model for a direct internal reforming SOFC to study the heating-up and start-up behaviors in coflow and counterflow modes. Notably, the model in the start-up period included chemical/electrochemical reactions. The model results indicated that the failure probability of the SOFC with coflow mode was 0.068%, while that of the SOFC with counterflow mode was 0.078% during the entire heating-up and start-up process.

**Figure 41:**
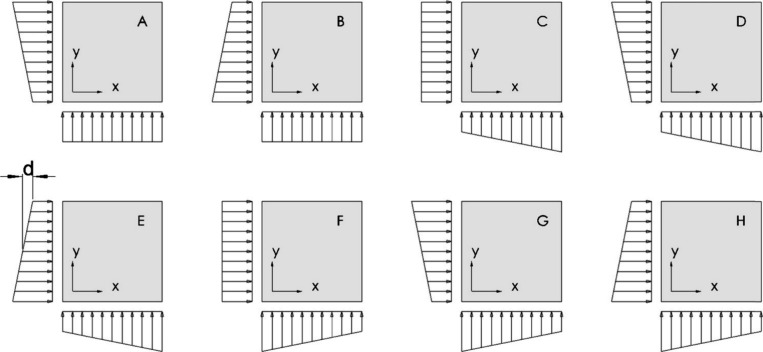
Schematic of different flow patterns. Reproduced with permission from ref (259). Copyright 2016 Elsevier.

Petruzzi et al.^257^ developed a 3D stack transient model of SOFC preheating, start-up, and operation processes, focusing on heat transfer behavior. The authors concluded that the influence of heat radiation should be considered in the SOFC heating process. Increasing the gas mass flow rate and reducing the inlet temperature could effectively improve the heating effect. Selimovic et al.^258^ conducted numerical simulations to compare the temperature field and stress distributions of cells under coflow, counterflow, and crossflow modes. The authors also compared the effects of ceramic and metal interconnectors on the cell heating process. The results indicated that the maximum stress existed at the contact surface between the electrolyte and anode during the heating process. The metal interconnector could substantially improve the SOFC temperature distribution and reduce thermal stress. Liu et al.^259^ developed a transient preheating model for a 3D crossflow SOFC stack and compared the effects of different flow patterns and nonuniform deviations on the maximum temperature gradient and preheating time. The different flow patterns (types A–H) are illustrated in Figure 41. The simulation results indicated that nonuniform flow patterns substantially impacted the maximum temperature gradient, with the fuel side exhibiting more pronounced effects than the air side. Inlet flow type C was the best pattern among the tested flow patterns. The influence of the nonuniform inlet gas flow rate on the preheating time could not be ignored, and the degree of influence increased with increasing nonuniform deviation. This finding was attributed to the temperature distribution varying greatly under different flow patterns with large nonuniform deviations. The heating time was defined as the time required when the difference between the maximum and minimum temperatures was 2 K. The study findings indicated that the nonuniform deviations of the gas flow rate greatly influence the heating time.

*K*_i_ et al.^245^ compared the heating effects of a high-temperature electric furnace and high-temperature gas on a 1 kW planar SOFC stack via numerical simulations. The results revealed that electric furnace and high-temperature gas heating required similar net heating energy to achieve a uniform stack temperature within preset requirements. However, the heating gas temperature remained high after passing through the flow channel. Without recycling the energy carried by the heating gas, the energy utilization efficiency of the high-temperature gas heating method was lower than that of the electric furnace heating method. Amin et al.^260^ conducted experimental and numerical studies on the temperature rise process and temperature distribution of a microtubular SOFC under gas heating, electromagnetic induction heating, hybrid sequential gas heating and induction heating, and hybrid gas and induction heating, with corresponding preheating times of 95, 31, 49, and 20, respectively.

Generally speaking, 3D models consider the rib structure and can obtain accurate temperature distributions and heating times. However, the obvious disadvantage is that the solving time is increased compared to lower dimension models, especially for the 3D stack model. By contrast, 2D models do not consider the influence of rib width on the temperature distribution during the heating process, which can lead to overestimations of the cell temperature under the same heating power. Moreover, 1D models do not consider the rib width and also cannot provide the temperature distribution in the direction of the vertical flow channel. However, 1D models have a short solving time and can obtain results quickly to guide practical operations. In fact, reducing the dimensionality of the model is a simplification that ignores the rib structure. During the practical design or optimization process, the right model dimension should be selected based on the trade-off between model accuracy and solution time.

The above discussion provides a detailed summary of SOFC preheating simulation studies using high-temperature gas. Generally, models for this process are simpler than classical MPMs, as they primarily focus on flow and heat transfer. Some studies have also considered gas mass transfer within porous electrodes, as seen in ref (260). The primary objective of gas heating is to minimize the heating time while maintaining a safe temperature gradient. Previous simulation studies have explored the impact of parameters such as gas temperature rise rate,^261^ gas flow rate,^252,257,261^ flow modes,^253,256,258^ flow channel structure,^254,259^ and other structural and operational parameters on the heating process, leading to reasonable optimization of these parameters. Subsequent research on the preheating process should consider the following points:

- Comprehensive optimization of operating parameters affecting the preheating process should be conducted to reduce preheating time while meeting temperature gradient requirements. Summarizing the optimal operating parameters would provide guidance for actual preheating processes.

##### 3.2.2.3 Performance Degradation Studies and Lifespan Prediction

Studies on performance degradation and lifespan prediction mainly focus on the effects of SOFC degradation mechanisms and the operating lifetime in the presence of degradation factors. In terms of the severity of SOFC performance degradation, failure can be divided into soft and hard failure. Hard failure implies that the SOFC will immediately stop working when it suffers damage, or its power generation performance will degrade rapidly. By contrast, soft failure implies that the cell performance can be maintained within an acceptable range or it will degrade slowly rate when the SOFC suffers damage. Hard failure mainly includes cracking of electrolytes, which is generally caused by mechanical stress, such as thermal stress, residual stress, thermal creep, etc., and delamination, which is mainly caused by excessive gas pressure at the electrode–electrolyte interface and mismatch of the coefficients of thermal expansion between the electrode and electrolyte. The solution to hard failure is mainly to improve the level of cell/stack manufacturing processes and reduce thermal stress, as discussed in Section 3.2.1 (Stress distribution). Soft failure generally includes microstructural changes, oxidation, slow increase in impedance, phase transformations, etc. The solution to soft failure generally involves identifying the factors that cause damage and cell performance degradation to restrain their impact on cell performance by optimizing the operating conditions or cell structure. The specific factors that cause soft failure can appear in each component of the cell, including the anode, cathode, electrolyte, and interconnector. The specific factors that cause soft anode failure include carbon deposition,^262,263^ particle coarsening or migration,^264,265^ and S poisoning,^266,267^ while cathode soft failure is mainly attributed to Cr or Zr oxidation.^268,269^ The electrolyte soft failure is mainly due to the reduction of YSZ intrinsic conductivity.^270^ The soft failure of the interconnector is mainly caused by a thin oxide layer on the surface that reduces electronic conductivity.^271^ Yang et al.^5^ published a detailed summary of SOFC degradation issues, their main mechanisms, and antiattenuation measures. Moreover, the authors summarized simulation studies on SOFC failure from microscopic, mesoscopic, and macroscopic perspectives. The main SOFC soft failure mechanisms and suppression measures are summarized in Table 13.

**Table 13:** SOFC Soft Failure Mechanisms and Suppression Measures

material degradation		
component	mechanism	measure
Cathode	• Cr poisoning: Cr oxides generated in the cathode cover the active sites of electrochemical reactions, hindering gas adsorption and diffusion and increasing ohmic resistance.	• Cr_2_O_3_ in the cathode and interconnector can be inhibited by adding an antioxidant layer.
	• Increase in ohmic resistance: The LSM cathode reacts with the YSZ electrolyte to form an insulating oxide that covers the reactive sites, resulting in an increase in ohmic resistance.	• Add a barrier layer between the LSM cathode and YSZ electrolyte.
Electrolyte	• Reduction in YSZ intrinsic conductivity.	• YSZ conductivity obviously decreases for high-temperature SOFCs (>1000 °C), but less substantially for moderate-temperature SOFCs (600 °C–800 °C).
Interconnect	• Decrease in electrical conductivity caused by the thin oxide layer formed on the surface. For stainless steel materials, the oxide layer is mainly Cr_2_O_3._	• Add an antioxidant layer.
Anode	• Carbon deposition.	• Carbon decomposition and Ni particle coarsening are inherent failure mechanisms, which can be suppressed by operation and structure optimization.
	• Ni particle coarsening.	• Gas cleaning for impurity gas poisoning.
	• Impurity gas poisoning.	

Microscopic SOFC failure simulation studies primarily focus on elemental adsorption/desorption, thermodynamic energy, energy barrier kinetic pathways, and rate-limiting steps related to harmful chemical reactions. By contrast, mesoscopic simulation studies on SOFC failure focus on particle behavior and interactions during SOFC preparation or operation processes, while macroscopic degradation simulations of SOFCs mainly involve embedding the degradation mechanisms obtained through micro/mesoscopic work into existing MPMs to establish a mapping relationship between the micro/mesoscopic degradation mechanisms and macroscopic performance. The impact of soft failure issues on reversible SOFC performance at the macroscale was thoroughly summarized by Yang et al.^5^ However, their literature review did not include work on SOFC transient apparent output performance evolution and lifespan prediction in the presence of degradation issues. Moreover, SOFC transient models considering degradation issues have not been fully summarized. A comprehensive literature summary and discussion of these issues are presented below.

The key to developing an SOFC performance degradation model lies in defining the degradation mechanism. While many soft failure issues demonstrably affect SOFC performance, most mechanisms are challenging to accurately describe using mathematical models, making their quantification difficult. Currently, the degradation mechanisms that can be described by mathematical equations mainly include anode carbon decomposition, Ni particle coarsening, Ni migration (in SOEC mode), Cr poisoning in the cathode, interconnector oxidation, and reduction in intrinsic conductivity of YSZ in the electrolyte. Table 14 summarizes the mathematical models for these degradation mechanisms. Notably, most of the degradation mechanisms in Table 14 are semiempirical and semitheoretical models. Specifically, these degradation model frameworks are typically proposed based on the failure mechanism, with the parameters determined experimentally. Given that long-term performance degradation experiments (>4380 h, approximately half a year) are time-consuming and expensive, such data are rarely reported. Although some long-term data have been documented in the existing literature, they are not specifically tied to a particular attenuation mechanism. For example, Bernadet et al.^272^ tested an SOFC for >14,000 h. The main purpose of this experiment was to confirm the inhibition effect of the barrier layer prepared by laser pulse deposition on cation diffusion and reaction at the electrolyte–cathode interface. Lim et al.^273^ tested the performance of a large area anode-supported SOFC for approximately 4400 h. The main purpose of this work was to explore the effects of fuel and air utilization on cell durability. Thus, most degradation equations can only be validated via attenuation data obtained from relatively short operation times. Validating the use of such equations in long-term degradation models is challenging, highlighting a limitation of these equations. However, this limitation underscores the importance of developing appropriate degradation equations. When obtaining SOFC attenuation performance through experiments is difficult, degradation equations can be extrapolated to reasonably predict attenuation performance. The numerical simulation studies on transient SOFC degradation performance are summarized below.

**Table 14:** Mathematical Models for Different Degradation Issues

issues	description	model	remark	ref
Anode carbon deposition	Carbon deposition rate		The carbon deposition rate is determined by the carbon deposition rate, which also leads to a decrease in reactivity activity, porosity, and permeability.	(126, 274)
	Reaction activity *a*			
	Porosity			
	Permeability			
Anode Ni coarsening^a^	Ni particle radius		The diameter of Ni particles increases, which leads to changes in TPB density, the specific surface area of Ni particles, and electrical conductivity.	(133, 275, 276)
	TPB density^b^	λ = 2π min (*r*_el_, *r*_io_) sin θ · *w* · *Nξ*_el_*Z*_el-io_*P*_el_*P*_io_		
	Ni specific surface area^b^	*A*_Ni_ = 2*πr*_Ni_^2^(2 – (1 – cos θ) ·*Z*_el-el_ – (1 – cos θ)· *Z*_el-io_) · *Nξ*_el_		
	Effective electronic conductivity	σ_*i*_^el, eff^ = σ_*i*_^el,0^((1−ε)ψ_*i*_*P*_el_)^γ^		
	Effective ionic conductivity	σ_*j*_^io, eff^ = σ_*j*_^io,0^((1−ε)ψ_*j*_*P*_io_)^γ^		
Cathode Cr poisoning	TPB density		The Cr oxidation rate causes a change in cathode TPB density.	(268, 277)
	Rate of Cr oxidation			
Interconnector oxidation	Wagner’s law for oxide layer growth		Growth of the oxide layer on the interconnector leads to an increase in electrical resistance.	(271, 278)
	Conductivity of the oxide layer			
	Area-specific-resistance for oxide layer			
Electrolyte YSZ phase transition	YSZ ionic conductivity	c	The crystal structure of 8YSZ changes from a cubic to tetragonal phase, resulting in a decrease in conductivity.	(279)
Anode phosphine (PH_3_) poisoning^d^	PH_3_ surface coverage θ		The formation rate of PH_3_ affects the surface coverage and molar fraction of PH_3_, thus changing the anode porosity, conductivity, and exchange current density.	(280, 281)
	PH_3_ molar fraction *y*			
	Reaction source term ω_θ_	*ω*_*θ*_ = *k*_f_*y*_PH_3__(1 – θ) – *k*_b_θ		
	Porosity	ε = ε_0_(1–0.95*θ*^*p*^)		
	Electrical conductivity	σ = σ_0_(1–0.95*θ*^*q*^)		
	Anode exchange current density			

a Various models for Ni particle growth have been proposed, which were summarized by Fu et al.^133^ The model proposed by Sehested et al.^282^ was used here, and the parameters in the model were determined by Fu et al.^133^ through experimental data.

b The TPB density and specific surface area of Ni particles were calculated based on the binary random filled sphere (BRFS) model, proposed by Chen et al.,^283,284^ which is an ideal model for relating the microscopic particle radius to macroscopic parameters. The specific details of the BRFS model can be found in refs.^85,133,276,283,284^

c Nakajo et al. obtained a more complex equation by fitting the experimental attenuation data of YSZ ion conductivity at different temperatures. For details, please refer to ref.^278^

d The PH_3_ poisoning phenomenon occurs only when the fuel gas contains phosphorus.

Ni coarsening is an inherent degradation factor because SOFC anodes can produce steam and Ni particles will grow in the humidified environment. Fu et al.^133^ incorporated a model for Ni particle coarsening into an MPM for direct internal reforming SOFCs and predicted the voltage attenuation curve. The authors investigated the effects of operating temperature, S/C ratio, current density, initial Ni particle diameter, and YSZ diameter, optimizing these parameters to decrease the voltage attenuation rate. Parametric analysis and optimization achieved an average voltage attenuation rate of 1%/1000 h, meeting the requirement for stable operation of commercial SOFCs.^264,285^ In our previous study,^276^ a transient MPM of an SOFC fueled by syngas was developed considering the Ni particle coarsening mechanism. The results indicated that reducing the attenuation rate by optimizing the operating and structural parameters could reduce the output power. After optimization, the average current density reached 5848 A·m^–2^ at an operating voltage of 0.6 V, with the attenuation rate not exceeding 1%/1000 h. Zhu et al.^275^ established a transient SOFC model that comprehensively considered Ni coarsening, interconnector oxidation, and electrolyte-phase transfer failure factors. The Ni particle growth model developed in this study differed from that listed in Table 14. The simulation results indicated that the cell attenuation rate in long-term operation was 2.2%/1000 h. However, through reasonable electrode microstructure design and interconnector coating, the authors were able to reduce the attenuation rate to 0.43%/1000 h, meeting the stability requirement of commercial SOFCs. Rizvandi et al.^286^ developed a 3D transient degradation performance model for an SOFC stack, considering Ni coarsening, Cr corrosion, and interconnector oxidation. This model predicted the operation performance of an SOFC stack over 38,000 h. The authors also explored the influence of different operating conditions on degradation performance, demonstrating that potentiostatic operation, moderate temperature, low load current, and counterflow could effectively reduce the stack’s attenuation rate by optimizing the operating conditions. Nakajo et al.^278^ established a degradation model for SOFCs considering interconnector oxidation, electrolyte conductivity reduction, Ni particle coarsening, cathode Cr poisoning, and insulating phase formation. The results indicated that the cathode substantially contributed to SOFC degradation. The local overpotential predominantly controlled Cr contamination, which also promoted the generation of insulating phases during operation. Owing to the lack of more detailed and reliable experimental data and degradation mechanisms, numerical models can only guarantee qualitative agreement with existing experimental data. In short, these studies provide a comprehensive summary of the degradation mechanisms and their impact on the electrochemical performance of SOFCs.

**Figure 42:**
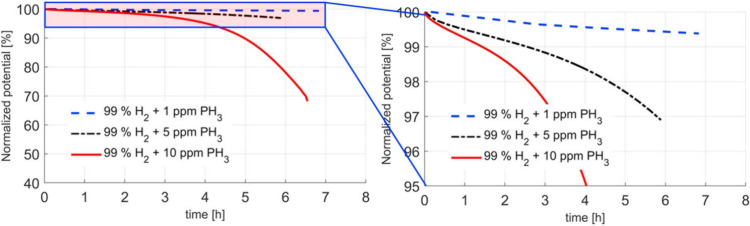
Voltage drops of an SOFC in atmosphere with different phosphine concentrations. Reproduced with permission from ref (280). Copyright 2020 Elsevier.

The presence of impurity gas components in the fuel gas, such as phosphides, sulfides, selenides, etc., can also lead to the degradation of cell performance. Sezer et al.^280^ developed a transient degradation model for a 3D single-channel SOFC to study the effect of phosphine in coal syngas on cell performance. The model parameters were calibrated experimentally using button cells. The simulation results indicated that phosphine coverage substantially affected the current and temperature distribution, leading to substantial degradation of cell performance, as shown in Figure 42. SOFC degradation became more severe with increasing phosphine concentration. Cayan^281^ developed a 1D anode transient model to investigate the effect of impurities (AsH_3_, PH_3_, H_2_S, and H_2_Se) on SOFC electrochemical performance. The parameters in the degradation model were calibrated for different impurities and the calibrated model was utilized to predict SOFC performance in an impurity-containing atmosphere, with the predicted results aligning well with reported experimental data.

Carbon deposition is a thorny problem for carbon-based SOFCs. The previously described steady-state models can only support a judgment about whether carbon is deposited. The main concern of such models is the thermodynamic aspects of carbon deposition.^113,287^ The chemical reactions of carbon deposition are generally believed to be CH_4_ decomposition, the Boudouard reaction, and CO hydrogenation, as shown in eqs 82–84.
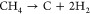
82
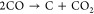
83
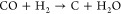
84

The deposition activity α determines the degree and trend of thermodynamic carbon deposition, as shown in eqs 85−87. If α > 1, thermodynamic carbon deposition will occur.^288^
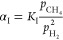
85
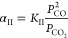
86
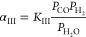
87where *K* is the reaction equilibrium of the above carbon deposition reactions.

The carbon deposition model described above does not account for the impact of deposited carbon on SOFC performance and can only predict whether carbon deposition occurs and the trends of carbon deposition reactions. However, given that deposited carbon accumulates over time, its influence on SOFC performance becomes increasingly important. To address these issues, transient carbon deposition models have been developed that couple the carbon deposition reaction kinetics with multiphysics field models of SOFCs. Transient carbon deposition models assume that the deposited carbon will cover the electrode particles, eventually blocking the electrode pores, which will increase gas flow resistance and reduce chemical/electrochemical reaction rates. Yan et al.^274^ developed a transient model for an SOFC fueled by a mixture of H_2_O, CH_4_, and H_2_ and considered variable porosity to explore the effect of carbon deposition on SOFC performance. The results indicated that the contribution of anode porosity to electrochemical performance was approximately 7%, highlighting the importance of considering porosity variation when dealing with carbon deposition. Using this model, the inhibitory effects of different operating parameters on carbon deposition were also investigated.^289^ Carbon tended to prominently accumulate at the entrance of the ASL, whereas the concentration of deposited carbon was comparatively lower at the outlet region. Consequently, the electrochemical performance of the ASL near the entrance was poor, while that of the ASL at the exit was superior. Additionally, the results indicated that increasing the operating voltage, inlet H_2_ fraction, operating pressure, and temperature could accelerate the carbon deposition process. Our group^126^ developed a 2D SOFC model considering the effects of carbon deposition and temperature on porosity, reaction activity, and permeability, but not the effects on TPB density and effective conductivity. The simulation results indicated that the carbon concentrations were approximately 30,000 and 90,000 mol·m^–3^ at 42 and 140 days, respectively. In addition, the current density was reduced by approximately 1.6% and 6% at 42 and 140 days, respectively, which did not meet the attenuation rate requirement of commercial SOFCs. Additionally, the comprehensive effects of the operating parameters on current density, current density attenuation rate, and temperature distribution were considered.

Given that long-term degradation tests of SOFCs are time-consuming, limited data are available to calibrate the parameters involved in these mechanisms. Most studies only validate degradation mechanism equations using experimental data. Although a few research groups have tested and reported long-term operation data of SOFCs, the cell degradation mechanisms and boundary conditions corresponding to these data may not be sufficiently clear for specific model validation. As a result, directly validating transient degradation models with existing long-term operation data is difficult. Additionally, the effects of these degradation equations on important SOFC performance parameters, such as the influence of Ni particle coarsening on TPB density and conductivity or the impact of carbon deposition on porosity and reactivity, have not been rigorously examined. Such validations may need to be conducted at a mesoscale, given that the acquisition of certain parameters encompasses the mesoscopic structure of electrodes, such as the TPB density and porosity. Therefore, the current validation of SOFC transient models is not rigorous or sufficient. These models can help to understand the mapping relationship between degradation mechanisms and apparent SOFC performance of SOFC, thus aiding in predicting their operating lifespan. To obtain more accurate transient degradation models in future work, the following points should be noted:

- More experimental data are needed to validate the reliability and accuracy of degradation mechanism equations.
- The interaction between degradation mechanisms and key SOFC parameters should be further verified through in situ testing or observation.
- The apparent SOFC performance (such as voltage or current density) obtained from a transient degradation model should be compared with experimental data to further confirm the model’s rationality and reliability.
- It is imperative to construct reasonable and reliable antidegradation strategies. A balance must be struck between mitigating various degradation factors. The trade-off between life extension and preferable performance should be considered. For example, if the S/C ratio increases, the risk of carbon deposition will be reduced, while SOFC performance will be reduced due to fuel dilution. Increasing the inlet gas temperature will improve SOFC performance, but it will also increase the carbon deposition rate, thus to increase the risk of carbon deposition. Therefore, it is necessary to establish the optimization model that considers both degradation performance and electrochemical performance, so as to optimize and screen the operating conditions.

### 3.3 Combining SOFC Models with AI

Generally speaking, SOFC MPMs, especially 3D models, are characterized by strong coupling, complex solutions, and long solution times, making real-time predictions and online optimization challenging. To ensure safe and efficient long-term operation of SOFCs, quickly optimizing and controlling the operating parameters to avoid thermal failure or other issues is necessary.^290^ While they provide precise results, MPMs are not conducive to the development of model-based control systems with fast response times.^291^ While they provide precise results, MPMs are not conducive to the development of model-based control systems with fast response times

**Figure 43:**
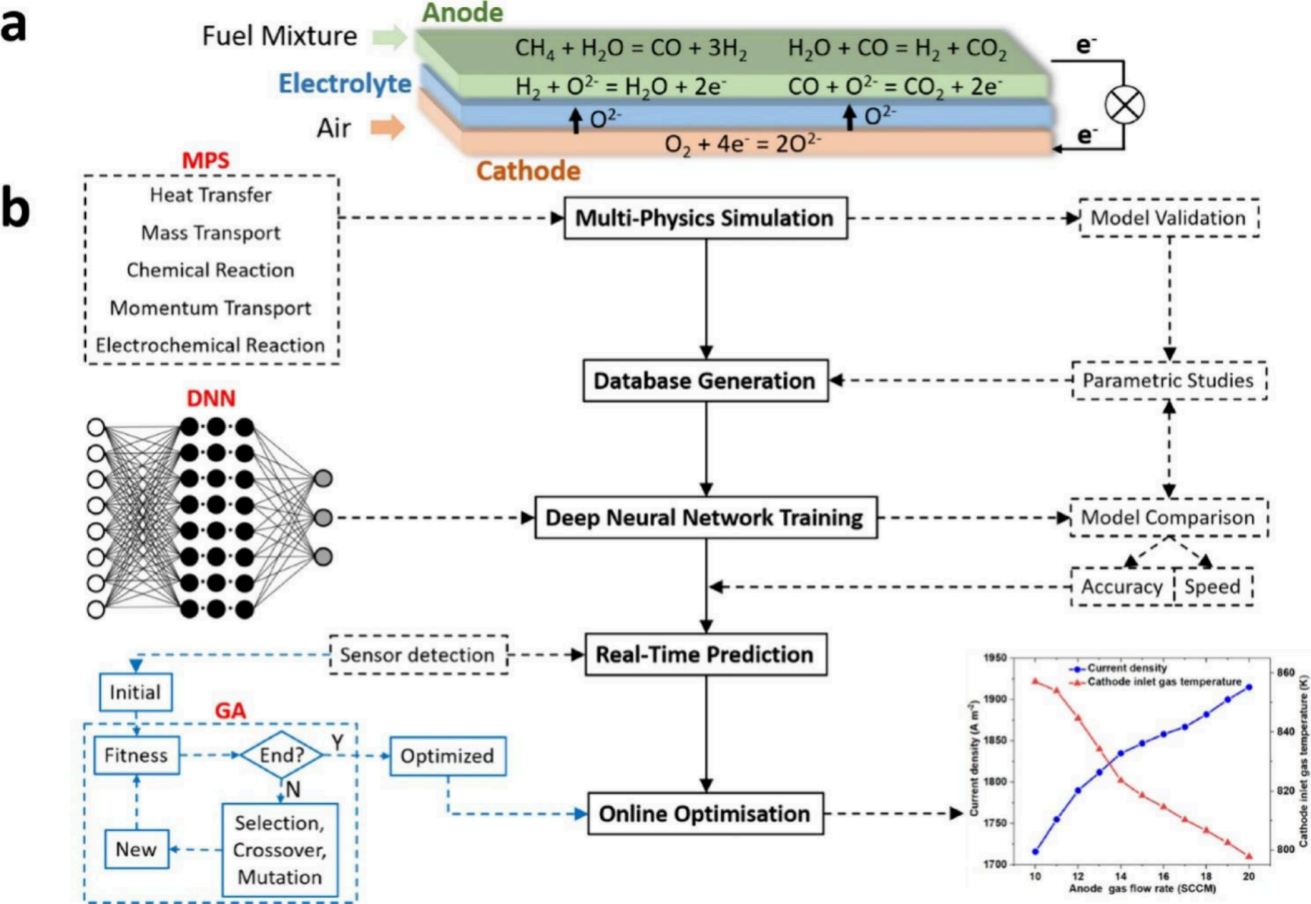
Schematic of the model framework for the combination of a multiphysics field model and ANN. Reproduced with permission from ref (291). Copyright 2020 Elsevier.

Xu et al.^291^ proposed a model framework that combined MPM with AI to address the complexity of optimizing and controlling SOFC systems based on physical models. The main framework of the model is illustrated in Figure 43. Initially, a validated SOFC MPM was established, generating a 17,384 × 11 data matrix comprising 17,384 × 8 input and 17,384 × 3 output data matrices. The input data set primarily consisted of various operating parameters, such as current density, anode and cathode gas flow rates, and temperatures at five points. The output data set included parameters related to SOFC performance, such as heat production and the temperature gradient. Subsequently, these data sets were utilized to train the artificial neural network (ANN). The results demonstrated that the well-trained ANN model could achieve precise prediction of SOFC performance, with the error between the MPM and ANN within 1%. Moreover, combining the GA with the ANN enabled online optimization of SOFC performance. The specific optimization objective was to limit the temperature gradient and operating conditions within reasonable boundaries while maximizing the power density.

Wang et al.^292^ developed a 2D MPM of SOFCs and generated data sets for training the ANN. Their study focused on the impacts of carbon deposition and temperature gradient on SOFC performance. Consequently, the input data set included parameters such as the steam-to-CH_4_ ratio, operating voltage, fuel velocity, and operating temperature. The output data set comprised the current density, average carbon deposition rate, and maximum temperature gradient. Subsequently, a multiobjective GA was utilized to optimize the ANN surrogate model to achieve the dual goals of minimizing the carbon deposition rate and maximizing the power density. A similar modeling framework was proposed in our previous work to construct an SOFC degradation performance prediction and optimization model considering Ni particle coarsening.^276^ A traditional MPM typically requires several to even 10 min to solve, rendering it challenging to utilize a traditional MPM for swift optimization of SOFC performance. Conversely, an ANN can offer a prediction outcome within only one second, making it ideal for rapid optimization of SOFC performance. With this framework, the degradation performance of an SOFC under Ni particle coarsening could be reasonably predicted and optimized. The results indicated that the average relative errors of the predicted attenuation rate and current density were 0.767% and 0.248%, respectively, indicating the reliability of the ANN. Under the conditions that the limited attenuation rate was <1%/kh and the operating voltage was 0.6 V, the optimized average current density and attenuation rate were 5701 A·m^–2^ and 0.945%/kh, respectively.

Wang et al.^293^ proposed a tubular SOFC structure coupled to a glycerol in-tube reformer and developed the 2D axisymmetric MPM. First, four inlet parameters, including tube reformer thickness, anode tube size, anode flow rate and anode glycerin content, and four outlet parameters, including current density, average temperature, anode syngas flow rate and maximum temperature difference were determined by parameter analysis. The, the authors uses ANN to construct the mapping relationship between input and output variables, and obtained a surrogate model to replace MPM. Finally, the authors used GA to optimize the surrogate model to improve the performance of the tubular SOFC.

Ba et al.^294^ introduced a novel modeling concept termed “alternative mapping” to address the time-consuming nature of solving MPMs for SOFC stacks. The core idea of this concept was to utilize an ANN to replace the complex nonlinear governing equations. The new model comprised the unit cell model, ANN, and stack model. The unit cell model was a physics-coupled model with reduced geometric dimensions, which was primarily employed to generate a large data set for training the ANN. The input data set for the ANN included position coordinates, anode H_2_ mass flow rate, cathode O_2_ mass flow rate, temperature, and voltage, while the output data set included reaction and heat source terms. Subsequently, the mapping function derived by the ANN between the input and output data sets was incorporated into the stack model to reduce its nonlinearity and computation time. This innovative approach demonstrated a reduction in solving time of 1/7 of that of the traditional fully coupled stack MPM.

**Figure 44:**
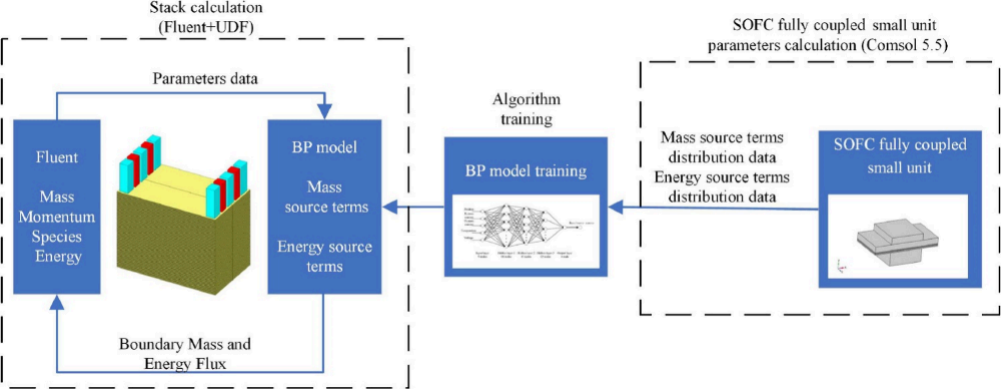
Model framework of a large stack SOFC simulation. Reproduced with permission from ref (295). Copyright 2022 Elsevier.

Xiong et al.^295^ introduced a large stack simulation framework based on alternative mapping, which combined the advantages of two commercial software packages, COMSOL and Fluent. The framework, as depicted in Figure 44, began by modeling a small SOFC unit with full physics field coupling in COMSOL software, generating a database of mass and energy source term distributions. Next, the mapping relationship between the input boundary conditions of small cells and the source terms was established using a backpropagation neural network. Finally, the mapping relationship was coupled to Fluent through user-defined functions to achieve decoupling of the large stack multiphysics field model. This framework substantially reduced the modeling time while maintaining a high level of accuracy. The study also compared the solution time and accuracy between the coupled MPM and alternative mapping model. The alternative mapping model reduced the solution time and memory usage for a one-layer case compared to the fully coupled model. The times required to solve the single-channel, 10-channel, and one-layer models using the alternative mapping approach were approximately 11027, 10931, and 11315 s, respectively, while the times required by COMSOL were 863, 14479, and 30972 s, respectively. When solving complex large cell models, the alternative mapping model demonstrated better performance than the fully coupled model. The maximum errors were 4.39% for the H_2_ molar fraction, 1.18% for the O_2_ molar fraction, and 1.09% for temperature.

Chi et al.^296^ employed data-driven surrogate cell submodels to simplify the SOFC stack MPM by replacing part of the governing equations. In their study, the adaptive polynomial approximation (APP) method was utilized to fit partial differential equations rather than an ANN. The APP method offers advantages such as low sampling cost and direct integration into commercial computational fluid dynamics (CFD) software. The simulation results demonstrated that the simplified stack model accurately predicted the temperature and voltage. Moreover, for a 7-cell stack, the simulation memory and time were reduced by approximately 60%, while the computation time was reduced by >70% for a 21-cell stack.

In recent years, two model frameworks combining MPMs and ANNs for SOFCs have emerged to address the challenge of long solution times for MPMs: (1) a model framework that directly replaces the MPM with a surrogate model created using deep learning, as shown in Figure 43; and (2) a model framework that substitutes some governing equations to decouple the MPM and reduce the solution time, as shown in Figure 44. Both frameworks have shown promising results. In particular, the second framework notably reduces the solution time of the SOFC stack MPM. While this reduction in solving time may slightly reduce the model’s accuracy, the error remains acceptable for practical applications. Therefore, the coupling of MPMs and AI provides an effective approach to accelerate MPM solutions or achieve fast responses. Overall, model frameworks combining MPMs and ANNs are worthy of further R&D and expected to accelerate stack model solving and the development of fast response control systems.

**Figure 45:**
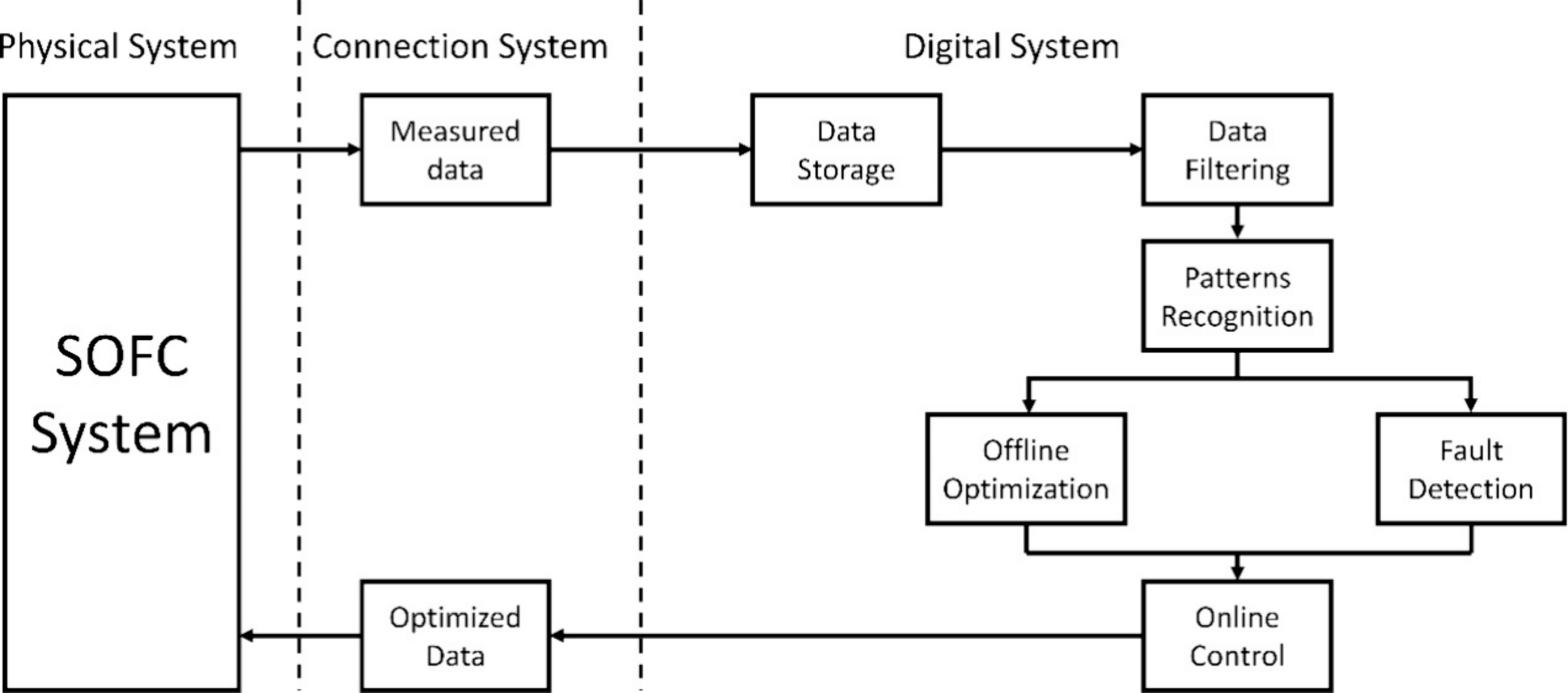
Schematic of SOFC digital twin architecture. Reproduced with permission from ref (301). Copyright 2024 Elsevier.

At present, the combination of MPMs and ANNs has achieved remarkable advancements in fast solution and real-time prediction. The combination of SOFC models and AI will transition to a more advanced stage, namely, the combination of SOFC models and digital twins (DTs) and the combination of SOFC models and generative adversarial networks (GANs). A DT virtually simulates the behavior of a physical system by acquiring real-time data that are updated over its lifetime. A DT replicates a physical system to observe and evaluate its operation, thereby enabling failure prediction and identification of opportunities for change, facilitating the development of real-time measures and optimization of equipment or systems.^297^ The fundamental architecture of DT models is presented in Figure 45.^298^ First, measurement data are generated by physical equipment or systems, which are imported into a digital system. Through data storage, data filtering, and pattern recognition, offline optimization and fault detection information is generated, which is converted into online control signals and fed back to the physical system. At present, few studies report the combination of DTs and SOFCs. Nevertheless, future research is expected to combine DTs and SOFCs to achieve online control and optimization. A GAN is an unsupervised deep learning model that is mainly employed to generate data by computer. To date, GANs are mainly applied in sample data, image, and text generation, among other directions. In view of the advantages of GANs in image generation, some scholars have applied GANs to the generation of SOFC electrode slice images.^299,300^ GANs can manipulate the geometric and statistical properties of electrodes, enabling the generation of electrode slices tailored to specific requirements and facilitating the targeted study of mesoscopic electrode structure. In addition, the use of GANs to generate electrode images can reduce research costs and time given that obtaining the electrode structure by traditional experimental means requires a large amount of time and expense. DTs and GANs are expected to be widely applied in the field of SOFC modeling in the future, which will greatly promote the application of AI to solve problems that were difficult to solve in the past.

### 3.4 Challenges and Prospects of Cell/Stack Models

The development of SOFC multiphysics field models has rapidly progressed and model frameworks have matured, with MPMs becoming a powerful tool for gaining insight into the inner workings and optimizing the design of SOFCs. Additionally, commercial software such as COMSOL and Fluent has successfully developed SOFC simulation modules, greatly reducing the complexity of solving MPMs and advancing SOFC simulation technology. Nevertheless, despite progress in multiphysics simulation techniques, two issues warrant further discussion and research: reliability and practicality. Currently, reliability is confirmed through model validation, yet existing validations focus on *I–V* curves with few reports addressing parameters such as temperature, flow, and species concentration fields and attenuation curves. Although validating these parameters is essential, experimental measurement is often challenging. Therefore, ensuring reasonable validation of crucial parameters (apart from *I–V* curves) and confirming model reliability are key concerns for future modeling work. In addition, most SOFC simulation work is presently conducted for theoretical research purposes only, with the conclusions rarely applied to practical SOFC fabrication or operation, leading to a loss of practicality in simulation research. This situation is understandable given that most of the reported work is performed at research institutions with few contributions from SOFC fabrication or operation enterprises. Future work should focus on conducting reasonable design and operation optimizations based on practical issues using simulation technology. This approach can guide SOFC manufacturing and operation, leveraging the full value of multiphysics simulations.

## 4 Development of Mesoscopic Models

### 4.1 Overview of Mesoscopic Models

The above-mentioned studies provide a review of SOFC modeling, primarily focusing on system and cell/stack scales. However, owing to the complex porous electrode structure, answers to some questions must be sought via smaller scale models, such as meso- or microscopic models. For instance, microstructural parameters such as TPB density and the specific surface area of Ni particles are typically set as average values in cell/stack simulations. Their exact values can only be extracted via mesoscopic electrode simulations. The impact of these microscopic parameters on SOFC performance can be fully understood at the microscale or mesoscale, especially the TPB density, which directly affects electrochemical performance. Moreover, accurately simulating several reaction processes at the mesoscopic electrode scale is imperative to derive precise reaction pathways and elucidate the mechanisms that influence cell performance. In short, mesoscopic electrode simulations provide the necessary parameters, refined reaction-transfer mechanisms, and reliable performance degradation mechanism for cell/stack simulations. Macroscopic cell/stack models can couple parameters, reaction kinetics, and degradation mechanism equations obtained from mesoscopic models, thereby enabling the development of more reliable large-scale models to guide the development and optimization of large-scale SOFCs. In fact, cell/stack models are a practical application of the parameters and mechanisms obtained from mesoscopic models, making them more suitable for application to actual large SOFCs.

**Figure 46:**
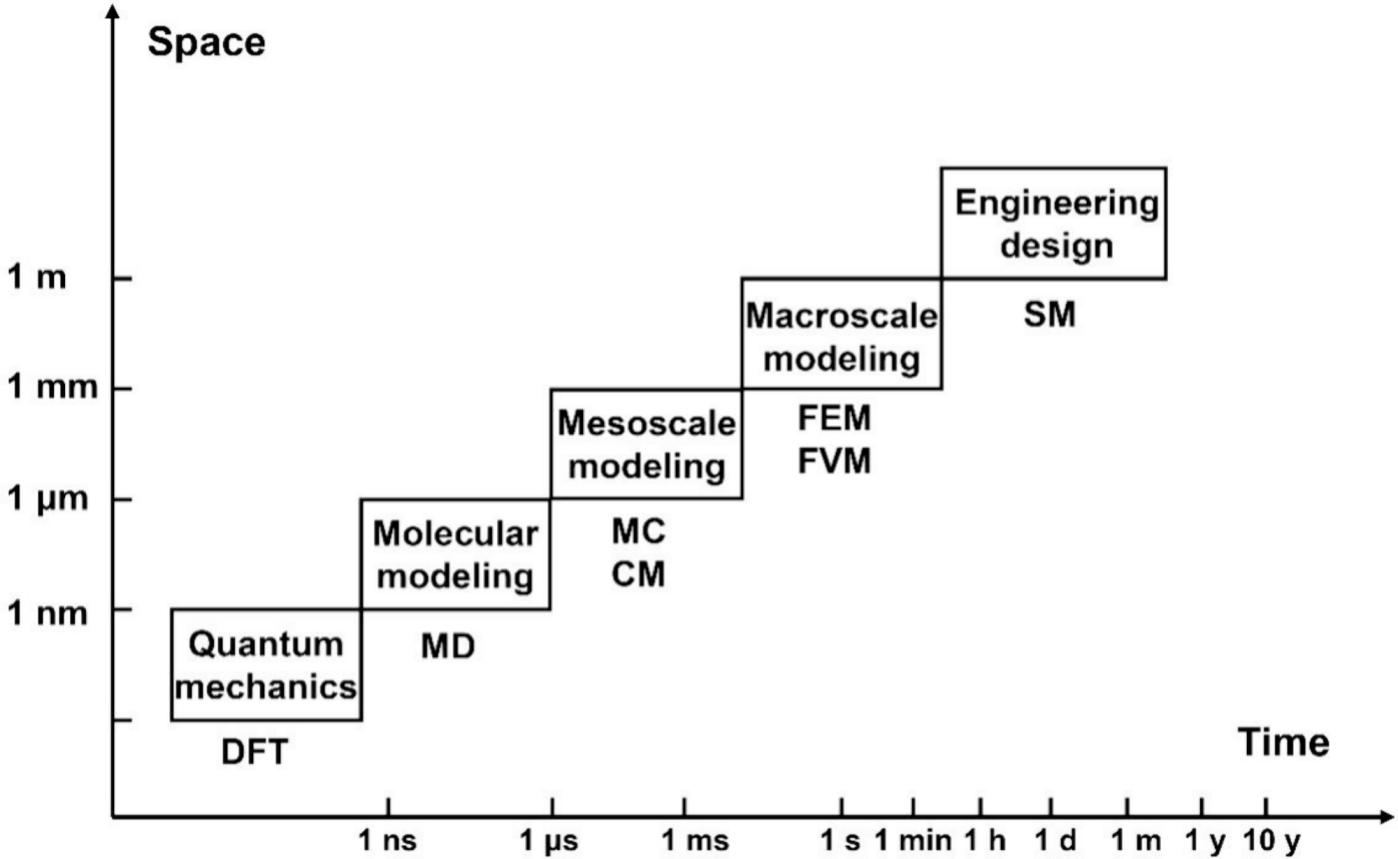
Length and time scales for different SOFC models. Reproduced with permission from refs (3, 103, and 302). Copyright 2007, 2010, 2021 Elsevier.

The research scale can be categorized based on time and length, as illustrated in Figure 46.^3,103,302^ Microscopic models, within the micrometer scale, primarily assist in developing functional materials. These include quantum mechanism research and molecular simulations, employing theories like DFT^303,304^ and molecular dynamics (MD).^305,306^ Mesoscopic models focus on microstructures or interfaces, utilizing methods like Monte Carlo (MC),^307,308^ the Lattice Boltzmann Method),^309,310^ and the continuum method (CM).^311^ These models have a length scale ranging from micrometers to millimeters. Macroscopic models aim to simulate cell/stacks, focusing on homogeneous reaction-transfer mechanisms, essentially referring to the MPMs discussed in Section 3. Their length scale ranges from millimeters to meters, utilizing methods like FEM and FVM. Engineering-scale models primarily target large system operation or optimization design, employing system modeling, as discussed in Section 2, with length characteristics exceeding meters. The modeling scale of SOFCs not only considers length but also time. For example, the quantum mechanical model operates within 1 ns, while MD operates within 1 μs. The continuous model (including meso/macro-scales) operates in the range of microseconds to days. Engineering design scales range from several days to several years.^3^

Microscopic models, with the smallest length scale, focus on molecular- or atomic-scale simulations of electrode material performance. By contrast, mesoscopic models, with a larger length scale, concentrate on electrode structure or interface simulation, which are key factors affecting the electricity production performance of SOFCs. Additionally, mesoscopic models can demonstrate the reaction-transfer phenomenon within the electrode more precisely. This section primarily focuses on modeling porous electrodes or small cells. Molecular or atomic-scale simulations of electrode materials are primarily employed for developing excellent performance materials and optimizing electrolyte conductivity, catalyst surface reaction mechanisms, and so on. The fundamental theory of these research topics goes beyond the scope of thermodynamics and heat and mass transfer to the field of materials science. Therefore, this section mainly discusses mesoscopic modeling related to electrode structures and interfaces.

### 4.2 Research Progress of Mesoscopic Models

#### 4.2.1 MPMs Based on Mesoscopic Elementary Reactions

**Figure 47:**
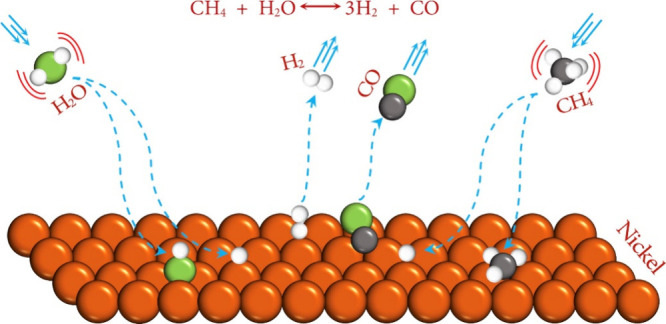
Schematic of the multistep elemental reactions of CH_4_ reforming on the surface of Ni particles. Reproduced with permission from ref (312). Copyright 2017 Elsevier.

The chemical reactions involved in previous MPMs are described by global reaction kinetics, as shown in Table 5 in Section 3.1. The global reaction kinetics describe the chemical reaction process by directly giving the reactants and products without considering the intermediate gas-phase adsorption and desorption. In fact, the reactions occurring in porous electrodes involve three kinds of elemental reactions: adsorption, desorption, and surface reactions, as shown in Figure 47. Global reaction kinetic equations have been widely used in previous MPMs to obtain satisfactory results.^32,33,36,128,140−142,145^ However, the global reaction model has difficulties dealing with the following problems:

(1) Obtaining insight into the gas–solid chemical reaction process is difficult via global reaction kinetics as the rate-controlling steps of the chemical reaction process cannot be determined.^313^ For example, the rate-controlling steps of the carbon deposition reaction must be clarified in the study of carbon deposition in porous anodes to reduce the carbon deposition rate.
(2) The global reaction model cannot address the case in which the chemical reaction kinetic parameters are unknown under specific operating conditions.^312^
(3) Most global electrochemical reaction kinetics are determined based on H_2_ and are not accurately applicable to syngas fuels with H_2_ and CO. The electrochemical reaction rate of H_2_ is reportedly 2.3 to 3.1 times larger than that of CO.^32^ However, this conclusion lacks a strict theoretical basis, and the electrochemical reaction kinetics of syngas fuel are not accurately calibrated in experiments.^313^

Considering the above issues, MPM-based mesoscopic elementary reactions have gradually developed. The main idea of this model is to replace the chemical and electrochemical global kinetics with elementary reaction kinetics. Notably, although the model involves mesoscopic elementary reaction mechanisms, solving the model continues to rely on FEM/FVM based on the continuum method. Studies investigating multiphysics simulations coupled with elementary reaction kinetics are reviewed herein.

One of the functions of elementary reactions is to predict chemical reaction pathways, making it is possible to precisely simulate the complex reaction processes that have multiple possible reaction directions, such as the CH_4_ reforming reaction. Goldin et al.^314^ developed a button cell MPM based on elemental reaction kinetics using in-house code. The model incorporated the 42 -step heterogeneous elementary reactions of MSR, the details of which are available in ref.^315^ This study aimed to assess the reliability of the assumptions that the cell is isothermal and gas composition within gas compartments is uniform and known. These assumptions are usually employed to explain button cell experimental results and from the basis of 1D SOFC models. The results indicated that the button cell data conformed to these assumptions when the inlet flow rate was sufficient. However, the button cell data significantly violated the assumption of uniform gas composition when the fuel flow was insufficient. Importantly, the elementary reaction kinetics of CH_4_ reforming can comprehensively describe reactions such as steam reforming, autothermal reforming, dry reforming, and catalytic oxidation. By contrast, conventional global reaction kinetics can only describe specific reactions through empirical expressions, as shown in the MSR and RM kinetics listed in Table 5. The elementary reaction model does not directly include specific global reactions; instead, specific global reaction pathways are represented by combinations of elementary reactions. Therefore, the elementary reaction model can predict multiple reaction paths under a wide range of operating conditions.

**Figure 48:**
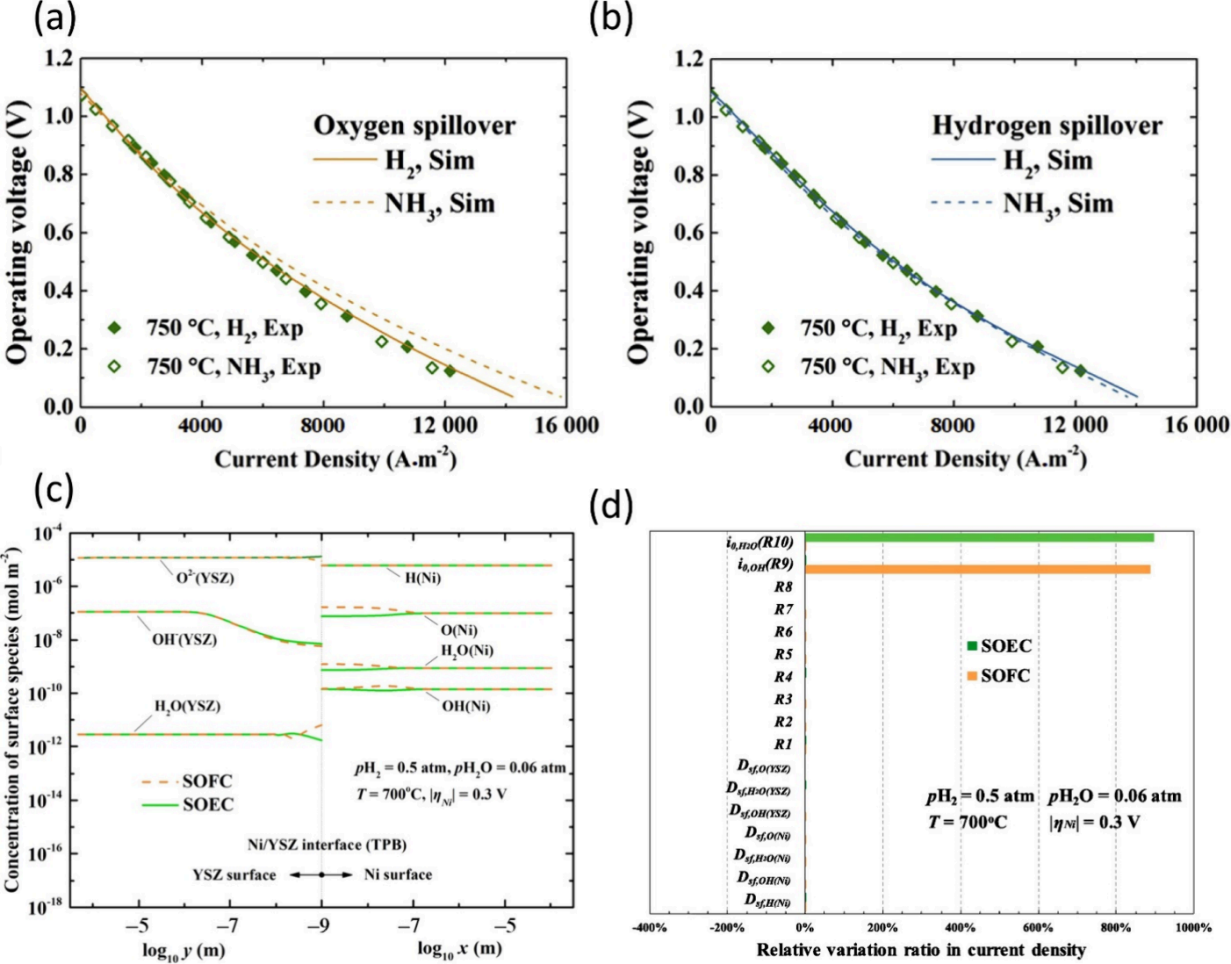
Validation of model of SOFC fueled by H_2_/NH_3_ with (a) O_2_ spillover and (b) H_2_ spillover. (c) variation of surface species concentrations in SOFC and SOEC modes with H_2_ spillover mechanism. (d) sensitivity of each charge transfer reaction, surface elementary reaction, or surface diffusion coefficient in SOEC and SOFC modes. R9 and R10 refer to eqs 88 and 89, respectively. Reproduced with permission from refs (316, 317). Copyright 2019 Elsevier.

Another application of elementary reaction theory is to simulate the electrochemical reaction process in detail and determine more reliable charge transfer paths for different operating conditions. For example, Luo et al.^316^ developed a 1D button cell MPM considering NH_3_ direct decomposition elementary reaction kinetics. The model consisted of three modules that include charge/gas species/surface species transfers but did not consider energy conservation and momentum transfer. The O_2_ and H_2_ spillover mechanisms were employed to describe the electrochemical reaction process. Comparing the simulation and experimental *I–V* curves, the H_2_ spillover mechanism was found to be a better fit with the experimental data, as illustrated in Figure 48(a, b). Furthermore, the rate-determining steps and main surface species of the SOFC fueled by H_2_ and NH_3_ were determined by calibrating the model with the experimental data. Luo et al.^317^ developed a 1D button cell model with Ni-patterned electrodes to assess the difference in the electrochemical reaction mechanism between SOFC and SOEC in an H_2_–H_2_O atmosphere. The patterned electrodes had precisely controlled TPB length and Ni surface areas, which were used to study the electrochemical reaction mechanism.^317^ The two-step H_2_ spillover mechanism, as expressed in eqs 88 and 89, was employed to describe the charge transfer process. As shown in Figure 48(c, d), the rate-determining step of charge transfer, regardless of SOFC/SOEC mode, was the elementary reaction of OH^–^ (YSZ) generation because the surface coverage of OH^–^ (YSZ) < 1%.
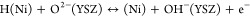
88
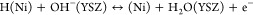
89

Li et al.^318^ established a similar model to study the effects of operating temperature, air electrode microstructure, and thickness on SOFC/SOEC performance. The results indicated that the influence of these parameters on SOFC/SOEC performance significantly differed. Thus, the optimization strategy for these parameters in SOFC/SOEC modes should also differ, particularly for thick air electrodes. Li et al.^319^ developed a 2D axial-symmetrical button cell MPM based on H_2_–H_2_O elementary reaction kinetics and the O_2_ spillover mechanism. The influences of the related operating parameters and fuel channel tube diameter on cell performance were investigated. The results indicated that the electrochemical reaction process mainly occurred at the anode–electrolyte interface within 20 μm. The surface of the Ni catalyst was mainly covered by H(s). The gas- and surface-phase species concentration distributions were presented. Xie et al.^320^ developed a 2D axial-symmetrical button cell model coupling the 5-step H_2_–H_2_O elementary reaction and O_2_ spillover electrochemical reaction kinetics. The simulation results indicated that the O(s), OH(s), and H_2_O(s) concentrations at the anode–electrolyte interface significantly increased with increasing temperature; however, the H(s) concentration was basically unaffected. The O(s), OH(s), and H_2_O(s) concentrations were very sensitive to fuel composition and voltage. This conclusion was consistent with the results obtained by Li et al.^319^ In the study by Xie et al.,^320^ the O(s) concentration was highest in the anode, followed by the H(s) concentration. However, in the study by Li et al.,^319^ the H(s) concentration was the highest in the anode. The inconsistent conclusions reached by these studies was attributed to the use of different operating conditions: Xie et al.^320^ and Li et al.^319^ obtained the species concentration distributions at 0.5 and 0.6 V, respectively. Additionally, according to the results of Luo et al.,^316^ although the H_2_ spillover mechanism described the experimental data of the H_2_-H_2_O electrochemical reaction more closely than the O_2_ spillover mechanism, the latter also provided a relatively reliable description of the H_2_-H_2_O electrochemical reaction. Using the H_2_ spillover mechanism to describe the H_2_-H_2_O electrochemical reaction as much as possible is recommended in subsequent research.

Yang et al.^313^ developed a reversible 2D SOFC steady-state MPM based on 20-step WGS elemental reactions kinetics. The authors found that the charge transfer process was controlled by the O_2_ spillover mechanism rather than the H spillover mechanism when the SOFC was fueled by syngas. Therefore, the O_2_ spillover mechanism was generally utilized to describe the electrochemical reaction kinetics. The microscopic electrode parameters, such as the TPB density and Ni particle surface area, were estimated using the BRFS model. The simulation results indicated that desorption and adsorption reactions mainly occurred near the electrolyte and flow channel. The surface species distribution depended on the gas diffusion process within the porous electrode. Given that gas reacts on the catalyst surface and diffuses through tortuous pathways, microstructure parameters such as porosity, tortuosity, and particle diameter greatly impact elementary reactions. In general, global reaction models tend to overestimate cell performance by not considering the limitations of local elementary reactions. By contrast, elemental reaction models can more accurately predict cell performance than global reaction models, confirming their accuracy and reliability. Based on the steady-state model, a transient MPM based on elemental reactions kinetics was further established.^321^ The dynamic simulation results indicate that SOFC performance needed >30 min to stabilize owing to gas diffusion limitations. Higher porosity and lower tortuosity facilitated gas diffusion, contributing to faster acquisition of stable performance.

Menon et al.^322^ developed a 2D planar SOFC-H model using syngas as the fuel, considering the 42-step CH_4_ reforming elementary reactions and H spillover electrochemical reaction mechanism. The specific H_2_ overflow mechanism is described by eq 90. The variable el refers to the electrode/electrolyte. The absorbed H atom (H(Ni)) vacates the Ni site (Ni), migrates to the electrolyte, releases electrons (e^–^), and generates protons (H^+^(el)). Since proton-conducting electrolyte was used, the electrochemical reaction of CO did not need to be considered. The influence mechanisms of the different operating conditions on temperature distribution, species transport, and electrochemical performance were explored. The H_2_ spillover mechanism used in this study differed from that employed in previous studies. Because the modeling object of this study was SOFC-H, the electrolyte only allowed for H^+^ transportation.
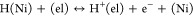
90

The above simulation studies on electrochemical elementary reactions demonstrate that the H_2_ spillover mechanism is more appropriate for SOFC-O fueled by H_2_ or NH_3_, the O_2_ spillover mechanism is more suitable for SOFC-O fueled by syngas, and the H_2_ spillover mechanism that considers H^+^ transport is more suitable for SOFC-H fueled by syngas.

The final application of the elementary reaction theory in SOFC modeling is to assess the risk of carbon deposition reaction occurrence and construct strategies for inhibiting this reaction. Yurkiv et al.^323^ conducted a detailed simulation of the anode carbon deposition mechanism in an SOFC using the elementary reactions theory, which differs from the transient MPMs presented in Section 3. The elementary reactions theory primarily investigates carbon deposition from basic carbon formation elementary reactions. By contrast, MPMs are based on global reactions and the apparent carbon deposition kinetics are determined from an experimental perspective. Thus, the elementary reactions theory better reflects the reaction mechanism and is more precise than global reaction–based MPMs. Schluckner et al.^324^ developed 3D single-channel models for an SOFC fueled by synthetic diesel reforming based on global and elemental reactions kinetics, respectively. The simulation results revealed that the global reaction kinetics mechanism accurately described the gas-phase reaction characteristics but could only estimate the thermodynamic trend of carbon deposition using eqs 85–87. By contrast, the heterogeneous elemental reactions kinetics could delve into detailed information on carbon deposition, such as the distribution of adsorbed carbon and the formation mechanism. The study demonstrated that the inlet region of the porous anode was particularly prone to carbon deposition because this region had the highest CH_4_/CO concentration. Higher operating temperatures and current densities helped to suppress carbon deposition. Li et al.^325^ developed a 1D MPM coupled with elemental reaction kinetics for a syngas-fueled button cell. The model was validated and calibrated using a self-built testing bench, and the effects of temperature and operating voltage on polarization loss, electronic current density, gas species concentration, and surface species coverage were investigated. The results indicated that higher temperatures and lower operating voltages helped to reduce the surface coverage of carbon and inhibited carbon deposition. Zhang et al.^326^ developed a 1D transient MPM of an SOFC fueled by syngas based on elemental reaction kinetics to investigate the effects of carbon deposition. The simulation results indicated that increasing the operating temperature, decreasing the operating voltage, and decreasing the molar fractions of CO and CH_4_ could effectively reduce surface coverage of carbon. Shi et al.^327^ developed a similar model to study the electrochemical performance and surface species coverage distribution of an SOFC fueled by a mixture of CO and CO_2_. The results suggested that increasing the temperature and decreasing the operating voltage could help to reduce the risk of carbon deposition on Ni surfaces. Meanwhile, increasing the CO content in the fuel would lead to a more serious degree of carbon deposition. This finding was attributed to increased temperature inhibiting CO decomposition, while a reduced operating voltage increased the current density, generated more O(s), and promoted C(s) oxidation. The above-mentioned studies applied elementary reactions theory to conduct precise simulations of the reaction paths of syngas/CO–fueled SOFCs and reached the same conclusions about optimizing the SOFC operating conditions to inhibit carbon deposition.

Yu et al.^328^ developed a 1D direct carbon SOFC model validated by experimental data obtained from fixed-bed carbon gasification experiments and a direct carbon SOFC testing bench. The model considered the elementary reactions for carbon bed gasification and electrochemical elementary reactions for CO oxidation. The experimental and simulation results indicated that using CO_2_ as the carrier gas could promote the forward Boudouard reaction and produce CO, thus improving cell electrochemical performance. The maximum power density of the cell could reach 213 W·m^–2^ at an operating temperature of 800 °C. Increasing the temperature was conducive to accelerating the rate of the elementary reaction that generated CO, thereby providing more CO for electrochemical reactions. Therefore, raising the temperature was beneficial to improving cell performance. The shortcoming of this study is that it did not consider the effect of carbon deposition caused by CO on cell durability.

In summary, the above-mentioned studies demonstrate the MPM coupling mesoscopic elementary reactions mechanism has the following three characteristics:

(1) The geometric structures of the models are relatively simple, mostly 1D or 2D, because the governing equations of surface species transport are added to previous MPMs, as expressed by eq 91.^313^
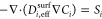
91where *D*_*i*,eff_^surf^ is the effective diffusion coefficient of surface species *i*; *C*_*i*_ is the concentration of surface species *i*; and *S*_*i*_ is the reaction source term.
  Each additional surface species is described by an additionally surface substance transport equation, which increases the difficulty of solving the model. For example, the 42-step CH_4_ reforming reaction mechanism contains 6 gas-phase species and 13 surface species, involving numerous species transport and reaction kinetics equations that result in a complicated model solution. Therefore, the modeling object in most studies was a button cell or single-channel SOFC where the cell geometry is reasonably simplified to 1D/2D to reduce the computational complexity.
(2) The focus of most elementary reaction models is the reaction mechanism, such as the CH_4_ reforming reaction path,^314,322^ charge transfer path,^316,317^ and carbon deposition reaction mechanism.^323,324,326^
(3) Although macroscopic global reaction kinetics are able to predict cell performance relatively accurately, addressing problems involving complex chemical/electrochemical reaction mechanisms remains challenging.^313,324^ Thus, developing MPM coupling elemental reaction kinetics is necessary to gain insight into the relationship between the reaction mechanism and cell performance at the mesoscale level.

The above characteristics of elementary reaction models provide guidance for selecting the appropriate model. If the research problem requires interpretation by the reaction mechanism, building a model based on elemental reactions may be beneficial. Otherwise, establishing a complex model based on elementary reactions may be unnecessary given that global reaction kinetics can predict cell performance with acceptable accuracy.

#### 4.2.2 MPMs Based on Microstructural Reconstruction

All of the aforementioned MPMs assumed that the porous anode was homogeneous. In other words, an average parameter, such as porosity or TPB density, was utilized to describe its characteristics. This approach was applied in MPMs based on mesoscopic elementary reactions. Similarly, surface species transport on Ni particles was described using a homogeneous theory akin to gas species transport. However, porous electrodes actually possess a complex microscopic pore structure and particle distribution, rendering average parameters inadequate for precise reflection of their characteristics. Moreover, acquiring average parameters relies on the microscopic morphology of porous electrodes. Additionally, the intricate gas transport path in the anode microstructure substantially impacts electrode performance. Hence, the porous electrode microstructure needs to be reconstructed and fine simulations of transport and electrochemical performance need to be conducted.

At present, microstructural reconstruction of porous electrodes is performed using experimental and numerical methods. Physical experiments involve using SEM and other high-resolution instruments to obtain planar images of porous electrodes. Subsequently, image processing technology is employed to generate a 3D porous medium model. Common tomography techniques include X-ray computed tomography (X-ray CT), focused ion beam-SEM (FIB-EM), electron tomography, and 3D atom probes. The resolution of these techniques sequentially increases, while the volume and voxel scale of the 3D structure obtained via these techniques gradually decrease. The choice of reconstruction technology depends on the sample’s natural properties and required size or precision of the 3D structure.

Zhang et al.^329^ provided a detailed description of the characteristics and resolutions of different tomography techniques. X-ray CT and FIB-SEM are most often employed in electrochemical applications and porous electrode reconstruction studies. Given that the particle diameter of porous electrodes is generally on the micron scale, a voxel scale of 10 nm to 50 μm is considered. However, tomography techniques are time-consuming and expensive. Only a small piece is selected as the sample, and the specific sample volume is approximately 100 μm^3^ to 10 mm^3^.^329^ Numerical methods offer an alternative porous electrode reconstruction approach, which can be divided into stochastic and process-based method s.^330^ The stochastic method, which utilizes statistical features such as porosity and pore distribution as reconstruction constraint functions to randomly generate 3D structures, includes the simulated annealing method (SAA), truncated Gaussian random field method (TGRFM), and Markov chain Monte Carlo method (MCMC). The process-based method reconstructs the 3D structure by simulating the formation process of porous media. Although numerical methods save time and cost, the reconstructed structure may not fully replicate the real structure owing to certain technical difficulties.

The commonly used methods for solving mesoscopic transport models include FEM, FVM, and LBM, the latter of which solves transport equations based on MD theory. The lattice is uniquely determined by the dimension number D and speed number Q. The commonly used LBM solution scheme for 2D geometry, is D2Q9, while the LBM schemes D3Q7, D3Q15, D3Q19, and D3Q27 are commonly used for 3D geometry.^329^ Selection of the correct microstructural reconstruction technique and method for solving the transport model facilitates investigations of the transport and electrochemical performance of porous electrodes.

As early as approximately 20 years ago, 3D reconstruction of SOFC electrodes was reported in the literature. Early efforts primarily focused on reconstructing and restoring the mesoscopic morphology of real electrodes, as well as extracting and analyzing the relevant parameters of electrode structures. Owing to computer technology limitations at the time, multiphysics field models based on 3D reconstructed structures were not yet established. For example, as early as 2006, James et al.^331^ employed FIB-SEM to carry out 3D reconstruction of a real anode’s structure, and the TPB density and tortuosity were analyzed and calculated. In 2007, Ji et al.^332^ numerically reconstructed the 3D structure of an SOFC cathode using the Monte Carlo method, and loaded the equations governing mass/charge transfers and electrochemical reaction kinetics onto the reconstructed structure. The authors successfully obtained the species concentration and potential distribution of heterogeneous electrodes. Unfortunately, the electrode structure reconstructed using the Monte Carlo method was quite different from the actual electrode’s structure. Since 2010, heterogeneous multiphysics field models based on SOFC electrode reconstruction have rapidly developed.^333^ Nowadays, such models have emerged as vital tools for gaining insight into the mesoscopic electrode structure and reaction-transport mechanisms. The latest SOFC simulation studies based on microstructural reconstruction are reviewed herein.

Brus et al.^334^ developed a 1D homogeneous MPM of a CH_4_-fueled button cell to investigate the distributions of electrical potential, species concentrations, and chemical reaction rates with cell thickness. The microstructures of the AFL, cathode functional layer (CFL), and cathode collector layer (CCL) were reconstructed via FIB-SEM. Parameters such as porosity, particle radius, tortuosity factor, and TPB density were extracted to construct the whole-cell model. Mozdzierz et al.^335^ developed a 2D whole-cell homogeneous model based on the reconstructed electrode microstructure. The authors obtained electrode parameters by processing the microstructure and then incorporated them into the macroscopic MPM. The model provided insight into electrochemical performance, species concentration distribution, and temperature distribution. Additionally, the authors substituted microscopic parameters from the literature for those obtained from the actual electrode structure. This substitution revealed differences between the model results using the literature-based parameters and experimental results, highlighting the substantial impact of the microstructure on cell performance and underscoring the importance of extracting parameters from actual microstructures for accurate prediction and analysis.

Wu et al.^336^ proposed an enhanced kinetic Monte Carlo (KMC) model to simulate the sintering process of a mixed ionic-electronic conducting cathode. The evolution of key microstructure parameters during sintering, such as average particle size, pore size, porosity, tortuosity, effective surface area, and effective TPB, was explored. The results indicated that large tortuosity and small porosity are beneficial to the formation of an effective surface reaction area but are not conducive to gas transport. Wu et al.^337^ analyzed the initial electrode morphology corresponding to the optimal electrode performance through simulation of the electrode sintering and operation process, which was helpful to guiding electrode structure design and optimization.

**Figure 49:**
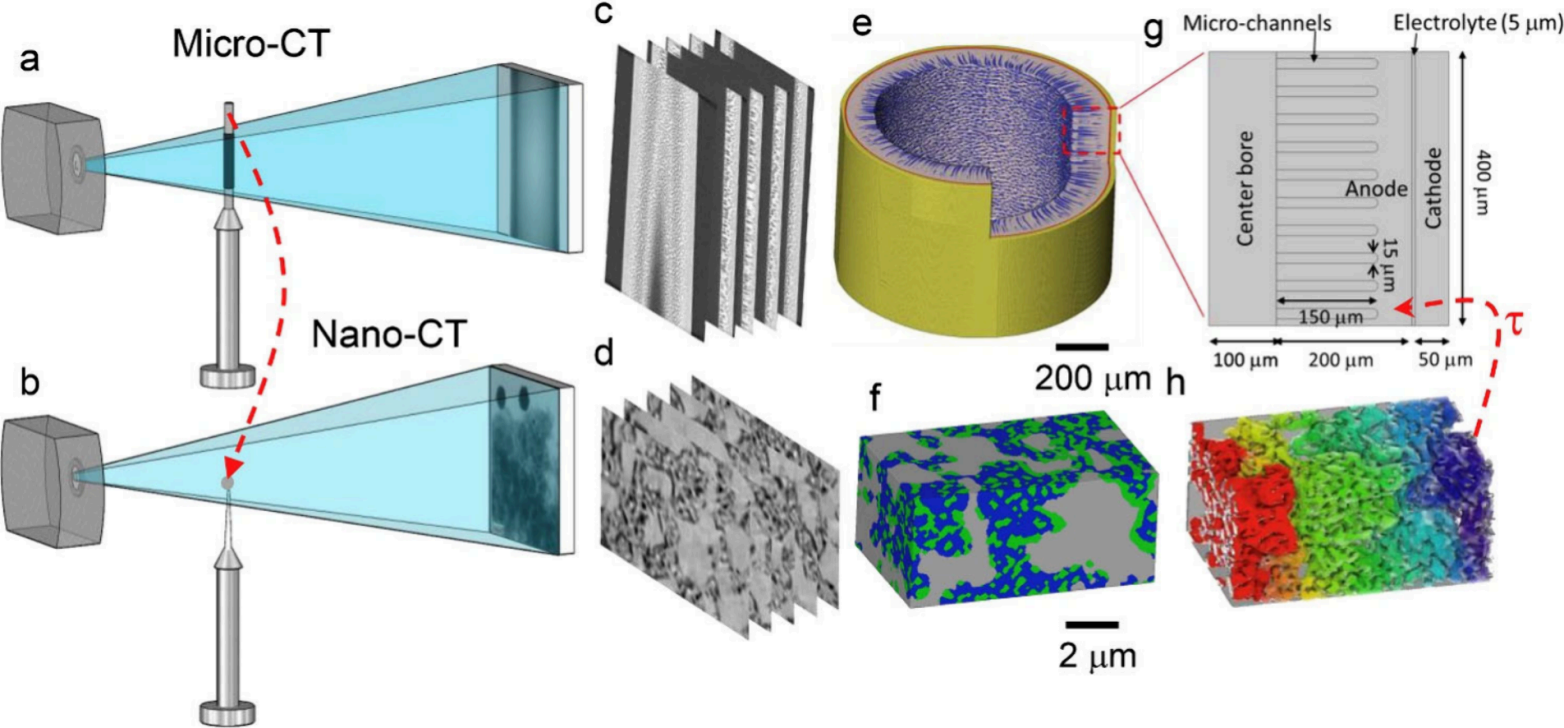
Schematic of (a) micro-CT set-up and (b) nano-CT set-up; (c) reconstructed slices of the SOFC; (d) spongy anode substrate; (e) 3D volume rendering of the segmented micro channels SOFC; (f) 3D volume rendering of the segmented spongy anode; (g) SOFC with finger-like microchannels; (h) concentration gradient of the diffusion simulation on the pore phase of the segmented volume in (f). Reproduced with permission from ref (339). Copyright 2019 Elsevier.

Lu et al.^311^ developed a novel hierarchical tubular SOFC anode with self-organizing microchannels and a sponge-like substrate structure to reduce concentration loss. Given that the pore sizes of the substrate and microchannel structures differ, a hierarchical multiscale approach was employed to image the substrate and all-anode microstructures at different resolutions. Subsequently, the effective transport parameters of the all-anode and sponge substrate were determined. The study also conducted a parametric analysis to explore the relationship between tortuosity, permeability, and substrate porosity. On the basis of their previous research, Lu et al.^338^ further characterized the effective mass transport parameters of a hierarchically porous anode by integrating CFD simulations with multiscale 3D X-ray microstructural reconstruction. These parameters were then used in whole-cell simulations to compare the performance of a conventional tubular SOFC with that of a tubular SOFC with microchannels. The simulation results demonstrated a 70% reduction in concentration polarization and 60% increase in power density for the tubular SOFC with microchannels compared to the conventional tubular SOFC. One of the main technical challenges of this study was finding a balance between the imaging resolution and field of view, thereby increasing the resolution while narrowing the field of view to recognize the 3D microstructure of the substrate, and decreasing the resolution while increasing the field of view to recognize the microchannel structure. Lu et al.^339^ reconstructed the entire cell structure, including the microchannels and anode substrate, using X-ray Micro CT and X-ray Nano CT, respectively. The reconstruction process is detailed in Figure 49. Subsequently, Avizo software was utilized to extract the tortuosity factor and effective mass transfer coefficient for continuous flow of the substrate, and the Knudsen mass transfer coefficients were obtained using the molecular-based direct simulation Monte Carlo method. These parameters were integrated into a 2D whole-cell model, enabling the analysis of various microscopic parameters and microchannel characteristics on cell performance.

**Figure 50:**
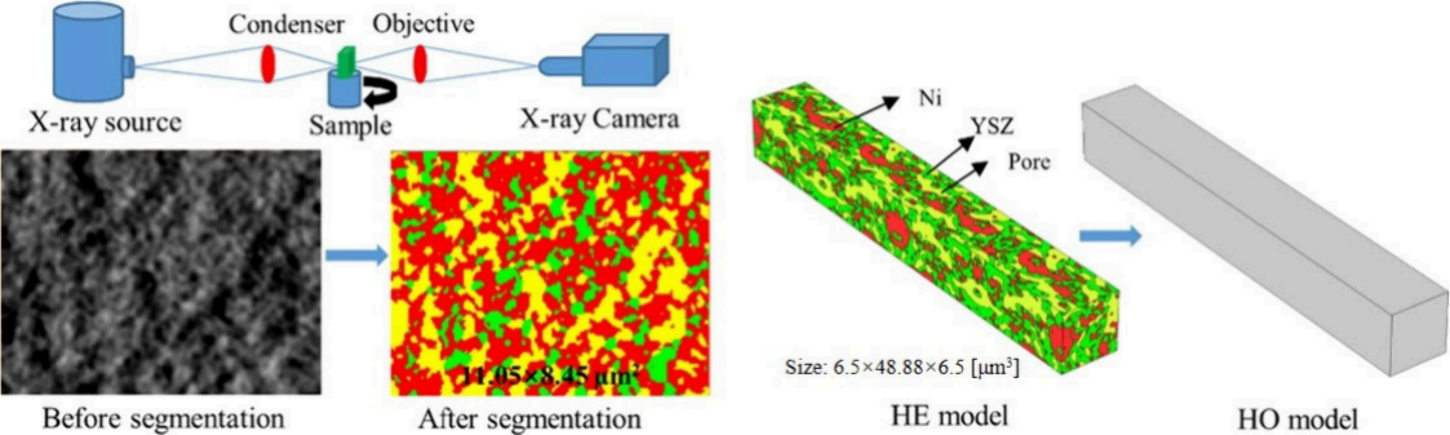
Structural reconstruction process and obtained microscopic porous electrodes. Reproduced with permission from ref (35). Copyright 2021 Elsevier.

Li et al.^35^ reconstructed the anode microstructure and developed an anode heterogeneous model coupled with internal reforming reactions. The porous anode was sliced and scanned via X-ray, yielding 240 images, which were reconstructed using Avizo software into a 3D microstructure. The structural reconstruction process and obtained microscopic porous electrodes are depicted in Figure 50. Owing to the high cost and time-consuming nature of microstructural reconstruction and heterogeneous model solving, only the performance of porous anodes was investigated. The key parameters employed in the homogeneous model, such as TPB density, Ni pore specific surface area, and average particle diameter, were obtained through further processing of the electrode microstructure. Subsequently, the results of the heterogeneous model were compared with those of the homogeneous model, revealing that the total overvoltage of the two models was nearly identical at medium and low current density, while the overvoltage of the heterogeneous model slightly exceeded that of the homogeneous model at high current density.

Zhao et al.^340^ utilized the open-source platform OpenFOAM to develop SOFC MPMs at single-cell and pore scales. The study demonstrated that the OpenFOAM and Fluent models accurately predicted the performance of cell-scale SOFCs, while the OpenFOAM and LBM models effectively predicted the performance of pore-scale SOFCs. The decision to use OpenFOAM was motivated by its open-source nature, which allows for better secondary custom development of multiscale models compared to commercial software. Zhao et al.^340^ reconstructed the microstructure obtained by Xe FIB-SEM, selecting 1243 voxels as the computational domain to establish an anode heterogeneous model. The authors conducted a detailed analysis of the mass/charge transfers and electrochemical reaction processes inside the electrode. An important contribution of their study was the release of the OpenFOAM source code, which may greatly advance the development of SOFC heterogeneous models.

**Figure 51:**
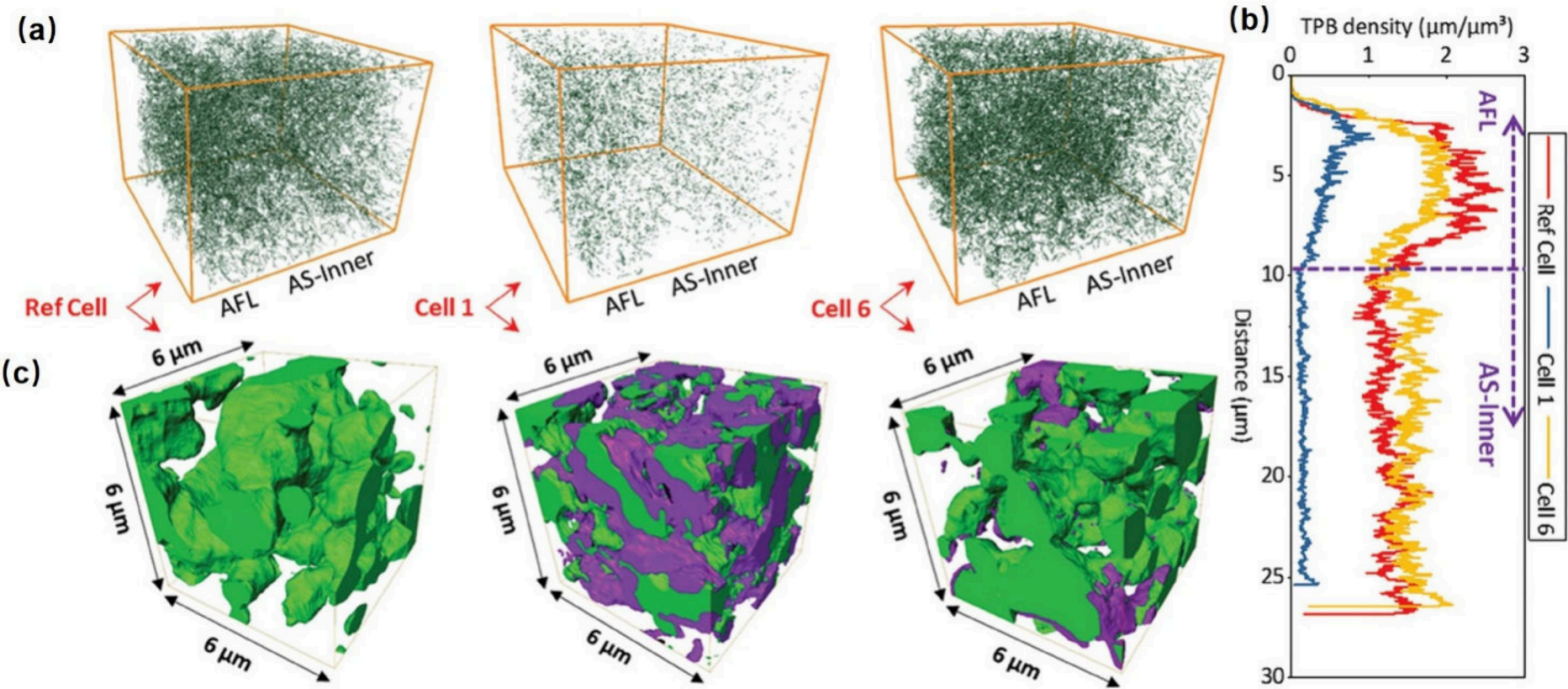
(a) The spatial distribution of anode TPB density (b) the statistical distribution of TPB density along the direction of anode thickness; (c) anode 3D reconstruction structure. Green represents the Ni phase and purple represents the carbon phase. ref cell has only undergone anode reduction and initial conditions; Cell 1 has the S/C of 0 and Cell 6 has the S/C of 1. AS-Inner indicates the inner side of the anode support layer. Reproduced with permission from ref (341). Copyright 2024 Wiley.

In order to explore the mechanisms and microstructural impacts of carbon deposition within the direct internal reforming SOFC using CH_4_ and H_2_O as fuel, Lyu et al.^341^ used FIB-SEM to reconstruct the anode structure at different S/C ratios, as shown in Figure 51. After the microstructure reconstruction, the TPB density of the anode at different S/C was extracted. It can be found that under the condition of small S/C, the carbon deposition is more obvious, and the TPB density is smaller.

Wu et al.^342^ employed the KMC method to reconstruct the microstructure of a mixed ionic-electronic conducting anode. The authors validated the reconstructed structure by comparing the relative density and specific surface area obtained experimentally. Subsequently, the authors solved the heterogeneous MPM of the cathode using pore-scale LBM to investigate the effect of local O_2_ partial pressure and the sintering process on cathode electrochemical performance. The results indicated that the local O_2_ partial pressure significantly increased the LSFC cathode ohmic overpotential when *P*_O_2__ < 0.1. Moreover, the study reported that a small initial grain size and irregular particles led to a large reaction area after sintering, thereby reducing the activation overpotential. The findings of the study provide guidance for the high-performance operation of cathodes and preparation of cathode structures with good morphology.

Li et al.^343^ synthesized four random anode microstructures with gradually increasing particle diameters using the log-normal distribution theory. Based on these structures, the authors synthesized four unidirectional anode microstructures, established a heterogeneous MPM for the eight anode microstructures, and compared their electrochemical performance. The results indicated that the unidirectional microstructure formed by fine particles (Ni particle diameter <0.7 μm) could improve electrode performance by >20% at an operating temperature of 800 °C. Although the TPB density of the random microstructures was large and the electrochemical reaction sites were sufficient, the electrochemical performance was controlled by ohmic loss. By contrast, the unidirectional microstructures had lower tortuosity and ohmic loss, resulting in better electrochemical performance.

**Figure 52:**
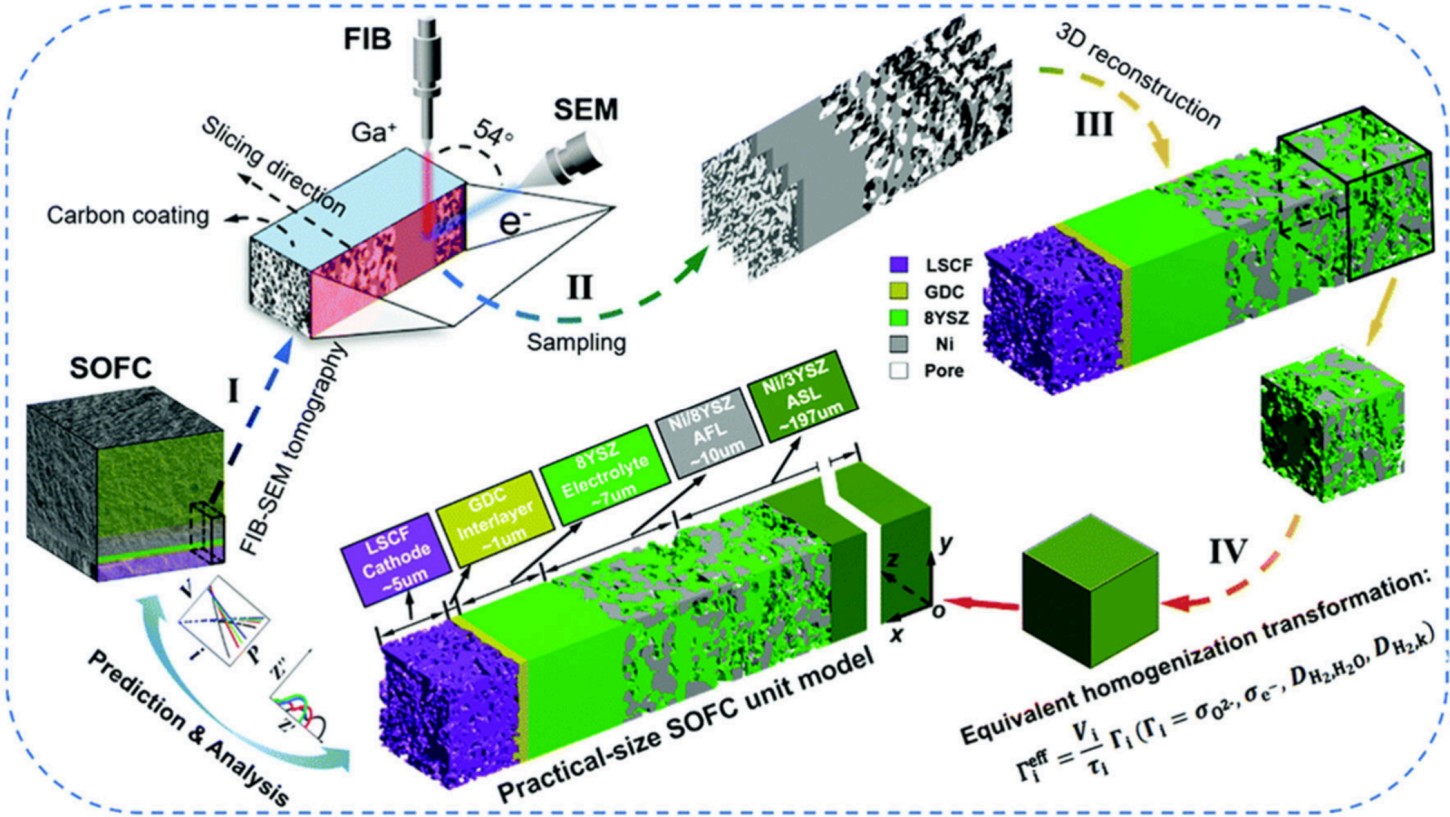
Schematic of the microstructural reconstruction process of a single cell. Reproduced with permission from ref (344). Copyright 2022 RSC.

Su et al.^344^ developed a 3D heterogeneous transient MPM of the whole cell and reconstructed the microstructure via FIB-SEM, as illustrated in Figure 52. Owing to the thickness of the ASL being approximately 197 μm, accurately reconstruct the entire AFL via FIB-SEM was not feasible. Instead, only the ASL with a thickness of 5 μm was reconstructed, and the remaining region of the ASL was modeled homogeneously using relevant microscopic parameters extracted from the previous microstructure. This approach helped to reduce the model’s computational burden. The model was subsequently employed to analyze the polarization characteristics under different conditions and quantify the effect of microstructural evolution, such as Ni particle coarsening and agglomeration, on cell performance. Jiao et al.^345^ employed FIB-SEM to quantify the characteristics of SOFC anodes after degradation, including the microscopic morphology, TPB density, etc.

The above-mentioned cell/electrode multiphysics simulation studies based on microstructural reconstruction are summarized in Table 15, demonstrating three main purposes for electrode microstructural reconstruction:

(1) Visualizing the microstructure and extracting the important parameters affecting cell performance.^35,311,334,335^ Most previous MPMs required an average parameter to quantify electrode performance, such as TPB density and permeability, which was difficult to obtain from macroscale simulations. Even the BRFS model requires providing the diameter or radius of electrode particles to estimate microscopic parameters. Moreover, the BRFS model is only an idealized model and cannot obtain real electrode parameters. Microstructural reconstruction provides a feasible method for accurately obtaining the microscopic parameters of the electrode.
(2) Describing the transport and reaction mechanisms with complex electrode or cell microstructures.^35,339,342,344^ This is particularly relevant for heterogeneous MPMs.
(3) Visually predicting or analyzing the electrode microstructure under the influence of degradation factors, and exploring the electrochemical degradation performance of the electrode/cell.^345,346^ The degradation mechanism based on microstructural reconstruction is more accurate and reliable than that described in Section 3.2.2. In this context, the electrode is directly reconstructed to describe the evolution of the electrode structure under the effect of degradation, providing a more realistic reflection of the electrode structure in a degraded state. By contrast, the degradation equations proposed in Section 3.2.2 are semiempirical and semitheoretical, providing reasonable fits to experimental data but may not accurately describe the evolution of microscopic parameters. From the perspective of MPMs, heterogeneous models are more detailed than homogeneous models, better reflecting the mapping relationship between microstructure and macroscopic electrochemical performance. However, heterogeneous models incur higher computational loads and longer computation times. Consequently, heterogeneous models tend to be used for analyzing electrode performance rather than whole-cell performance. At present, some scholars have begun to employ supercomputers to solve multiscale models, enabling comprehensive and careful study of SOFCs. For example, Tim et al.^347,348^ constructed a 10 × 10 × 10 μm^3^ heterogeneous-physics model of the cathode–electrolyte subvolume. The model was solved using two supercomputers, Joule at the National Energy Technology Laboratory and Bridges at the Pittsburgh Supercomputing Center in the U.S. The computation time for each case simulation was approximately 30 min to 2 h. Although supercomputers can greatly improve the computational efficiency of heterogeneous models, reduce their running time, and expand their size, the adopting of supercomputers requires the support of powerful laboratories.

**Table 15:** Summary of Whole-Cell/Electrode Multi-Physics Simulation Studies Based on Microstructural Reconstruction

authors	cell type	fuel type	transport equation	reconstruction method	solution method	main concerns	ref
Li et al.	3D anode	Prereforming - gas	Mass, Charge	X-ray	FEM (COMSOL)	• Microscopic parameter extraction;	(35)
						• Heterogeneous and homogeneous model comparison;	
						• Parametric analysis on heterogeneous model.	
Lu et al.	3D anode	H_2_	Heat, Momentum	X-ray CT	FVM (STAR CCM+)	• Enhanced mass transfer through microchannels;	(311)
						• Hierarchical multiscale method for microscopic reconstruction;	
						• Transport parameter extraction and analysis.	
Lu et al.	3D anode and 2D tubular cell	H_2_	Mass, Charge, Momentum	X-ray CT	FVM (STAR CCM+)	• Solve the trade-off between of imaging resolution and field of view in hierarchical porous materials;	(338)
						• Study the enhancement effect of microchannels on the electrochemical performance of whole microtubular cell.	
Lu et al.	3D anode and 2D tubular cell	H_2_	Mass, Charge, Momentum	X-ray CT	-	• Continuous flow and Knudsen diffusion mass transport parameter extraction;	(339)
						• Simulation of electrochemical performance of microchannel microtubular SOFC with microchannels.	
Brus et al.	1D cell	CH_4_	Mass, Charge. Momentum	FIB-SEM	FVM (MATLAB)	• Extraction of parameters from the microstructure of the electrode;	(334)
						• Construction of 1D button cell homogeneous model.	
Su et al.	3D cell	H_2_	Mass, Charge, Momentum	FIB-SEM	FEM (COMSOL)	• Development of 3D heterogeneous whole-cell model;	(344)
						• Explore polarization characteristics under different conditions;	
						• Quantification of the effect of microstructural evolution on cell performance.	
Mozdzierz et al.	2D cell	H_2_	Mass, Charge, Energy, Momentum	FIB-SEM	FVM (In-house code)	• Extraction of parameters from the microstructure of the electrode;	(335)
						• Confirm that effect of microstructure is not negligible.	
Wang et al.	3D anode	H_2_	Mass, Charge	PFM	FDM (In-house code)	• Evolution of electrode structure in response to Ni particle coarsening;	(349)
						• Effect of Ni particle coarsening on anode overpotential.	
Wu et al.	3D cathode	O_2_	Mass, Charge	KMC	LBM (In-house code)	• Microstructure of the cathode was reconstructed by KMC;	(342)
						• The cathode heterogeneous MPM was solved by LBM.	
Zhao et al.	3D anode	H_2_	Mass, Charge	Xe FIB-SEM	FVM (OpenFOAM)	• The representative element size related to current density was determined;	(340)
						• Open-source heterogeneous model OpenFOAM code.	
Li et al.	3D anode	H_2_	Mass, Charge	Lognormal distribution	FEM (COMSOL)	• The anode microstructure was reconstructed by logarithmic normal distribution;	(343)
						• The unidirectional microstructure was proposed and simulated.	

A previous study indicated that the difference between the results of heterogeneous and homogeneous models for the same electrode was insignificant.^35^ Therefore, selecting the appropriate model according to the study’s purpose is crucial. Regarding the reconstruction approach, the physical experiment method often requires sophisticated instruments and professional image processing software but offers the advantages of being intuitive and straightforward, with the obtained 3D microstructure reflecting the real pore electrode. By contrast, the numerical method does not require sophisticated instruments but necessitates a deep understanding of numerical theory, and the obtained microstructure requires further validation using experimental data.

#### 4.2.3 Application of Emerging Simulation Methods in SOFC Modeling

With the continuous development of numerical simulations, several advanced techniques have been applied in SOFC modeling to address challenges that are difficult to solve via conventional methods. These emerging techniques can effectively reflect the degradation mechanisms or mechanical performance of SOFCs. However, their application in SOFC modeling is currently limited. Among these advanced methods, the phase field model (PFM) and Peridynamics method are notable. This section focuses on the application of these methods in SOFC simulation research.

PFM is a thermodynamics-based model that considers the comprehensive actions of thermodynamic potential and driving force to establish the governing equations that describe system’s evolution dynamics. These governing equations are divided into conservative field and nonconservative field equations, described by the Cahn–Hilliard and Allen–Cahn functions, respectively. For detailed information and governing equations of PFM, readers can refer to refs.^350−353^ Currently, PFM is mainly applied in studying the evolution of electrode microstructure during the operation or degradation processes of SOFCs. By obtaining the electrode microstructure, the model can extract microscopic parameters and simulate electrochemical performance. The degradation simulation is mainly utilized to determine the evolution of the mesoscopic electrode structure during operation. Thus, the governing equations can be loaded onto the heterogeneous structure to directly obtain the evolution of electrode performance. This differs from the degradation model developed in Section 3.2.2. wherein semiempirical and semitheoretical equations are utilized to describe the evolution of relevant parameters in the degradation process. Here, the reliable and fine simulation of degradation performance is achieved by directly capturing the degradation process of the electrode structure. However, predicting the degradation performance of a large cell solely based on an electrode microstructure degradation simulation is challenging owing to the time and computing resources required to solve large heterogeneous models. Therefore, PFM is primarily applied in analyzing degradation mechanisms and visualizing the evolution of microscopic electrode morphology. Upon its acquisition via PFM, a more reliable degradation mechanism can be integrated into a homogeneous multiphysics field model to perform large-scale cell degradation performance simulations and lifetime predictions.

**Figure 53:**
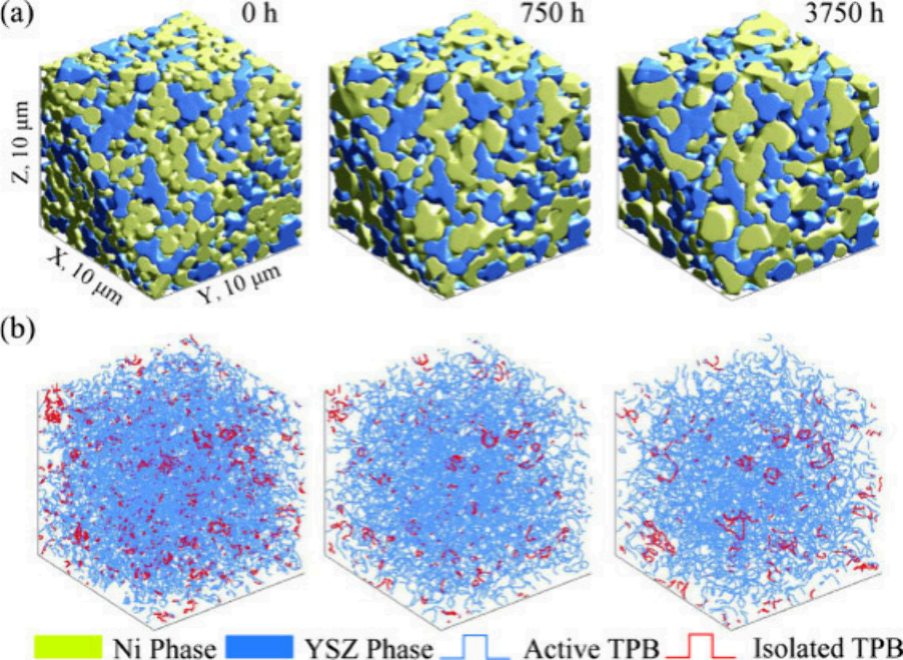
Evolution of (a) anode morphology and (b) TPB network during Ni particle coarsening. Reproduced with permission from ref (353). Copyright 2021 Elsevier.

Wang et al.^353^ utilized PFM to simulate and predict the morphological evolution of the anode microstructure during Ni particle coarsening, as depicted in Figure 53. This figure clearly illustrates that the Ni particle diameter increases over time due to Ni coarsening. Additionally, Wang et al.^353^ validated the reliability of the predicted microscopic morphology by comparing experimental and simulation results of Ni particle diameter, TPB length, and normalized specific interfacial area evolutions. Subsequently, after obtaining the reliable anode microstructure, the authors incorporated the electrochemical and mass transfer governing equations of the anode to develop a heterogeneous MPM. The model is solved by finite difference method using MATLAB. The results indicated that the total overpotential of the electrode increased from 0.176 to 0.191 V after 3750 h due to Ni coarsening at the operating temperature of 850 °C and current density of 4000 A·m^–2^. To maintain the optimal balance between the percolation rate and ionic conductivity, while mitigating Ni coarsening, the authors recommended maintaining the Ni-to-YSZ volume ratio within the range of 0.49–0.68, thereby ensuring the desired electrochemical performance.

**Figure 54:**
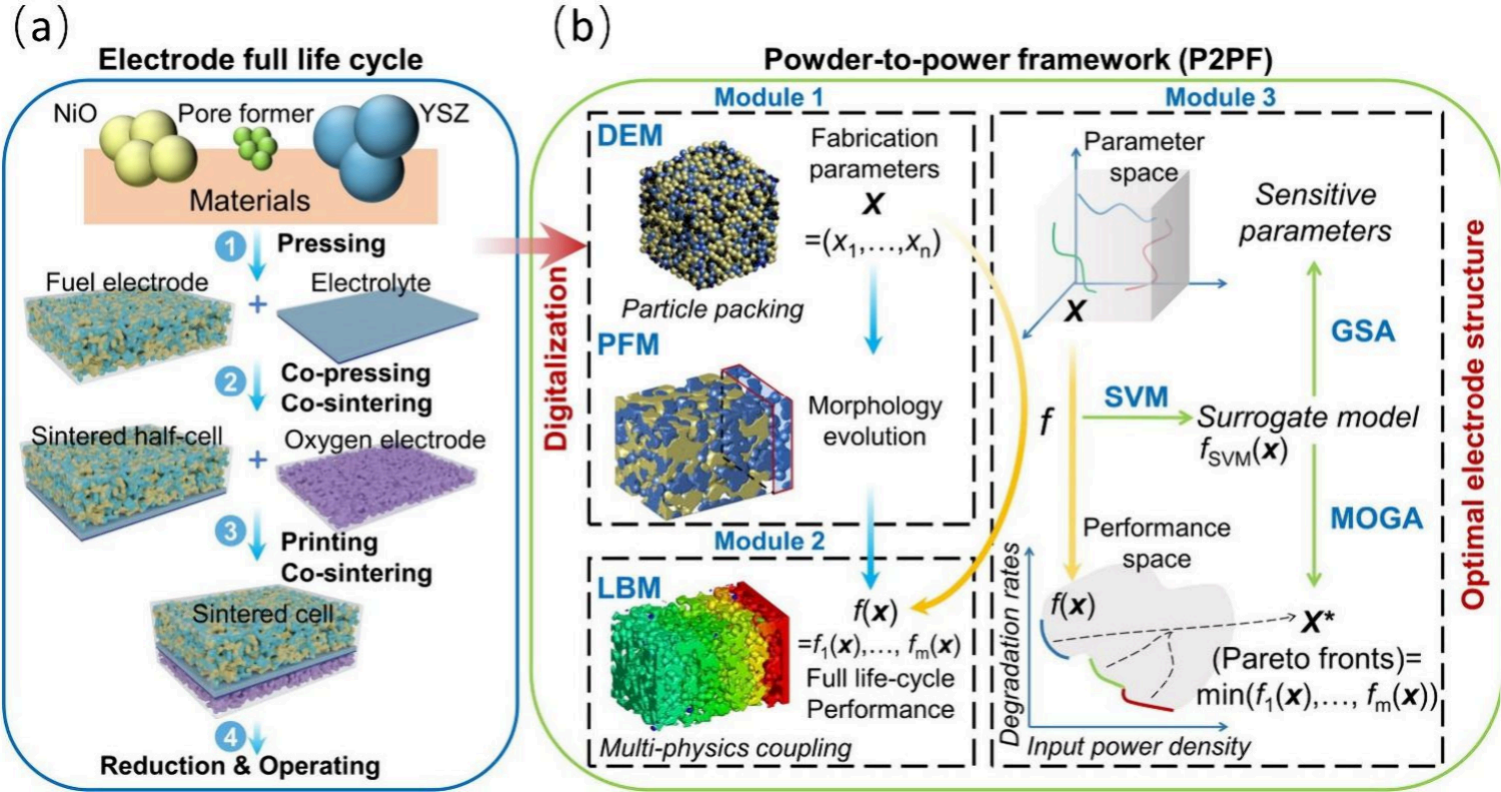
(a) Full life cycle of an SOFC electrode from powder to pressed tablet to sintered cell. (b) schematic illustration of the “powder-to-power” simulation framework comprising three modules: Module 1 uses DEM to simulate the mesoscopic structure of the electrode obtained after powder laminating, then PFM is used to trace the mesoscopic structure of the electrode during sintering, reduction, and long-term operation; Module 2 establishes the heterogeneous multiphysics field model of the electrode using LBM to simulate the life-cycle performance; and Module 3 uses a support vector machine to obtain the surrogate heterogeneous model of the electrode and adopts a multiobjective GA to optimize electrode performance. Reproduced with permission from ref (355). Copyright 2023 Elsevier.

Wang et al.^354^ proposed a mesoscopic electrode reconstruction and simulation framework, named the “powder-to-power” framework, which is depicted in Figure 54. First, the discrete finite element method (DEM), based on a particle-packing algorithm, was employed to digitize the pressed electrode precursor structures composed of NiO, YSZ, and pore former. Then, the digital 3D structure was utilized as the initial computational domain for PFM to simulate the evolution of anode morphology under sintering, reduction, and long-term operation. Finally, the mesoscopic structures obtained by PFM were employed as the computational domain to construct the heterogeneous multiphysics field model of the electrode, which was solved by LBM. This framework achieved the full-process mapping relationship from powder composition (phase volume fraction and particle diameter) to mesoscopic electrode structure and electrochemical performance. Utilizing this framework, the mesoscopic structure and powder composition of electrodes with superior performance can be determined, guiding the preparation of high-performance electrodes and selection of operating conditions. In this study, PFM was also utilized to predict the evolution of electrode structure during sintering, reduction and operation processes.

Jiao et al.^356^ utilized PFM to simulate the evolution process of the anode microstructure under Ni coarsening. The authors introduced a semi-implicit Fourier spectral method into PFM to enhance computational efficiency. Similarly, a reduced anode microstructure was employed as the initial condition. After obtaining the anode microstructure at different times, the authors extracted the Ni-phase specific area, TPB density, and Ni- and pore-phase tortuosity factors as typical microscopic parameters. The reliability of the model was quantitatively determined by comparison with experimental data. Building on this research, Jiao et al.^357^ introduced material composition and crystallographic orientation order parameters to simultaneously simulate Ni crystal particle growth and the corresponding morphological changes. The reduction process of NiO in anodes can impact the initial performance and stability of Ni/YSZ composite anodes in SOFCs, as revealed by Jiao et al.,^352^ who simulated the reduction process of Ni/YSZ anodes using PFM, considering not only the reduction process of NiO but also the sintering mechanism of Ni. The initial anode microstructure was reconstructed via FIB-SEM. The reliability of the model was confirmed by qualitative comparison with the anode microstructure obtained experimentally at different temperatures.

**Figure 55:**
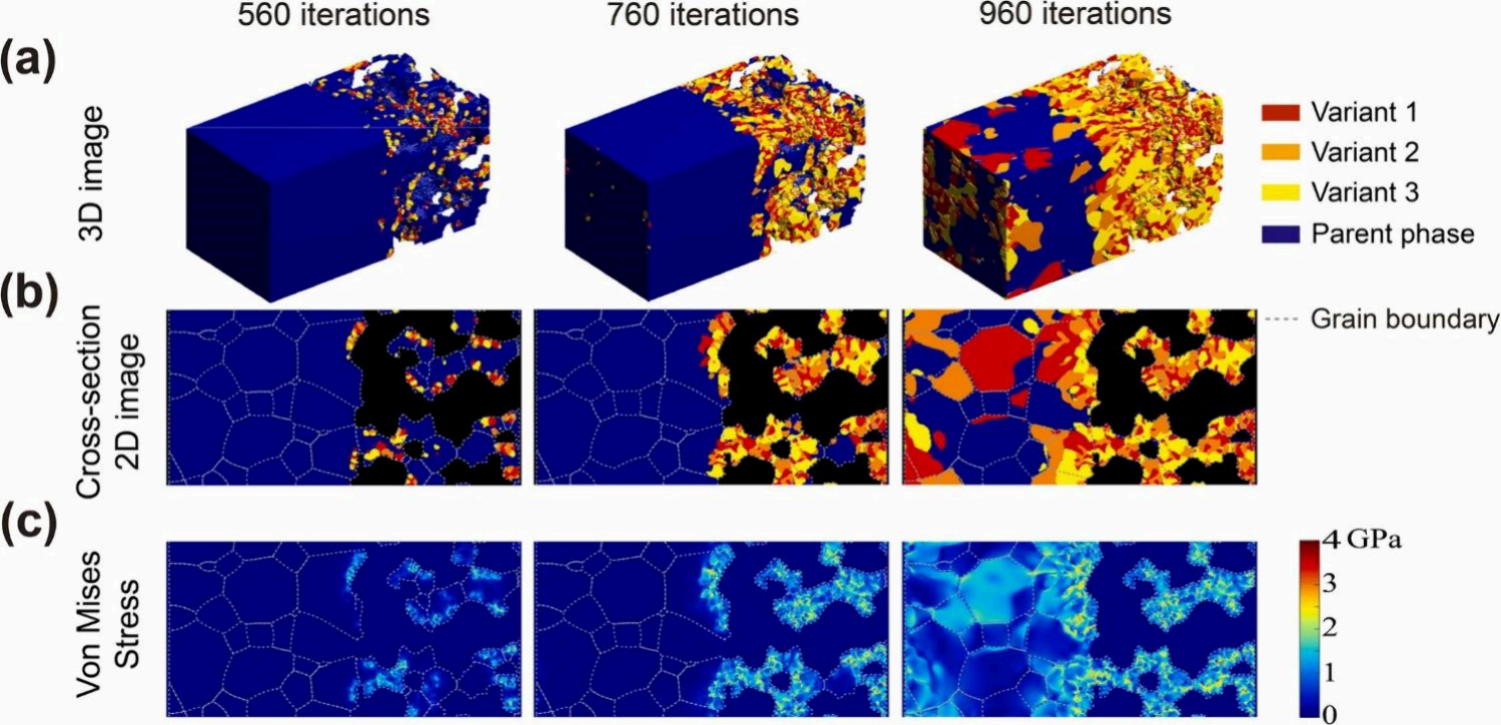
Simulated distributions of variant phases in (a) the 3D polycrystalline YSZ microstructure and (b) 2D cross-section images. (c) the von Mises stress distributions in the 2D cross-section image. Reproduced with permission from ref (358). Copyright 2022 Elsevier.

The electrolyte degradation of YSZ grains caused by the transition from cubic phase to tetragonal phase (c-t) is a key problem leading to SOFC degradation. Da et al.^358^ quantitatively studied the effect of YSZ c-t phase transition on SOFC by establishing a microelastic PFM to simulate the phase transition. In addition, PFM was applied to the 3D microstructural reconstruction of polycrystalline YSZ. Figure 55(a) presents the simulation distribution of the variant phases in 3D microstructures under different iteration steps, revealing that the variant phases gradually increases and spread to the dense electrolyte region. The different variant numbers represent the tetragonal variants along the three axes of the coordinates. Figure 55 demonstrates that high von Mises stress was accumulated within the YSZ skeleton and dense electrolyte, leading to the formation and development of microcracks in the electrolyte region. This study provided an example for the application of PFM to simulations and predictions of phase transitions within the electrode microstructure. To sum up, PFM is a powerful tool for predicting and simulating the microstructural evolution of SOFC electrodes, capable of overcoming the inherent limitations of experimental methods when it comes to capturing microstructural evolution, which is frequently time-consuming and expensive.

Peridynamics is an emerging method for establishing models and describing the mechanical behavior of matter by solving spatial integral equations based on the idea of nonlocal interactions. The basic idea of Peridynamics was initially proposed by Silling in 2000,^359^ and the basic concepts and principles of Peridynamics were introduced in detail in the PhD thesis of Speronello, which provided the model code.^360^ The Peridynamics method is mainly applied to problems such as large deformation, damage, fracture, penetration of homogeneous/heterogeneous materials and structures, crystal phase transformation dynamics, and failure of nanomaterials and structures.^361^ At present, the application of Peridynamics theory in SOFC modeling mainly focuses on visualizing crack distribution, initiation, and propagation, stress distribution, and fracture dynamic results in electrode microstructures.

**Figure 56:**
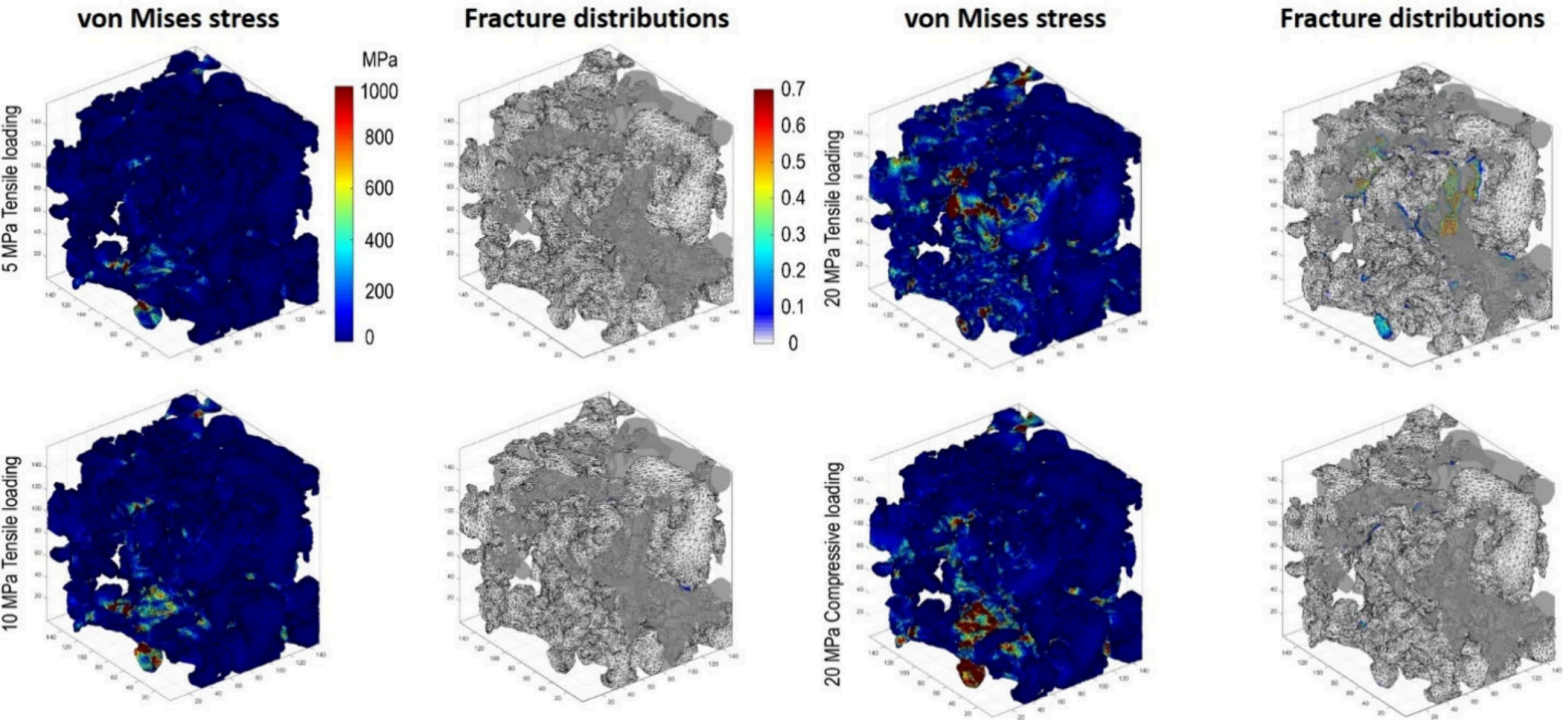
von Mises stress and fracture distributions within the anode microstructure under different external loads. The Ni and YSZ phases are shown as transparent and gray phases, respectively. Reproduced with permission from ref (362). Copyright 2022 Elsevier.

The brittle ceramic phases and metal–ceramic interfaces in SOFC electrodes are prone to cracking, which can lead to mechanical damage and irreversible degradation of cell performance. Xiang et al.^362^ employed FIB-SEM to obtain the anode microstructure and proposed an ordinary state-based Peridynamic model to simulate the formation of cracks in the anode microstructure. Additionally, the effect of external mechanical load on the strength of the anode microstructure was quantitatively assessed. Figure 56 illustrates stress and crack distributions within the anode microstructure under different external loads, revealing that no cracks were present when the external tensile stress was 5 MPa. However, the area covered by high stress gradually expanded when the tensile stress reached 20 MPa, and fracture damage appeared on a large area of the Ni–YSZ interface and at several YSZ locations. The Peridynamic method provides a feasible approach for visualizing stress and crack distributions within the electrode microstructure. Xiang et al.^363^ reconstructed the anode microstructure at different sintering temperatures and proposed a weakly coupled thermal-mechanical Peridynamics model to predict mechanical damage under severe thermal shock. Wang et al.^364^ adopted the Peridynamics method to calculate the deformation and stress of a 2D anode microstructure under uniform thermal load and analyze the process of crack formation and propagation therein.

In summary, these new simulation methods have emerged to solve specific problems that are challenging to address via conventional simulation technology. On the one hand, PFM is primarily utilized to predict and simulate the evolution of electrode microstructures during operation or degradation processes, such as during NiO reduction,^352,355^ Ni particle coarsening,^351,353,356,357,365,366^ and YSZ phase transformation.^358^ On the other hand, the Peridynamics method is mainly employed to address mechanical problems related to crack generation, growth, and diffusion in electrode microstructures. These problems are difficult or even impossible to address using conventional MPMs. The emergence of these simulation methods provides new solutions to these challenges. Importantly, studies have demonstrated the reliability and effectiveness of PFM and Peridynamics in addressing these problems. However, the implementation of these emerging simulation technologies requires a solid theoretical foundation and programming ability given that mature and user-friendly software has not yet been developed, highlighting an area that requires further breakthroughs and in-depth exploration.

### 4.3 Challenges and Prospects of Mesoscopic Models

Summarizing the above-mentioned studies reveals that the most mesoscopic models have been developed from cell/stack models from a technical point of view. For example, elementary reaction models are developed by changing the reforming and WGS global chemical reaction kinetics to elementary reaction kinetics in traditional MPMs. Heterogeneous multiphysics field models are constructed by replacing the homogeneous electrode geometry with a more realistic heterogeneous electrode structure. Hence, mesoscopic models can obviously obtain more fine and rich information than cell/stack models. This is also the point of mesoscopic models, which are adopted to solve problems that cell/stack models have difficultly describing. Inevitably, the shortcomings of mesoscopic models are also obvious, which include being more difficult to establish and more complex to solve. Moreover, the development of each mesoscopic model requires breaking through the corresponding technical difficulties. For example, elementary reaction models can only be established by accurately constructing multiple elementary reaction kinetics and reasonably describing the mass transfer processes of multiple surface and bulk species. Given that the elementary reaction kinetics involve many surface and bulk species, the number of variables in the model that need to be solved greatly increase. To successfully address heterogeneous models using either FEM or FVM, precisely identifying the phases and their interfaces is imperative while also ensuring a reasonable mesh of the electrode structure. Other novel mesoscopic models, such as PFM and the Peridynamics model, require unique and advanced techniques to be realized.

The above analysis clearly indicates the challenges and prospects of mesoscopic models. Mesoscopic models can describe or solve the problems encountered by SOFCs at the mesoscopic level, such as reaction path exploration, electrode structure optimization, degradation mechanism, electrode crack distribution, etc. These unique applications highlight that the mesoscopic model has an important position in future SOFC fine research. However, considerable challenges face mesoscopic models. That is, mesoscopic models are difficult to construct and solve, requiring deep modeling experience and interdisciplinary knowledge. For example, it is time-consuming and costly to generate three-dimensional electrode structures by experimental means, so the researchers can try to use the image generation method in AI to generate realistic electrode structures.

## 5 Analysis and Selection Guide of Multiscale Solid Oxide Cells Model

### 5.1 Development of SOFC Cross-Scale Models

As previously mentioned, SOFC models at each scale target their own problems. When a problem becomes intractable at a particular scale, establishing a cross-scale model becomes essential. Notably, existing studies have different understandings of the term “cross-scale”. Mastropasqua et al.^367^ developed a cross-scale model and applied it to an axially graded electrode design. In their study, the mass and heat transfer processes in the PEN were described by a microscopic model, while the mass/momentum/heat transfer processes in the flow channel were described by a macroscopic model. In fact, the reaction-transfer processes in the PEN and flow channel were described by a macroscopic cell/stack model. Amiri et al.^368^ proposed a SOFC cross-scale modeling framework, including compartment-scale (1 μm–1 cm), channel-scale (1–10 cm), cell-scale (1–10 cm), stack-scale (10 cm–1 m), and system-scale (1–10 m). However, this study failed to provide a mathematical description of the models at each scale, nor did it offer a typical and detailed example of a cross-scale coupled model. In this study, the compartment-scale model was classified as a mesoscopic model and the channel, cell, and stack-scale models were classified as a cell/stack model. Therefore, the cross-scale model in this study included at least two out of the three types of models: mesoscopic, cell/stack, and system models. Models at each scale have specific construction methods and solution strategies, making the development of a cross-scale model challenging. Development of a cross-scale model requires converting single-scale models and establishing interfaces between models of various scales. This is one of the reasons why there are currently few SOFC cross-scale models.

**Figure 57:**
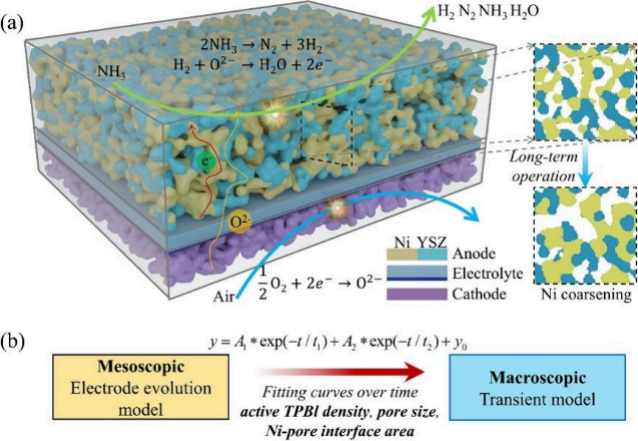
(a) Diagram of PEN structure, reaction process, and Ni coarsening process of direct NH_3_-fed SOFC. (b) coupling relationship between mesoscopic electrode evolution model and macroscopic single-channel cell transient degradation model. Reproduced with permission from ref (369). Copyright 2024 Elsevier.

Wang et al.^369^ proposed a cross-scale model framework that connected meso- and macroscopic models, as shown in Figure 57. First, PFM was employed to simulate the electrode morphology evolution under the influence of Ni particle coarsening. After obtaining the electrode morphology at different times, the function of key mesoscopic parameters, such as the TPB density, Ni specific surface area, and average pore diameter, was fitted with time. Then, the single-channel 3D MPM coupled with the evolution of mesoscopic parameters was established to obtain the electrochemical degradation performance. This study coupled a mesoscopic electrode model and single-channel cell model. The function of key parameters change over time was employed as the interface of the models at two different scales. Similarly, Su et al.^344^ established a cross-scale model coupling a mesoscopic electrode model and macroscopic single-cell model. As shown in Figure 52, carrying out a 3D reconstruction of the ASL entire structure was impossible because the ASL thickness was too large. Even if the entire 3D reconstruction of the ASL was acquired by mirroring the existing electrode structure, the computational costs would be too high. To solve this problem, Su et al.^344^ proposed that, in the process of single-cell multiphysics field model construction, part of the ASL adopted a macroscopic homogeneous structure and the other regions adopted a mesoscopic heterogeneous structure. The coupling of two models at different scales was realized through the interaction of heterogeneous and homogeneous structure interface data. Establishing such a multiscale model enabled fine mesoscopic simulation of the cell while reducing computational and structural reconstruction costs.

Relatively few studies have reported SOFC cross-scale numerical simulations to date. However, it is worth noting that cross-scale models provide more information than single-scale models and provide new solutions for solving problems that are currently difficult for single-scale models to solve. The key to constructing a multiscale model is defining the interface of each single-scale model to realize their information interaction. Needless to say, the process of solving and establishing cross-scale models will be more complex and difficult than that of single-scale models. Nevertheless, complex cross-scale problems will continue to emerge as SOFC-related research deepens, promoting multiscale model development. Cross-scale model will be the next research focus in SOFC modeling.

### 5.2 Modeling of Reversible Solid Oxide Cells

**Figure 58:**
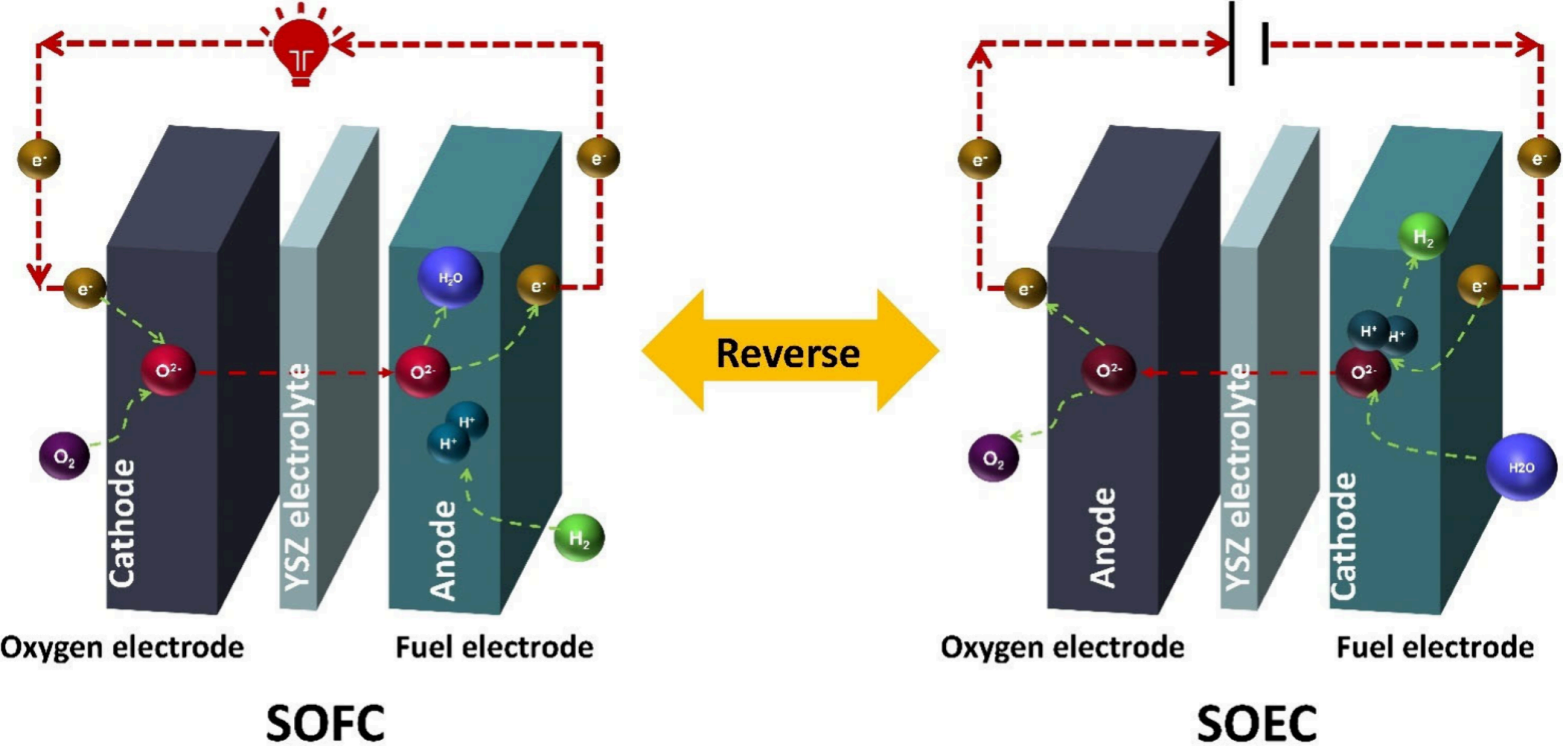
Comparison between the working processes of SOFCs and SOECs.

The working process of SOECs is reversed from that of SOFCs. SOECs are mainly utilized to electrolyze H_2_O/CO_2_ at high temperatures to produce H_2_/CO fuel. A comparison between the working processes of SOFCs and SOECs is illustrated in Figure 58. In SOECs, H_2_O or CO_2_ is supplied to the cathode, where gains electrons to form H_2_/CO and O^2–^. O^2–^ is then transported through the dense electrolyte to the porous anode, where O^2–^ loses electrons to form O_2_. The specific electrode reactions are expressed by eqs 92–94. The electrode material, structure, and composition of SOECs and SOFCs are similar, consequently making their governing equations for charge/mass/momentum transfers, and energy conservation similar, as expressed in eqs 37–46, eqs 49–58, and eqs 63–65. Notably, the source terms in the governing equations for SOECs differ from those SOFCs. For example, H_2_O/CO_2_ is consumed as a reactant in the mass transfer equations for SOECs, and the source term should be negative. By contrast, H_2_O/CO_2_ is generated in the mass transfer equations of SOFCs, and the source term should be positive. These subtle differences require special attention during modeling.
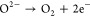
92
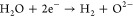
93
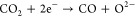
94

The operating voltages of SOECs and SOFCs also differ. The operating voltage of SOFCs is lower than the Nernst voltage, while the opposite is true for SOECs owing to polarization loss. Specifically, the operating voltage of SOFCs is equal to the Nernst voltage minus the polarization loss, as expressed by eq 68. Conversely, the operating voltage of SOECs is equal to the Nernst voltage plus the polarization loss, as expressed by eq 95.^370^
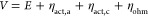
95

The heat released during the working processes of SOFCs and SOECs can be described by eq 96 without considering heat dissipation. The thermal effects of SOFCs and SOECs also differ. The electrochemical reactions in SOFCs are exothermic, which tends to increase the cell temperature, while the electrolysis reactions in SOECs are endothermic, which tends to decrease the cell temperature. The overpotential loss in SOFCs and SOECs tends to increase the cell temperature. Therefore, the electrochemical reactions and overpotential loss in SOFCs always increase the temperature of the cell. However, the overall thermal effects in SOECs are more complicated. The heat demand for electrolysis reactions linearly depends on the current density, while the heat generation due to overpotential loss is nonlinear with current density. Therefore, heat demand is higher than heat generation when the voltage is small, and the SOEC is in endothermic operation mode. When the heat demand for electrolysis reactions is equal to the heat generation due to overpotential loss, the corresponding working voltage is called thermal neutral voltage (TNV). When voltage > TNV, the heat generation due to overpotential loss is higher than the heat demand for electrolysis reactions, and the SOEC is in exothermic operation mode.
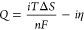
96where *Q* is the thermal power (*Q* < 0 in exothermic state; *Q* > 0 in endothermic state); *i* is the current density (*i* > 0 in SOFC mode; *i* < 0 in SOEC mode); η is the polarization voltage; and Δ*S* is the entropy change (Δ*S* < 0 in SOFC mode; Δ*S* > 0 in SOEC mode).

In general, the working processes of SOECs and SOFCs are completely opposite; however, their governing equations are the same. When modeling SOECs, the species, charge, and energy source terms must be modified based on the SOFC model. In addition, when considering SOC degradation, it should be noted that Ni migration is an important factor affecting SOFC durability, while Ni coarsening is an important factor affecting SOFC. Since the working mechanism of SOEC and SOFC is opposite, the factors affecting the durability of these two are also different. This point should be emphatically confirmed in the study of degradation performance. Several reviews have been published on SOEC modeling, providing detailed information about SOEC models.^371,372^

### 5.3 Application-Oriented Comparison of MPMs at Different Scales

In summary, mesoscopic models have been discussed, including MPMs based on mesoscopic elemental reactions, MPMs based on microstructural reconstruction, and other emerging models. Elemental reactions are primarily employed to uncover chemical/electrochemical reaction mechanisms, while microstructural reconstruction techniques are adopted to visualize the microstructure and extract microscopic parameters. Other emerging models are primarily designed to solve a specific problem. For example, PFM is mainly utilized to predict the mesoscopic structure of the electrode, while the Peridynamics method is mainly employed to solve the mechanical problems related to electrode damage. Studies in Sections 4.1 and 4.2 demonstrated that the prediction results of MPMs based on elemental reactions or microstructural reconstruction and homogeneous macroscopic models for the same cell do not significantly differ.^35,321^ While MPMs coupled with mesoscopic mechanisms are more refined and accurate than macroscopic models for simulating the same cell or electrode, they are also more complex and time-consuming, making them unsuitable for all research subjects. This highlights the importance of selecting an appropriate model. To guide the selection of the appropriate MPM for a given research subject or target, the application scenarios and features of MPMs in Sections 3 and 4 have been compared in Table 16, providing a clear overview of the functions, advantages, disadvantages, and application objectives of various MPMs. With the help of Table 16, researchers can easily select the appropriate model for their specific problem to be solved.

**Table 16:** Application-Oriented Comparison of SOFC MPMs at Different Scales

model type	functions	advantages	disadvantages	applications
Macroscopic models				
2D/3D single cell/stack MPM	• Analysis of reaction-transfer phenomena in whole cell;	• Mature model and simulation software;	• Relies on reliable microscopic electrode parameters;	• Prediction of performance of whole cell;
	• Structure optimization and stress simulation;	• Strong engineering practicability.	• Hard to visualize the microstructure.	• Optimization of cell structure;
	• Dynamic response and full cell degradation life prediction.			• Dynamic response and degradation life prediction.
Mesoscopic models				
MPM based on elementary reactions	• Further investigating the reaction mechanism accurately;	• Compensates for the lack of or some reaction kinetics;	• Difficult to solve;	• Exploration of the reaction mechanism with SOFCs;
	• Exploring the effect of chemical/electrochemical reaction mechanisms on cell performance.	• Reaction kinetics are more accurate.	• Difficult to apply to 3D model;	• Studying the elemental reaction mechanism of carbon deposition.
			• Long simulation time.	
Homogenous MPM	• Extraction of microscopic parameters;	• Electrode structure parameters are more accurate.	• Precise instruments or complex reconstruction methods.	• Requirements for the electrode parameters;
	• Visualization of electrode structure evolution.			• Electrode structure degradation characterization and performance simulation.
Heterogeneous MPM	• Studying the reaction-transfer mechanism of complex electrode microstructures.	• Precisely describes the electrode working mechanism;	• Precise instruments or complex reconstruction methods;	• Studying and visualizing the of electrode working process.
		• Visualizes electrode performance.	• Long simulation time.	
PFM	• Predicting and simulating the evolution of the electrode microstructure.	• Capturing the evolution of the microstructure becomes feasible and convenient.	• Complex theoretical basis and cumbersome programming;	• Capturing the evolution of the electrode microstructure during the degradation process.
			• Difficult to describe the degradation of the whole cell.	
Peridynamics	• Simulating the mechanical performance of the electrode microstructure.	• Simulating the mechanical performance of the microstructure becomes feasible and convenient.	• Complex theoretical basis and cumbersome programming;	• Simulating large deformation, damage, fracture, and impact of microstructure.
			• Difficult to describe the mechanical performance of the whole cell.	

## 6 Conclusions and Recommendations

Numerical simulations play a crucial role in studying the working mechanisms of SOFCs and guiding their design and operation. To this end, numerous models have been developed to address various challenges associated with SOFC manufacturing and operation. This review provides a comprehensive summary and analysis of a large body of research on SOFC simulations, categorizing the developed models according to different scales. SOFCs are inherently complex electrochemical energy conversion devices, ranging from the scale of electrode materials to porous electrodes to single cells/stacks to entire systems. The complexity of the SOFC process results in various challenges across these different scales. Therefore, a comprehensive understanding and resolution of scale-specific challenges are essential for achieving efficient and stable power generation in SOFCs. This article conducts a detailed top–down review of SOFC models from large systems to fine electrodes. This logic helps to optimize the electrode structure to achieve the goals of system performance and durability. In addition, a top–down approach follows from simple to complex. Most large system models of SOFCs involve thermodynamic energy conversion or simple parameter distribution modeling, while electrode models involve complex reaction-transfer modeling. This simple to complex review process is beneficial to reader understanding.

Currently, SOFC models have been developed for macroscopic systems, macroscopic cells/stacks, and mesoscopic electrodes. Macroscale system models aim to elucidate the coupling and matching mechanisms of energy and species flow between different components, with the goal of optimizing system performance and developing efficient and clean energy conversion systems based on SOFCs. Macroscopic cell/stack models focus on clarifying the internal complex reaction-transport mechanisms to optimize the performance of SOFCs while ensuring stability and durability. These models are widely employed and highly relevant to SOFC manufacturing and operation, with abundant information available. Moreover, some commercial software, such as COMSOL and Fluent, have developed simulation modules at the cell/stack scale, making it easier to solve the model. Mesoscopic electrode models serve as a supplement and correction to cell/stack models, addressing issues that are difficult to explore at the macroscopic level, such as Ni particle coarsening, carbon deposition, and microscopic parameter extraction. These models provide deeper insight into the reaction mechanisms and microstructural evolution of porous electrodes, enabling more accurate simulation of electrode performance. However, mesoscopic models require expensive characterization instruments, advanced numerical theory, and long solution times. Notably, while the introduction of SOFC models is typically in the sequence of system–cell/stack–electrode, the practical development process of SOFCs should be the reverse. Only by starting with an excellent electrode can a cell/stack with excellent performance be produced, leading to a system with stable and efficient operation.

The combination of SOFC models and AI may be a hot research direction in the future. At present, some SOFC MPMs have been combined with ANNs. ANNs generally serve to build a surrogate model for MPMs or to replace the specific partial differential equation to decouple MPMs, thereby reducing their solution time. With the development of AI technology, the combination of SOFC models with DTs and GANs will likely become a hot topic in future research. DTs will enable the creation of a digital virtual operating process along with the real operating process so as to achieve online diagnosis and optimization of SOFCs without stopping the running process. This development is expected to greatly improve SOFC performance, reduce operational failures, and increase durability during runtime. GANs can be employed to generate mesoscopic 3D structures of SOFC electrodes, realizing fast and low-cost 3D heterogeneous model construction. Briefly, the assistance of AI will help to resolve problems that were previously difficult to solve via traditional models, thereby facilitating the rapid SOFC development.

Some new mesoscopic emerging simulation methods are also developing rapidly, which are of great significance in solving the problem in mesoscopic electrode scale. Despite necessitating interdisciplinary knowledge and posing significant challenges in resolution, these models exhibit remarkable efficacy in addressing specific mesoscopic-scale problems and are therefore worthwhile to pursue. However, most of these models are aimed at mesoscopic mechanism research. At present, the results obtained from these models are rarely applied to practical fields such as electrode structure, cell operating condition optimization, degradation performance improvement and so on. Therefore, cross-scale models need to be developed. By combining the characteristics of different scale models, SOFC can be studied from mesoscopic electrodes to macroscopic cells even to large systems. Only in this way can the mesoscopic electrode structure optimization results be successfully mapped to the cell/stack performance improvement, and the potential of SOFC performance can be deeply explored. Therefore, in the follow-up work, the cross-scale model will also become an attractive research direction.

The complexity of SOFC models highlights the importance of selecting the appropriate model to address specific research problems. While a more microscopic or precise model may offer greater accuracy than a mesoscopic model, it also increases the complexity and computational time. For instance, applying a stack-scale model to system-scale research may enhance accuracy but could lead to difficulties in solving the entire system model or even render it unsolvable. In such cases, the primary goal of analyzing and optimizing overall system performance cannot be achieved. This review has categorized and summarized models at different scales, providing typical application scenarios for different types of models. As such, this review serves as a valuable tool to assist researchers in selecting the most suitable model for their research objective.

The construction of most models is widely acknowledged to be based on certain assumptions, indicating that they are not flawless. Moreover, the existing modeling work does not fully address the problems that already exist. Consequently, existing models continuously warrant improvement. This review offers a comprehensive analysis of current SOFC models, identifying their limitations and areas that warrant further development. Additionally, this review outlines future directions for the development of SOFC simulations. The limitations of the existing works, the problems that need to be further solved, and the direction of development are summarized after the discussion of each kind of model. It is hoped that the insights provided herein will motivate future researchers to address the shortcomings of current SOFC models, thereby advancing SOFC technology.
